# OBO-Fused Benzo[fg]tetracene as Acceptor With Potential for Thermally Activated Delayed Fluorescence Emitters

**DOI:** 10.3389/fchem.2020.563411

**Published:** 2020-09-30

**Authors:** Zhen Zhang, Shiv Kumar, Sergey Bagnich, Eduard Spuling, Fabian Hundemer, Martin Nieger, Zahid Hassan, Anna Köhler, Eli Zysman-Colman, Stefan Bräse

**Affiliations:** ^1^Institute of Organic Chemistry (IOC), Karlsruhe Institute of Technology (KIT), Karlsruhe, Germany; ^2^Organic Semiconductor Centre, EaStCHEM School of Chemistry, University of St Andrews, St Andrews, United Kingdom; ^3^Soft Matter Optoelectronics, Bayreuth Institute for Macromolecular Research (BIMF) & Bavarian Polymer Institute (BPI), University of Bayreuth, Bayreuth, Germany; ^4^Department of Chemistry, University of Helsinki, Helsinki, Finland; ^5^Institute of Biological and Chemical Systems – Functional Molecular Systems (IBCS-FMS), Karlsruhe Institute of Technology (KIT), Eggenstein-Leopoldshafen, Germany

**Keywords:** boron emitters, fluorescence, OLED, TADF, boron

## Abstract

Six luminophores bearing an OBO-fused benzo[fg]tetracene core as an electron acceptor were designed and synthesized. The molecular structures of three molecules (PXZ-OBO, 5PXZ-OBO, 5DMAC-OBO) were determined by single crystal X-ray diffraction studies and revealed significant torsion between the donor moieties and the OBO acceptor with dihedral angles between 75.5 and 86.2°. Photophysical studies demonstrate that blue and deep blue emission can be realized with photoluminescence maxima (λ_PL_) ranging from 415 to 480 nm in mCP films. The emission energy is modulated by simply varying the strength of the donor heterocycle, the number of donors, and their position relative to the acceptor. Although the DMAC derivatives show negligible delayed emission because of their large singlet-triplet excited state energy difference, Δ*E*_ST_, PXZ-based molecules, especially PXZ-OBO with an experimental Δ*E*_ST_ of 0.25 eV, demonstrate delayed emission in blend mCP films at room temperature, which suggests triplet exciton harvesting occurs in these samples, potentially by thermally activated delayed fluorescence.

## Introduction

As one of the most promising electroluminescent technologies, organic light-emitting diodes (OLEDs) have attracted significant attention and are now being commercialized across a number of different product lines (Endo et al., 2011; Uoyama et al., 2012; Tao et al., 2014; Wong and Zysman-Colman, 2017; Yang et al., 2017). State-of-the-art phosphorescent OLEDs (PhOLEDs) have an Achilles heel, and that is the use of non-sustainable noble metal emitters. Purely organic thermally activated delayed fluorescence (TADF) OLEDs show comparable performance to PhOLEDs and use sustainable materials (Lin et al., 2016). TADF emitters rely mainly on a twisted donor acceptor structure (Chen et al., 2016; Wang et al., 2017). Heterocycles, such as carbazole derivatives (Albrecht et al., 2015), triphenylamines (Data et al., 2016), phenoxazines (Takahashi et al., 2014), and acridines (Zhang et al., 2016), are suitable donors to construct TADF molecules. Electron-accepting units, such as benzonitriles (Park et al., 2016), triazines (Kim et al., 2015), benzophenones (Lee et al., 2015), and sulfones (Lee et al., 2016), are commonly used as acceptors.

Three-coordinate boranes have also been reported as electron-withdrawing acceptors in donor–acceptor systems (Numata et al., 2015; Kitamoto et al., 2016; Chen et al., 2017; Lee et al., 2017). However, the high susceptibility of boron–carbon (B–C) bond cleavage in electron-rich and electron-poor BN heterocycles along with vacant p_z_-orbitals on the boron atom has resulted in poor chemical and photostability (Yang et al., 2016). For example, the BN heterocycles undergo photoelimination reaction to form a new π-conjugated polycyclic azaborine compounds (Lu et al., 2013; Yang et al., 2016). In order to increase the chemical and photostability of these materials, steric bulk is added in the vicinity of the boron atom, leading to air-stable derivatives. Suzuki and coworkers (Suzuki et al., 2015) synthesized two stable boron-containing compounds based on the bis(mesityl)borane (BMes_2_) acceptor and realized efficient sky-blue [Commission Internationale de l'Eclairage (CIE): 0.18, 0.43] and green (CIE: 0.22, 0.55) TADF OLEDs with maximum external quantum efficiency (EQE_max_) of 21.6% (2DAC-Mes_3_B) and 22.8% (PXZ-Mes_3_B), respectively. Positioning the boron atom within polycyclic π systems is another strategy to achieve improved stability of the emitter. With a demethylative direct borylation method, Hatakeyama et al. reported synthesis of stable benzo[fg]tetracenes core containing boronate ester, amide, and thioester substructures. Depending on the heteroatom fragment (O and NMe) adjacent to the boron, these materials exhibited characteristic emission in the UV at 335 to 377 nm (Katayama et al., 2016; Numano et al., 2016). Müllen et al. also reported a series of OBO-doped tetrabenzo[*bc,ef,kl,no*]coronenes and tetrabenzo[*a,f,j,o*]perylenes, which exhibited structured blue fluorescence with ϕ_PL_ of 61 and 27%, respectively (Wang et al., 2016). In 2019, Yasuda et al. reported two TADF emitters using boronate ester or boronate thioester as the acceptor (Matsuo and Yasuda, 2019). The boronate thioester–based device showed sky-blue emission at 489 nm (CIE: 0.17, 0.39) and excellent EL performance with the EQE_max_ of 20.9%. Although the boronate ester–based device exhibited only the EQE_max_ of 5.2% because of its low PLQY of 28%, its bluer emission at 471 nm (CIE: 0.17, 0.22) revealed the potential of the boronate ester acceptor for blue and even deep blue TADF emitters.

**Figure 1:**
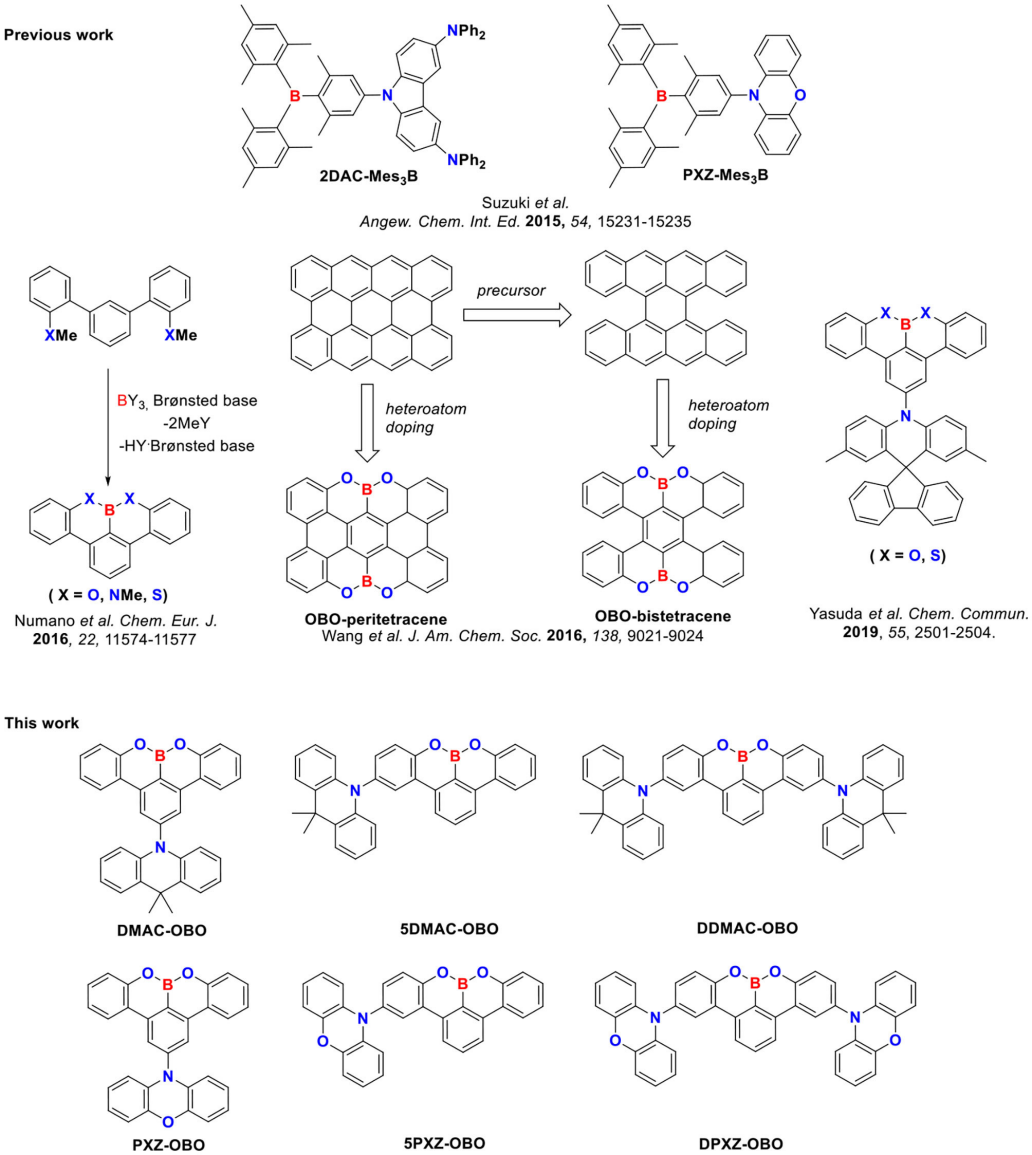
Chemical structures of boron-containing compounds.

Herein we report six examples of blue emitters based on this OBO-based benzo[fg]tetracene acceptor (OBO). We systematically investigate the impact of the number, identity of the donor (9,9-dimethyl-9,10-dihydroacridine, DMAC, and 10*H*-phenoxazine, PXZ), and their position relative to OBO on the optoelectronic properties of the emitter (Figure 1).

## Results and Discussion

### Synthesis

**Scheme 1:**
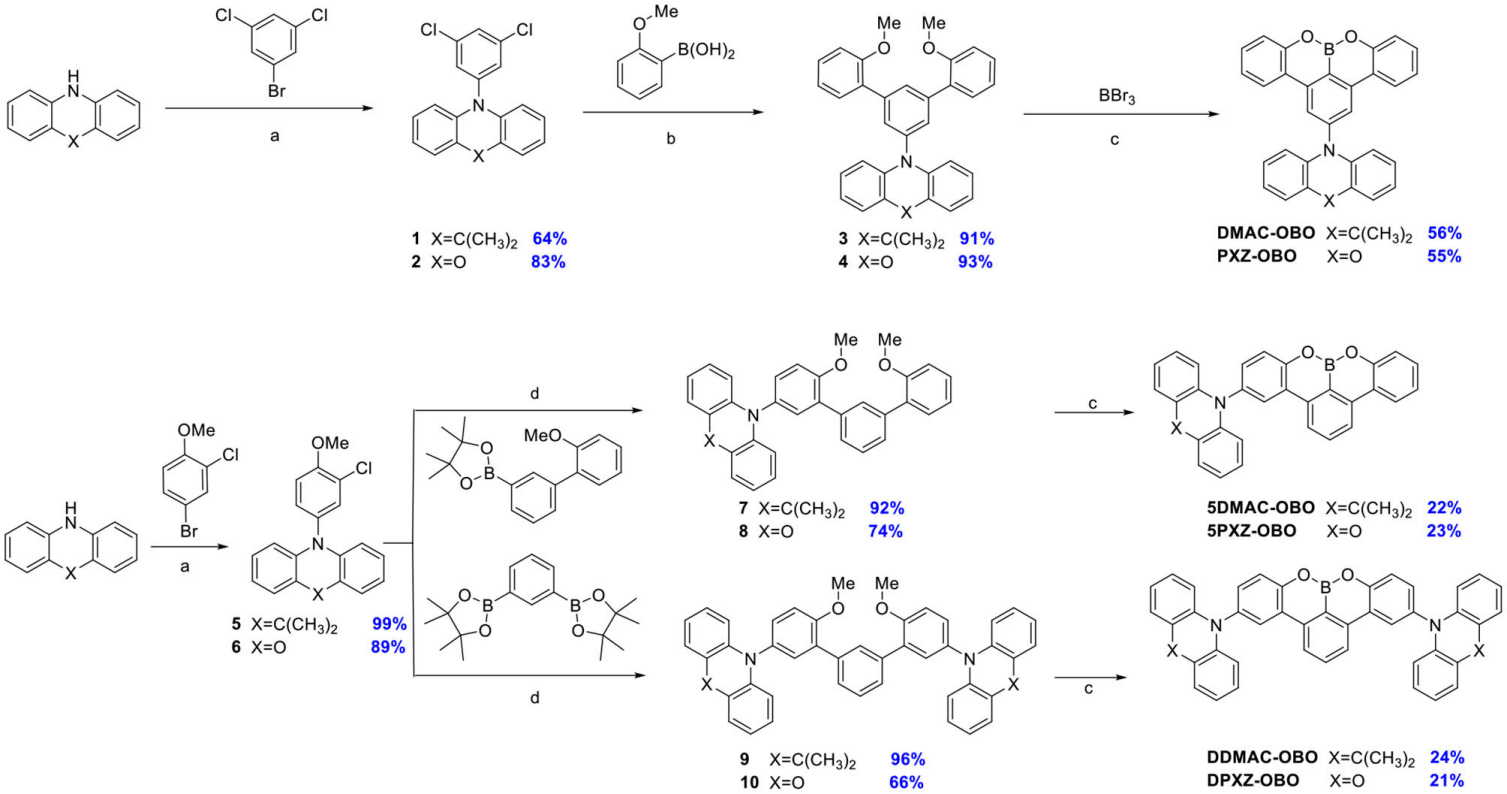
Synthetic route for OBO-based derivatives. a. Pd(OAc)_2_, xantphos, NaO^*t*^Bu, PhMe, 100°C, 12 h, argon; b. Pd(OAc)_2_, SPhos, K_3_PO_4_, THF/H_2_O, 80°C, 12 h; c. 150°C, 1,2-dichlorobenzene, 12 h, argon; d. Pd(OAc)_2_, SPhOS, K_3_PO_4_, PhMe, 100°C, 12 h.

These six OBO-based derivatives were successfully synthesized via a three-step procedure (Scheme 1). First, DMAC and PXZ were treated with halo-substituted anisoles under Buchwald–Hartwig cross-coupling conditions to afford the corresponding coupled products **1**, **2**, **5**, and **6** in good to excellent yield. These intermediates were reacted with arylboronic acids/esters under Suzuki–Miyaura cross-coupling conditions to produce dimethoxyteraryl intermediates (**3**, **4**, **7**–**10**) in good yields. Finally, the target OBO-based emitters were obtained through a demethylative direct borylation procedure of the aryl methyl ethers in the presence of BBr_3_ (Wang et al., 2016). This final cyclization proceeded in 55 to 56% yield (DMAC-OBO and PXZ-OBO) when the donor units were introduced to the central phenyl ring. On the other hand, the yields dropped to 21 to 24% when the donor units were in *para* position to the methoxy group. The final products were found to be easily purified by silica gel column chromatography and stable under ambient conditions for more than 2 years, showing no decomposition in nuclear magnetic resonance spectra.

**Figure 2:**
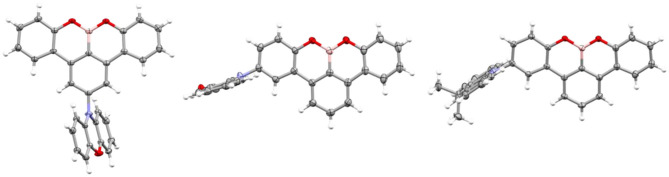
Molecular structure of **PXZ-OBO** (left, CCDC 1973638), **5PXZ-OBO** (middle, CCDC 1973639), and **5DMAC-OBO** (right, CCDC 1973640, one of the two crystallographically independent molecules is shown) drawn at 50% probability level.

The crystals of PXZ-OBO, 5PXZ-OBO, and 5DMAC-OBO suitable for single crystal X-ray diffraction analysis were obtained from a mixed solution of dichloromethane (DCM) and cyclohexane. The structures reveal that these three molecules possess the expected highly twisted donor–acceptor conformation (Figure 2). The dihedral angles between donor and acceptor for the two structural isomers PXZ-OBO and 5PXZ-OBO are tuned remarkably. For PXZ-OBO, this torsion is 75.5°, whereas the donor–acceptor dihedral angle is larger at 86.2° for 5PXZ-OBO. The DMAC analog, 5DMAC-OBO, shows a slightly reduced twisting angle between the donor and the acceptor at 80.1°. In all the structures, the OBO-containing acceptor itself is flat.

### Theoretical Calculations

**Figure 3:**
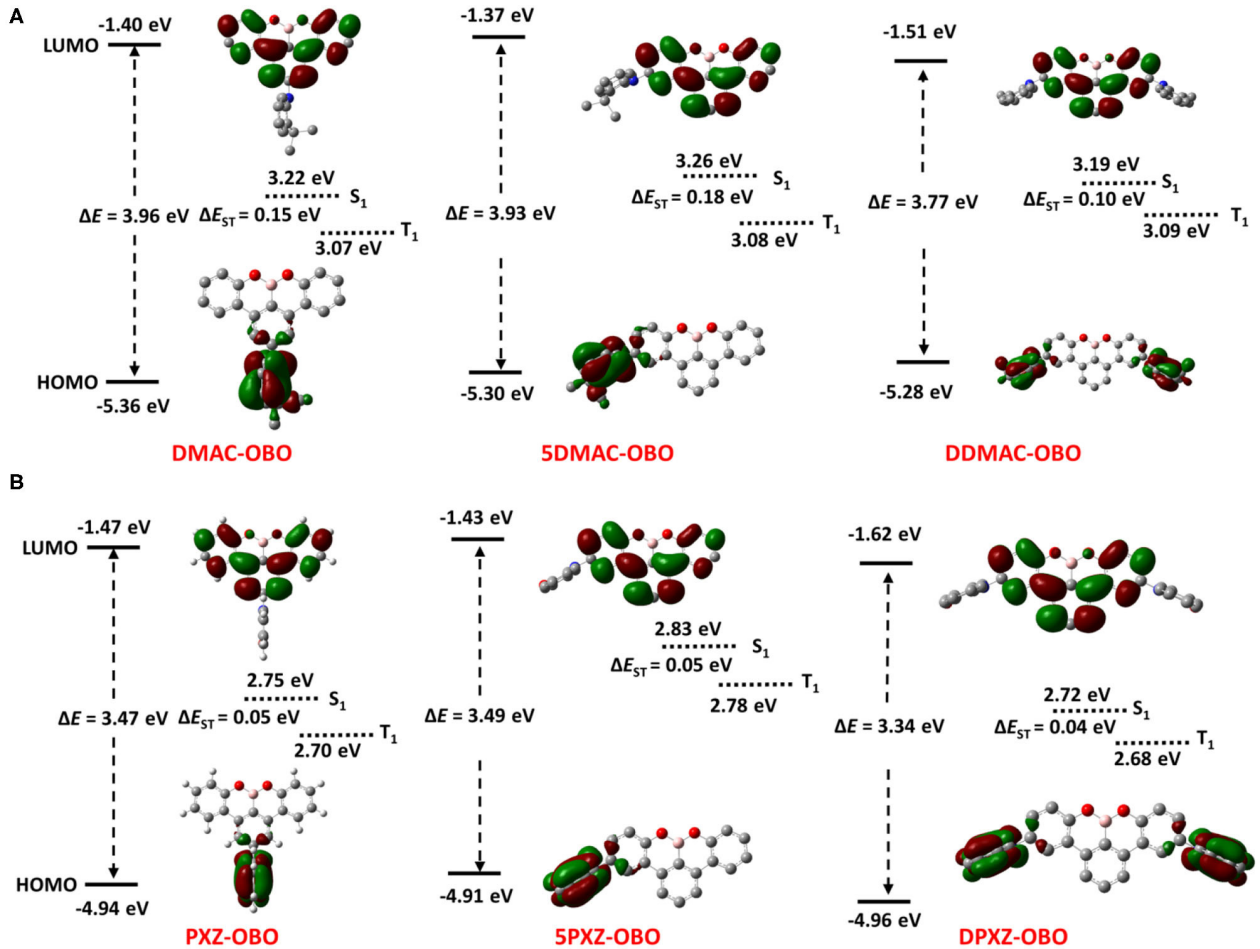
DFT calculated ground and excited state energies and electron density distributions of the HOMOs and LUMOs for **(A)** DMAC-based OBO emitters and **(B)** PXZ-based OBO emitters. The orbitals are derived using the ground state geometry.

Density functional theory (DFT) calculations were performed to assess the electronic structure of the six emitters using the PBE0 (Adamo and Barone, 1999) functional and the 6–31G(d,p) basis set (Cancès et al., 1997) implemented within Gaussian 09 (Frisch et al., 2009). The ground state geometries of the molecules were optimized in the gas phase starting with the geometry obtained from the single crystal X-ray diffraction analysis. Time-dependent DFT calculations were performed within the Tamm–Dancoff approximation (TDA) on the ground state optimized molecular structures (Hirata and Head-Gordon, 1999). The energies and electron density distributions of the highest occupied and lowest unoccupied molecular orbitals (HOMO/LUMO) and the energies of the S_1_ and T_1_ states are shown in Figure 3, and the data are summarized in Tables 1, 2, and Supplementary Table 1, and the electron density distributions of other molecular orbitals are summarized in Supplementary Table 2. As indicated in the SI, the HOMO → LUMO transition is the dominant contribution to the S_1_ state.

**Table 1:** Summary of electrochemical and DFT/TDA calculated photophysical properties of OBO-based emitters.

**Emitters**	**Experimental**			**Calculated**	
	**$E_{pa,1}^{ox}a$/$E{\mathrm{red}}_{pc,1}^{b}$ (V)**	**HOMO/LUMO (eV)**	**Δ*E*^c^ (eV)**	**HOMO^d^/LUMOd (eV)**	**Δ*E*^c^7 (eV)**
DMAC-OBO	0.98/−2.11	−5.78/−2.69	3.09	−5.36/−1.40	3.96
5DMAC-OBO	0.97/−1.84	−5.77/−2.96	2.81	−5.30/−1.37	3.93
DDMAC-OBO	0.97/−1.87	−5.77/−2.93	2.84	−5.28/−1.51	3.77
PXZ-OBO	0.70/−2.10	−5.50/−2.70	2.80	−4.94/−1.47	3.47
5PXZ-OBO	0.75/−1.98	−5.55/−2.82	2.73	−4.91/−1.43	3.48
DPXZ-OBO	0.79/−1.81	−5.59/−2.99	2.60	−4.96/−1.62	3.34

a In CH_2_Cl_2_ and

b *in DMF, with 0.1 M [nBu_4_N]PF_6_ as the supporting electrolyte, Pt as the working electrode, Ag/AgCl as the reference electrode and Pt wire as the counter-electrode. Fc/Fc^+^ was used as the internal reference and the data reported vs. SCE. The HOMO and LUMO energies were calculated using the relation E_HOMO_/_LUMO_ = –($E_{pa,1}^{ox}$/$E_{pc,1}^{red}$ + 4.8) eV, where $E_{pa}^{ox}$ and $E_{pc}^{red}$ are anodic and cathodic peak potentials, respectively*.

c *ΔE = |E_HOMO_ – E_LUMO_|*.

d *Determined from the DFT or TDA-DFT calculations*.

**Table 2:** S_1_ and T_1_ energies determined in 2-MeTHF solution At 77 K and from theoretical calculation.

**Compounds**	**S_**1**_(77 K) (eV)**	**$S_{1}^{\text{theor}}$ (eV)**	**T_**1**_(77 K) (eV)**	**$T_{1}^{\text{theor}}$ (eV)**	**Δ*E*_**ST**_(77 K) (eV)**	**$\Delta E_{\text{ST}}^{\text{theor}}$ (eV)**
DMAC-OBO	3.43	3.22	2.69	3.07	0.74	0.15
5DMAC-OBO	3.60	3.26	2.69	3.08	0.91	0.18
DDMAC-OBO	3.51	3.19	2.68	3.09	0.83	0.10
PXZ-OBO	3.34	2.75	2.70	2.70	0.64	0.05
5PXZ-OBO	3.34	2.83	2.70	2.78	0.64	0.05
DPXZ-OBO	3.33	2.72	2.69	2.68	0.64	0.04

The near orthogonal dihedral angle between the donor and acceptor units in the ground state geometries matched with the single crystal data analysis. The HOMO of the emitters is localized on the donor DMAC or PXZ groups, while the LUMO of the emitters is localized on the electron-withdrawing OBO units. There is minimal spatial overlap between the HOMO and LUMO, which suggests a strong CT character of the excited singlet state and which results in a small calculated Δ*E*_ST_ for the ground state geometry. The S_1_ energies for the DMAC series ranged narrowly from 3.22 to 3.19 eV, showing potential as blue emitters. The use of the stronger PXZ donor results in smaller Δ*E*_ST_ values and lower S_1_ energies (2.72–2.83 eV) compared to the DMAC analogs; the use of two donor units in DDMAC-OBO and DPXZ-OBO also contributed to decreased Δ*E*_ST_. Even though these values are derived for the vertical transition from the ground state geometry, the calculated Δ*E*_ST_ values for all emitters are sufficiently small to justify their further evaluation as TADF emitters.

### Electrochemical Properties

**Figure 4:**
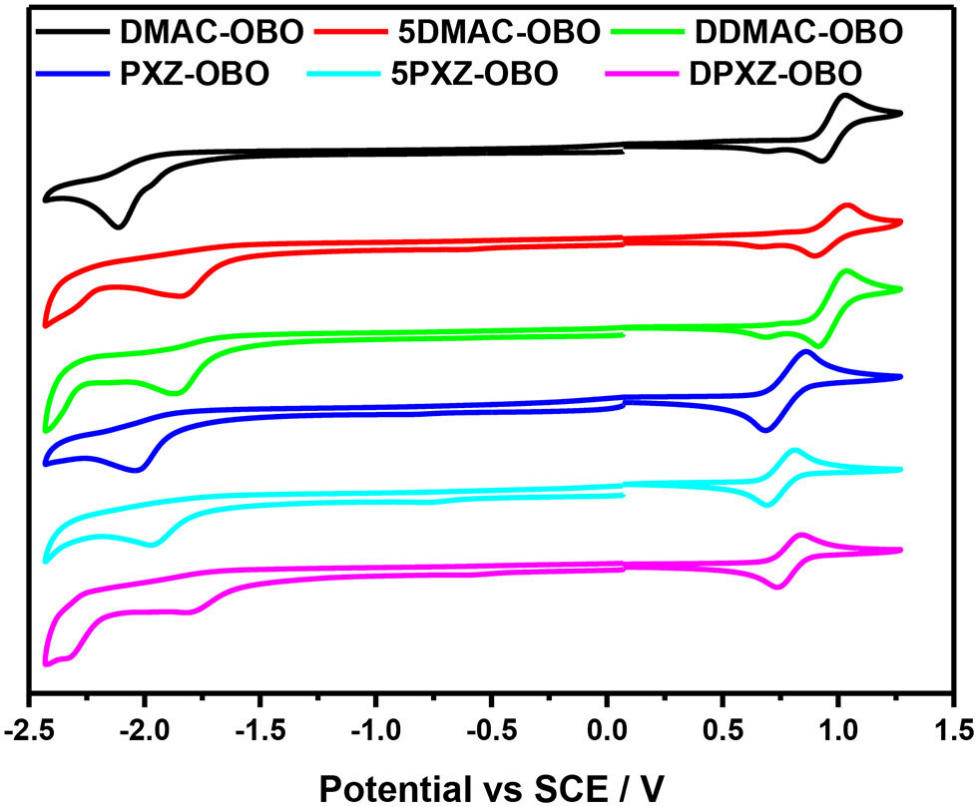
Cyclic voltammograms of OBO-based emitters (reduction and oxidation scans were carried out in degassed dimethylformamide (DMF) and DCM, respectively, at a scan rate 100 mV s^−1^).

The electrochemical properties of the six emitters were examined by cyclic voltammetry (CV) as shown in Figure 4, and the results are listed in Table 1. The reduction wave was assigned to the electron-accepting OBO unit and found to be irreversible. The LUMO value for DMAC-OBO, 5DMAC-OBO, and DDMAC-OBO were determined to be −2.69, −2.96, and −2.93 V, respectively, from the reduction potentials obtained by CV. For the PXZ analogs PXZ-OBO, 5PXZ-OBO, and DPXZ-OBO, the LUMO values were determined to be −2.70, −2.82, and −2.99 V, respectively. The LUMO of 5DMAC-OBO is lowest among DMAC-based emitters, whereas the most stabilized LUMO in the PXZ family of compounds is DPXZ-OBO. The oxidation wave was found to be reversible in all six emitters, which demonstrates that PXZ and DMAC radical cations are electrochemically stable. The DMAC-based emitters show slightly more anodic oxidation potentials than the corresponding PXZ-based emitters, in line with their relative electron-donating ability.

### Photophysical Properties

The newly synthesized OBO derivatives form two structurally analogous series. While the OBO moiety always takes the acceptor role, in the first series, DMAC is used as donor moiety, and PXZ is used as donor for the second series. We address here how changing the number and position where the donor is attached impacts on the electronic structure of the TADF molecule.

#### Absorption and Emission in Solutions

**Figure 5:**
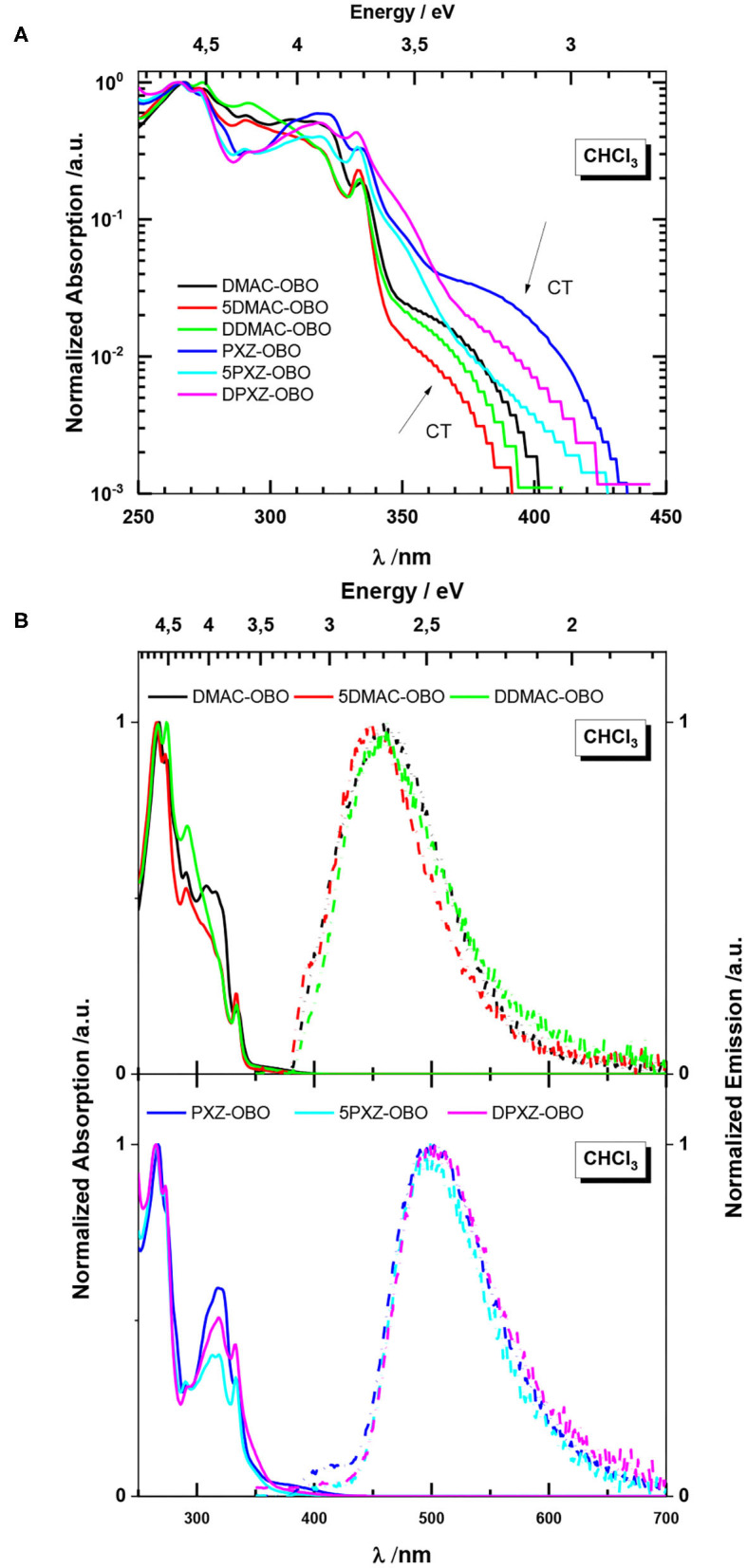
**(A)** Absorption in CHCl_3_ on semilogarithmic scale. **(B)** Absorption (left) and photoluminescence spectra (right) in CHCl_3._ (λ_exc_ = 332 nm).

The absorption spectra of the emitters in CHCl_3_ are shown in Figure 5A. In solution, all the emitters exhibit similar absorption profiles with the absorption peaks between 260 and 340 nm. Taking into account the structureless absorption spectra of the donors, one can assign these maxima to localized π-π^*^ transitions of the acceptor OBO moiety (Numano et al., 2016). In the case of DMAC molecules, the absorption of the donor is masked by absorption of the acceptor and cannot be clearly observed (Rodella et al., 2020). In contrast, for the PXZ compounds, the contribution of the donor is distinguished as the shoulder at 350 nm (Mantsch, 1969). On the semilogarithmic scale, weak broad structureless absorption bands can be seen in the low-energy part of the spectra. These broad bands are ascribed to an intramolecular charge transfer transition that corresponds to transfer of electron from the PXZ or DMAC donor to the OBO acceptor, in agreement with the theoretical calculations. In the case of the PXZ molecules, this band is shifted to longer wavelengths by 25 nm (0.2 eV).

**Figure 6:**
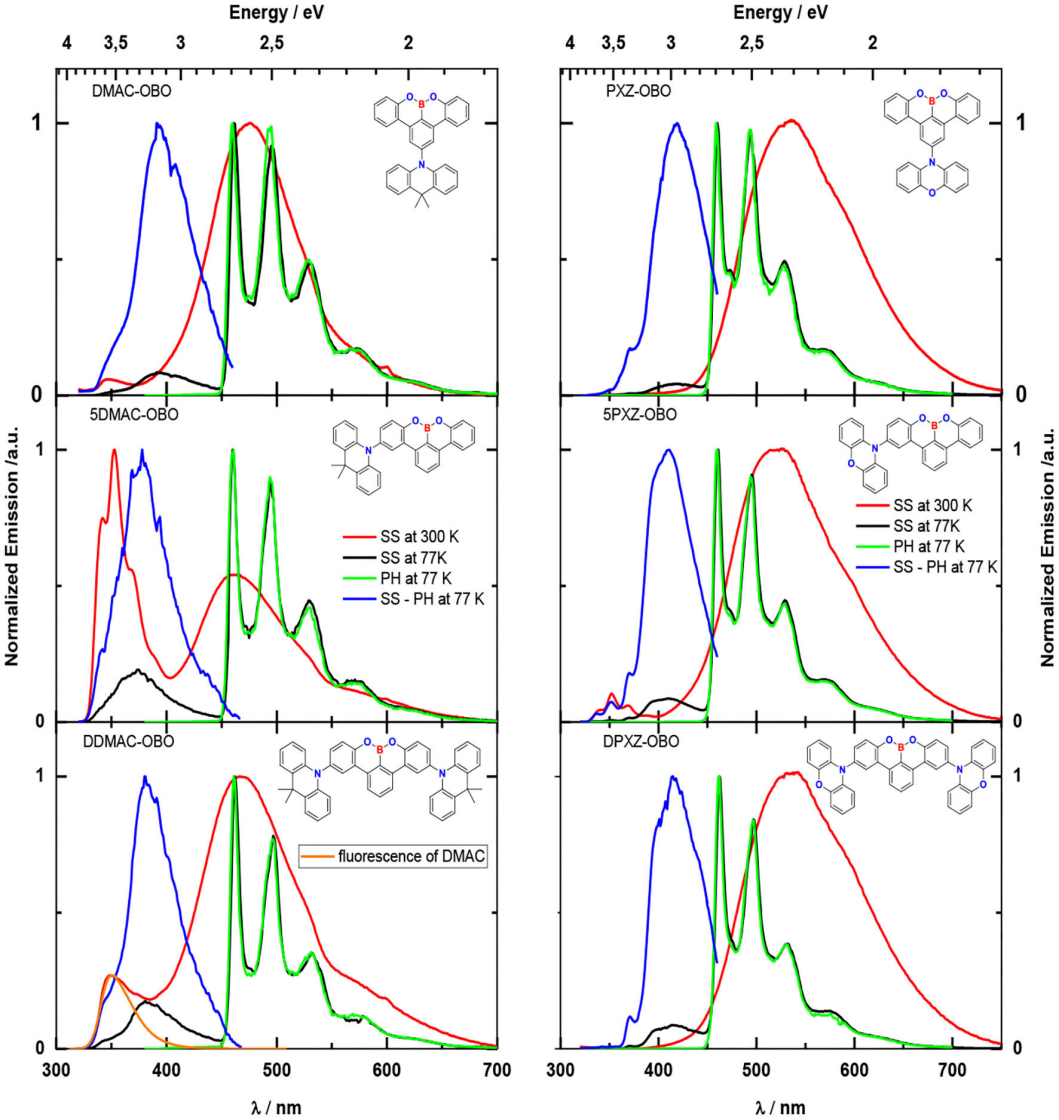
SS emission in 2-MeTHF at 300 K (red lines), SS emission (black lines), phosphorescence (green lines), fluorescence of OBO-based emitters (blue lines) in 2-MeTHF at 77 K. The fluorescence spectrum was obtained by subtracting the phosphorescence from the SS spectrum and by next normalizing. Excitation was at 300 nm. The concentration was 0.05 mg/mL. The orange line corresponds to DMAC fluorescence at 300 K.

The photoluminescence (PL) spectra are broad and unstructured, an indication of emission from a CT state (Figure 5B). The PL spectra of the DMAC-based emitters in CHCl_3_ show blue–green emission with maxima (λ_PL_) between 448 and 460 nm, whereas for the PXZ-based emitters, a red-shifted emission is observed maxima ranging more narrowly between 497 and 501 nm. The weak bands observed for PXZ-containing molecules can be assigned to emission of the donor (see discussion of Figure 6).

Steady-state (SS) emission in 2-methyltetrahydrofuran (2-MeTHF) at 300 and 77 K was investigated to explore the energy of the excited states of the emitters (Figure 6 and Table 2). At 300 K, all molecules demonstrate a broad emission spectrum centered at about 2.6 eV (DMAC-containing compounds) or 2.3 eV (PXZ-containing compounds) that is close to the, respectively, spectrum observed in CHCl_3_. This band can be assigned to a transition from lowest stabilized CT state. Concomitantly, when exciting the solutions at 300 nm for the DMAC-containing molecules, a second, high-energy band is observed with a λ_PL_ at 350 nm. For the more symmetric molecules DMAC-OBO and DDMAC-OBO, this band is not structured, while it carries additional pronounced shoulders/peaks for the less symmetric 5DMAC-OBO. The unstructured band fits well in terms of the shape and position to the spectrum of DMAC fluorescence and can therefore be assigned to emission from an LE state on the acridine donor (the emission spectrum of DMAC has been inserted in Figure 6 for DDMAC-OBO, orange line, for ease of reference). The pronounced features in the 5DMAC-OBO exactly match the energies and linewidth reported from Numato et al. at 3.65, 3.50, 3.35, and 3.20 eV for the vibronic progression of the emission associated with the OBO-acceptor moiety (Numano et al., 2016). Evidently, for the less symmetric 5DMAC-OBO compound, there is LE emission predominantly from the acceptor, in contrast to the LE emission from only the donor for the two more symmetric compounds. For the PXZ compounds, we also observe the characteristic vibronically well-resolved emission from the OBO acceptor for the less symmetric 5PXZ-OBO, whereas it is not present in the more symmetric PXZ-OBO and DPXZ-OBO.

Dual emission is not a common phenomenon in general, as internal conversion (IC) from the higher to the lower excited state usually competes efficiently with radiative decay from the higher state to the ground state, unless there is a large energy gap separating the two emitting state as, e.g., for azulene. However, such dual emission from a CT and an LE state has already previously been observed for donor–acceptor compounds (Acar et al., 2003; Druzhinin et al., 2015). In such molecules, IC from LE to CT state corresponds to electron transfer between two parts of the molecule. Because of the nearly perpendicular conformation of the donor and acceptor, the initial intramolecular electron transfer can be sufficiently slow so that radiative decay from the LE states may compete with the IC process.

In both series, the intensity of the CT emission relative to the respective LE emission decreases in the order DMAC-OBO (PXZ-OBO), DDMAC-OBO (DPXZ-OBO), and 5DMAC-OBO (5PXZ-OBO). This order reflects the relative strength of the y-component of the molecular transition dipole moment that would result in a simple picture where the whole density is considered localized on the nitrogen and the electron density on the boron.

The SS PL spectra at 77 K consist of a strong and highly structured phosphorescence and a weak fluorescence emission. The transition in the SS spectra from 300 to 77 K leads to a large shift of the fluorescence of the molecules to higher energy (to 3.2 eV for the DMAC compounds and to 3.0 eV for the PXZ compounds). This is caused by the freezing out of the reorientation of solvent shell molecules, so that reorganization of the emitter after excitation is precluded (Lakowicz, 2006). As for 300 K, the 77 K fluorescence bears the signature of the respective LE states. We point out that the vibrational structure that weakly superimposes on the CT emission for PXZ-OBO and DPXZ-OBO is not that of the OBO acceptor but rather that of the phenoxazine donor LE (Mantsch, 1969), whereas that in 5PXZ-OBO is again the LE state of the OBO acceptor.

The phosphorescence observed has the same position and the same shape for all six molecules, implying its origin is from a purely LE triplet state of the OBO acceptor (Matsuo and Yasuda, 2019). Table 2 presents the values for the singlet and triplet energies and the corresponding Δ*E*_ST_ values. The energy of the triplet and singlet states was obtained from the position of the onsets of the phosphorescence spectra (Figure 6, green lines) and fluorescence spectra (Figure 6, blue lines), respectively. Table 2 shows that the theoretical values for the singlet state energy more or less coincide with experimentally derived values for the DMAC-based molecules, whereas for PXZ-based molecules, the theoretical data are very close the experimental one for the triplet state. Nevertheless, the experimental values for Δ*E*_ST_ (0.64–0.91 eV) are significantly higher than the theoretical prediction (0.04–0.18 eV) for both types of molecules, the latter conducted in the gas phase.

#### Luminescence in Blend mCP Films

**Figure 7:**
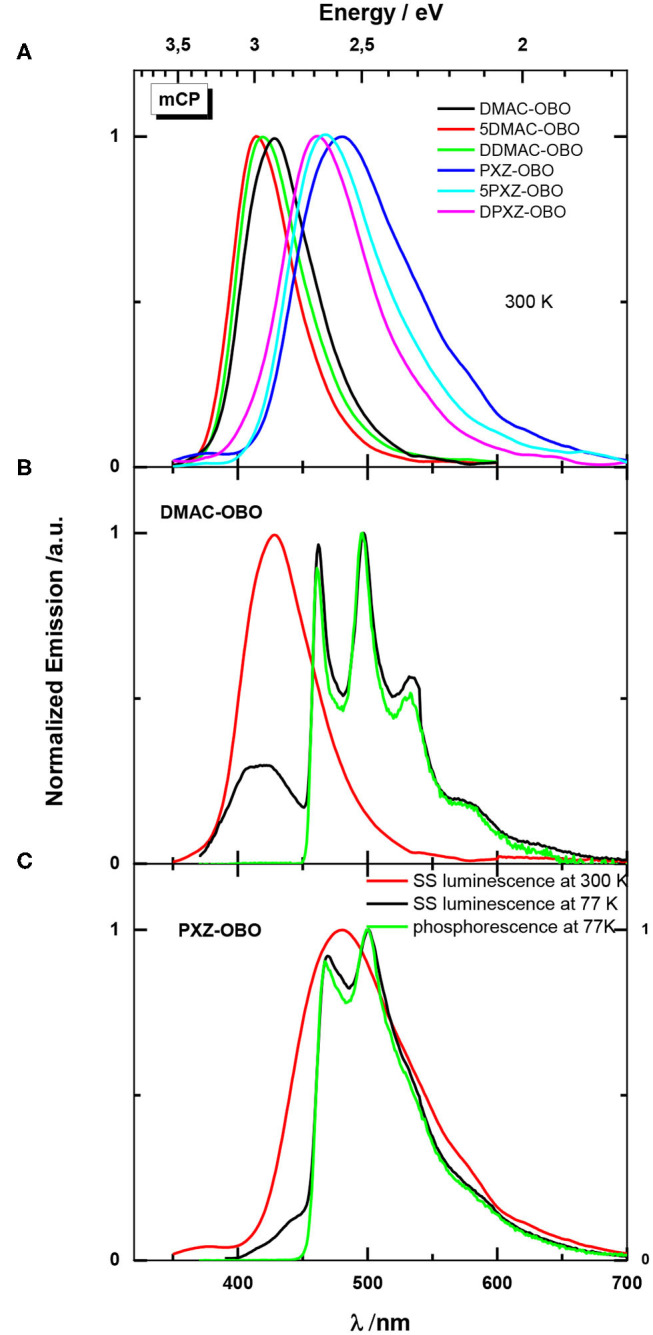
**(A)** PL spectra in 10 wt% doped films in mCP at 300 K. **(B)** PL spectra in 10 wt% doped films of **DMAC-OBO** in mCP at 300 and 77 K. **(C)** PL spectra in 10 wt% doped films of **PXZ-OBO** in mCP at 300 and 77 K. Excitation 320 nm.

While the solution data are relevant to analyze the excited states of the molecule, for electroluminescence applications the solid-state host materials doped with the emitter need to be considered. So, the PL behavior of these emitters in mCP films was studied at 300 K (Figure 7). The DMAC-based derivatives show deep blue emission with λ_PL_ ranging from 415 to 428 nm, whereas the emission of the PXZ-based derivatives is red-shifted and blue to sky-blue with λ_PL_ ranging from 458 to 480 nm. Like for measurements in 2-MeTHF glass at 77 K, phosphorescence dominates in emission of the mCP blend films at 77 K. The phosphorescence of the compounds in the DMAC series coincides with the phosphorescence observed in 2-MeTHF glass, implying that this emission corresponds to acceptor phosphorescence. However, for PXZ-based molecules, the phosphorescence differs in shape and position from measurements in 2-MeTHF glass. In fact, this phosphorescence in the shape and position coincides with phosphorescence of the donor (Mantsch, 1969). This implies that for PXZ-based molecules, there are two LE triplet states that are very close in energy. This is not the case for DMAC-based molecules, where donor triplet state has an energy of around 3.1 Ev (Rodella et al., 2020). The relative position of these lowest triplet states can vary, depending on the host properties. The weak fluorescence at 77 K is close in position to that observed at 300 K (Figures 7B,C). The energy of the CT singlet states in the films is lower in comparison with that measured in the 2-MeTHF glass at 77 K. This effect can be explained by considering that dispersion effects are negligible for small-molecular solvents but can be remarkable in the case of adjacent host molecules with an extended π-electron system as is the case with mCP. Thus, after excitation, the resulting change of dipole moment of the emitter leads to a change of electron distribution in the host molecule, which in turn leads to stabilization of the excited state of the dopant.

#### Transient Luminescence in mCP Blend Films

**Figure 8:**
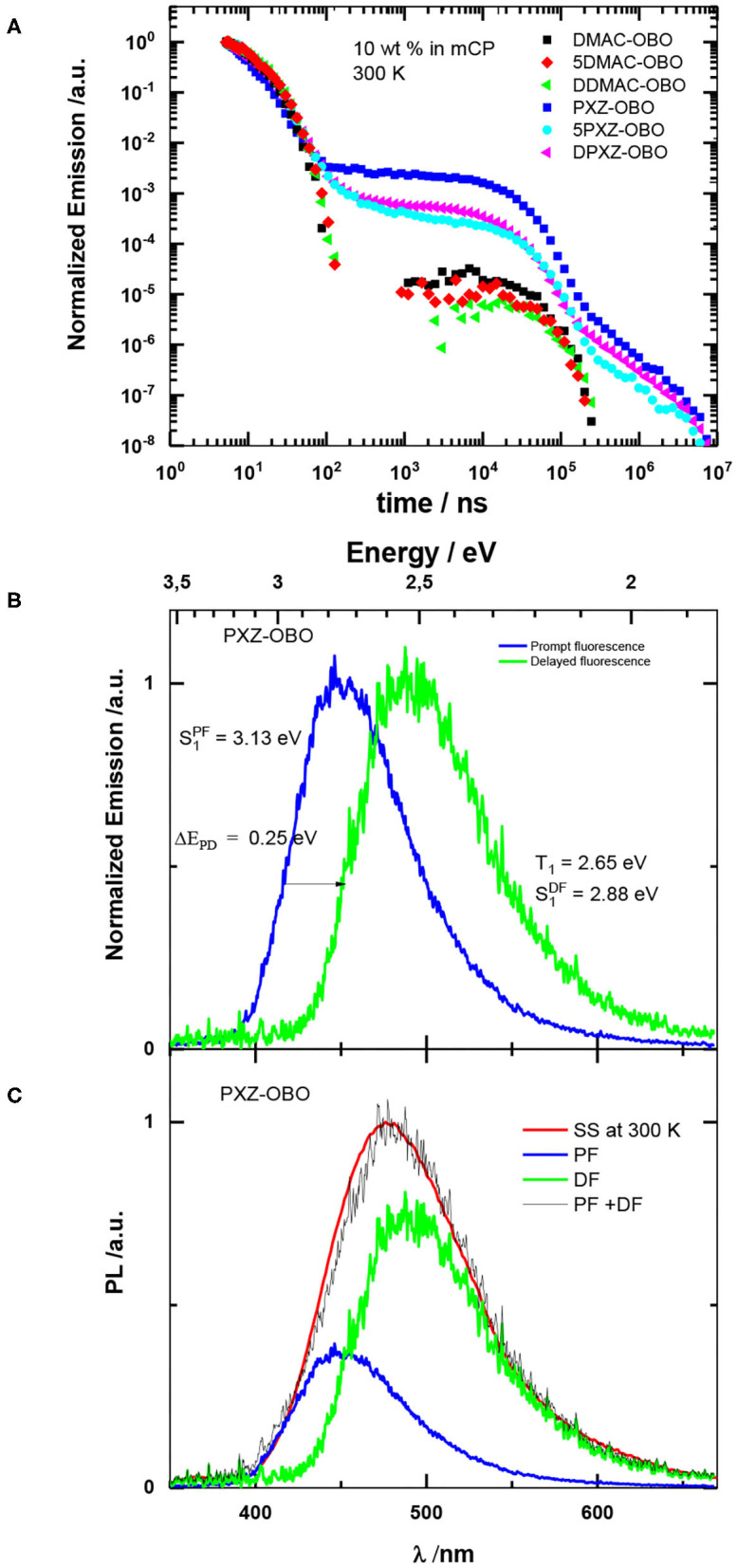
**(A)** Transient data for blend films in mCP at 300 K. **(B)** Spectra of prompt (delay 10 ns, gate 10 ns) and delayed fluorescence (delay 1 μs, gate 100 ns) of **PXZ-OBO** in mCP film at 300 K. **(C)** Analysis of the spectrum of total emission of PXZ-OBO in mCP film using the spectra of prompt and delayed components from **(A)**.

To allow for an analysis of the TADF properties, Figure 8A presents the transient decay of the emission of the molecules under investigation in 10 wt% mCP blend films at 300 K. Considering the large Δ*E*_ST_ for DMAC-based molecules (Table 2), we do not expect any considerable TADF for these compounds and that is confirmed by the experimental data. For PXZ-based molecules, delayed emission is clearly observed, and its contribution is surprisingly high, with a *I*_DF_/*I*_PF_ between 0.6 and 4. To explore whether this DF is due to TADF or TTA, we observed its dependence on the intensity of the exciting laser beam. As it is seen from Supplementary Figure 4, this dependence demonstrates a lineal character with slope value close to 1 that points out to monomolecular nature of this emission.

An analysis of the time-resolved spectra helps to understand the mechanism of TADF in case of such large singlet-triplet gap. Figure 8B shows the spectra of the prompt and delayed fluorescence. The DF spectrum is shifted to lower energy by 0.25 eV relative the PF spectrum; i.e., it results only from the lower energy part of the DOS. Such a broad DOS distribution can result in solid solutions where there are a number of different conformers of donor–acceptor molecules, each associated with different energies of their excited states (Penfold et al., 2018). Figure 8B demonstrates clearly that only molecules with the lowest singlet state energy (the low energy tail of the DOS) and lower singlet-triplet gap energy (0.25 eV) contribute in TADF. One can see from Figure 8C that the spectrum of the SS emission can be well-fitted by a superposition of the spectra of prompt and delayed fluorescence. It is clear that maximum of the SS spectrum and its intensity is determined by TADF, whereas PF forms high-energy tail of the SS spectrum.

#### Luminescence in Blend PMMA Films

Unfortunately, the PLQY values for these six compounds in mCP films are low, ranging from 5 to 15%. Therefore, PMMA as host materials was also investigated to explore the emission and quantum yield of these emitters in a second solid state host material. In 10 wt% doped PMMA films (Supplementary Figure 5), the PXZ-based derivatives show sky-blue emission with λ_PL_ ranging from 471 to 491 nm, whereas the emission of the DMAC-based derivatives is deep blue with λ_PL_ ranging from 410 to 446 nm. When the donor is decorated on the central phenyl ring of the OBO unit, the emission of PXZ-OBO is 491 nm, whereas it is 481 nm for 5PXZ-OBO with the donor located at the *para* position to the methoxy group. The corresponding DMAC derivatives demonstrate a similar trend. This trend can be explained by the mesomeric donation of the donor to the oxygen atoms, thereby strengthening the acceptor. The PLQYs of all three DMAC derivatives remain low, between 8 and 10%. In contrast, the family of PXZ derivatives shows higher PLQYs, from 19 to 46% (Table 3).

**Table 3:** Photophysical properties of OBO-based emitters.

**Compounds**	**$\lambda_{\text{PL}}^{\text{a}}$ (nm)**	**$\lambda_{\text{PL}}^{\text{b}}$/$\lambda_{\text{PL}}^{\text{c}}$ (nm)**	**FWHM^c^ (eV)**	**CIE(*x*, *y*)^c^**	**PLQY*^b^*/PLQY*^c^* (%)**	**τ_p_^b^ (ns)**	**τ_d_^b^ (μs)**	***I*_**DF**_/$I_{\text{PF}}^{\text{b}}$**
DMAC-OBO	460	429/446	0.56	0.16, 0.13	5/8	8	37	0.07
5DMAC-OBO	448	415/410	0.53	0.17, 0.10	9/8	9	42	0.04
DDMAC-OBO	461	419/420	0.55	0.16, 0.11	8/10	10	57	0.03
PXZ-OBO	500	480/491	0.49	0.21, 0.36	15/46	6	23	4
5PXZ-OBO	497	466/471	0.52	0.18, 0.24	7/19	10	30	0.6
DPXZ-OBO	501	462/481	0.50	0.20, 0.31	9/20	11	22	1

a *In CHCl_3_ at 300 K*.

b *In 10 wt% doped films in mCP at 300 K*.

c *In 10 wt% doped films in PMMA at 300 K. τ_p_, prompt lifetime. τ_d_, delayed lifetime*.

## Conclusions

We report six blue emitters employing an OBO-fused benzo[fg]tetracene core as an acceptor. Experimental studies demonstrate their blue and deep blue emission with λ_PL_ of 415 to 480 nm in mCP films. Although the DMAC derivatives show the negligible delayed emission (*I*_DF_/*I*_PF_, 0.03–0.07), the PXZ-based emitters exhibit much higher ratio of delayed emission (*I*_DF_/*I*_PF_, 0.6–4) with the PLQYs from 5 to 15%. When doped in PMMA, these fluorescent emitters show higher PLQYs, from 19 to 46%. Current efforts are focused on designing new OBO-based molecules through exploring different donors and novel connectivity strategies between donor and OBO acceptor to access more promising blue TADF emitters for OLEDs.

## Data Availability Statement

The datasets presented in this study can be found in online repositories. The names of the repository/repositories and accession number(s) can be found in the article/Supplementary Material. The research data supporting this publication can be accessed at https://doi.org/10.17630/5acd95b7-73bf-4e5a-abe6-ec23c9120835.

## Author Contributions

ZZ conceived the concept and synthesized and characterized the materials. SK performed the DFT calculations electrochemical measurements. SBa performed the photophysical measurements and, together with AK, wrote the photophysics section. ES, FH, and ZH participated in results discussions. MN did the X-ray analyses. ZZ, SK, and EZ-C wrote the manuscript. SBr, AK, and EZ-C supervised the project. All authors contributed to the article and approved the submitted version.

## Conflict of Interest

The authors declare that the research was conducted in the absence of any commercial or financial relationships that could be construed as a potential conflict of interest.
