# Supplementary material for: OBO-Fused Benzo[fg]tetracene as Acceptor With Potential for Thermally Activated Delayed Fluorescence Emitters
Source: Front Chem. 2020 Sep 30;8:563411. doi: 10.3389/fchem.2020.563411 (PMC7555999; doi:10.3389/fchem.2020.563411)
Supplement: Supplementary file 1 [file Data_Sheet_1.PDF]

Electronic Supporting Information (ESI)

OBO-Fused Benzo[fg]tetracene as Acceptor with Potential for Thermally Activated Delayed Fluorescence Emitters

Zhen Zhang,[1] Shiv Kumar,[2] Sergey Bagnich,[3] Eduard Spuling,[1, 2] Fabian Hundemer,[1] Martin Nieger,[4] Zahid Hassan,[1] Anna Köhler, [3]* Eli Zysman-Colman, [2]* Stefan Bräse[1, 5]*

[1] Institute of Organic Chemistry (IOC), Karlsruhe Institute of Technology (KIT), Fritz-Haber-Weg 6, 76131, Karlsruhe, Germany. E-mail: braese@kit.edu

[2] Organic Semiconductor Centre, EaStCHEM School of Chemistry, University of St Andrews, St Andrews, Fife, KY16 9ST, UK. E-mail: eli.zysman-colman@st-andrews.ac.uk

[3] Soft Matter Optoelectronics, BIMF & BPI, University of Bayreuth, Universitätsstraße 30, 95447, Bayreuth, Germany. E-mail: anna.koehler@uni-bayreuth.de

[4] Department of Chemistry, University of Helsinki, P.O. Box 55 A.I. Virtasen aukio 1, 00014 University of Helsinki, Finland.

[5] Institute of Biological and Chemical Systems – Functional Molecular Systems (IBCS-FMS), Karlsruhe Institute of Technology (KIT), Hermann von-Helmholtz-Platz 1, D-76344 Eggenstein-Leopoldshafen, Germany

*** Correspondence:**

Prof. Dr. Stefan Bräse, Prof. Dr. Eli Zysman-Colman, Prof. Dr. Anna Köhler

General information

NMR spectra were recorded using the following devices: ^{1}H NMR: *Bruker* Avance 300 (300 MHz), *Bruker* Avance 400 (400 MHz), *Bruker* Avance DRX 500 (500 MHz); ^{13}C NMR: *Bruker* AM 400 (100 MHz), *Bruker* Avance DRX 500 (125 MHz). The following solvents from *Eurisotop* were used: chloroform-d_1, tetrahydrofuran-d_8. Chemical shifts δ were expressed in parts per million (ppm) and referenced to chloroform (^{1}H: δ = 7.26 ppm, ^{13}C: δ = 77.16 ppm) or tetrahydrofuran (^{1}H: δ = 1.72 and 3.58 ppm, ^{13}C: δ = 67.21 and 25.31 ppm). The signal structure is described as follows: s = singlet, d = doublet, t = triplet, q = quartet, quin = quintet, b = broad singlet, m = multiplet, dd = doublet of doublet, dt = doublet of triplet. The spectra were analyzed according to first order. All coupling constants are absolute values and expressed in Hertz (Hz). The multiplicities of the signals of ^{13}C NMR spectra were determined using DEPT (Distortionless

Enhancement by Polarisation Transfer) and are described as follows: + = primary or tertiary (positive DEPT signal), – = secondary (negative DEPT signal), Cq = quarternary carbon atoms (no DEPT signal).

The electron ionization (EI) and fast atom bombardment (FAB) methods were conducted using an instrument by Finnigan, model MAT 90 (70 eV), and 3-nitrobenzyl alcohol (3-NBA) was used with as matrix and reference for high resolution mass spectrometry. For the interpretation of the spectra, molecular peaks $[M]^+$, peaks of pseudo molecules $[M+H]^+$ and characteristic fragment peaks are indicated with their mass to charge ratio (m/z) and in case of EI their intensity in percent, relative to the base peak (100%) is given. In the case of high resolution measurements, the tolerated error is 0.0005 m/z.

The infrared spectra of solid samples were recorded on Bruker IFS 88 and measured by attenuated total reflection (ATR method). Absorption is given in wave numbers $\bar{\upsilon}$ [cm^{-1}].

Analytical thin layer chromatography (TLC) was carried out on Merck silica gel coated aluminum plates (silica gel 60, F254), detected under UV-light at 254 nm or stained with "Seebach staining solution" (mixture of molybdato phosphoric acid, cerium(IV)-sulfate tetrahydrate, sulfuric acid and water) or basic potassium permanganate solution. Solvent mixtures are understood as volume/volume. Solvents, reagents and chemicals were purchased from Sigma-Aldrich, Chempure, ABCR and Acros Organics. All solvents, reagents and chemicals were used as purchased unless stated otherwise.

The DFT calculations, including geometry optimization of the emitters, were performed by the Gaussian 09 Revision D.01 software[1] in the gas phase at the Density Functional Theory (DFT) level using the PBE0 functional[2] and the 6-31G(d,p) basis set[3] starting with the molecular geometry obtained from single crystal X-ray diffraction analysis. Excited singlet and triplet states were calculated by performing time dependent DFT (TD-DFT) calculations within the Tamm-Dancoff approximation[4] using the same functional and basis set.

Experimental part

Synthetic procedures and analytical data

10-(3,5-dichlorophenyl)-9,9-dimethyl-9,10-dihydroacridine (1):

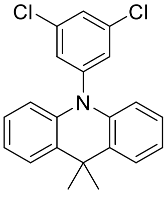

A 20 mL sealed vial was charged with 9,9-dimethyl-9,10-dihydroacridine (105 mg, 502 μmol, 1.00 equiv.), 1-bromo-3,5-dichlorobenzene (135 mg, 610 μmol, 1.21 equiv.), palladium(II) acetate (6.0 mg, 26.7 μmol, 0.05 equiv.), 4,5-bis(diphenylphosphino)-9,9-dimethylxanthene (Xantphos, 14.5 mg, 25.1 μmol, 0.05 equiv.) and sodium *tert*-butoxide (72.0 mg, 749 μmol, 1.49 equiv.). It was evacuated and flushed with argon three times. Through the septum 8 mL toluene was added, then it was heated to 100 °C and stirred for 12 h. The reaction mixture was diluted with 30 mL of ethyl acetate and washed with brine (20 mL). The organic layer was dried over $MgSO_4$ and the solvent was removed under reduced pressure. The obtained crude product was purified *via* flash-chromatography on silica gel using cyclohexane to yield the product as a white solid (113 mg, 319 μmol, 64%).

R_f = 0.65 (cyclohexane/ethyl acetate = 50:1). – **Mp =** 129 °C. – **^{1}H NMR** (400 MHz, $CDCl_3$, ppm) δ = 7.53 (t, *J* = 1.9 Hz, 1H), 7.47 (dd, *J* = 7.6 Hz, *J* = 1.7 Hz, 2H), 7.29 (d, *J* = 1.9 Hz, 2H), 7.06–6.94 (m, 4H), 6.28 (dd, *J* = 8.1 Hz, *J* = 1.4 Hz, 2H), 1.67 (s, 6H). – **^{13}C NMR** (101 MHz, $CDCl_3$, ppm) δ = 143.5 (C_q, C_{Ar}), 140.2 (C_q, 2C_{Ar}), 137.0 (C_q, 2C_{Ar}), 130.6 (C_q, 2C_{Ar}), 130.3 (+, 2C_{Ar}H), 128.9 (+, C_{Ar}H), 126.7 (+, 2C_{Ar}H), 125.6 (+, 2C_{Ar}H), 121.4 (+, 2C_{Ar}H), 114.2 (+, 2C_{Ar}H), 36.1 (C_q, *C*C_{Ar} $(CH_3)_2$), 31.3 (+, 2CH_3). – **IR** (ATR, ṽ) = 2966, 1589, 1571, 1561, 1479, 1460, 1455, 1429, 1412, 1388, 1327, 1281, 1268, 1232, 1221, 1194, 1105, 1099, 1089, 1045, 977, 929, 873, 858, 802, 745, 696, 684, 652, 612, 520, 472, 405 cm^{-1}. – **MS** (EI), *m/z*: 354 $[M+H]^+$, 353 $[M]^+$. – **HRMS** (EI, $C_{21}H_{17}N_1{}^{35}Cl_2$) calc.: 353.0738; found: 353.0736.

10-(3,5-dichlorophenyl)-10*N*-phenoxazine (2):

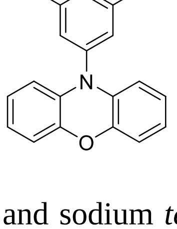

A 20 mL sealed vial was charged with 10*H*-phenoxazine (274 mg, 1.50 mmol, 1.00 equiv.), 1-bromo-3,5-dichlorobenzene (356 mg, 1.57 mmol, 1.05 equiv), palladium(II) acetate (6.7 mg, 29.9 μmol, 0.02 equiv.), 4,5-bis(diphenylphosphino)-9,9-dimethylxanthene (17.0 mg, 29.4 μmol, 0.02 equiv.) and sodium *tert*-butoxide (216 mg, 2.25 mmol, 1.50 equiv.). It was evacuated and flushed with

argon three times. Through the septum 8 mL toluene was added, then it was heated to 100 °C and stirred for 12 h. The reaction mixture was diluted with 30 mL of ethyl acetate and washed with brine (20 mL). The organic layer was dried over $MgSO_4$ and the solvent was removed under reduced pressure. The obtained crude product was purified *via* flash-chromatography on silica gel using cyclohexane to yield the product as a white solid (405 mg, 1.23 mmol, 83%).

R_f = 0.60 (cyclohexane = 1). – **Mp =** 145 °C. – **^{1}H NMR** (400 MHz, $CDCl_3$, ppm) δ = 7.49 (t, *J* = 1.9 Hz, 1H), 7.30 (d, *J* = 1.9 Hz, 2H), 6.81–6.52 (m, 6H), 6.07–5.85 (m, 2H). – **^{13}C NMR** (101 MHz, $CDCl_3$, ppm) δ = 143.8 (C_q, $2C_{Ar}$), 141.3 (C_q, C_{Ar}), 137.1 (C_q, $2C_{Ar}$), 133.3 (C_q, $2C_{Ar}$), 129.8 (+, $2C_{Ar}H$), 129.1 (+, $C_{Ar}H$), 123.4 (+, $2C_{Ar}H$), 122.1 (+, $2C_{Ar}H$), 115.8 (+, $2C_{Ar}H$), 113.3 (+, $2C_{Ar}H$). – **IR** (ATR, ṽ) = 3070, 1575, 1562, 1487, 1465, 1425, 1402, 1329, 1293, 1272, 1207, 1184, 1122, 1094, 975, 919, 873, 800, 727, 711, 693, 669, 603, 446 cm^{-1}. – **MS** (EI), *m/z*: 328 $[M+H]^+$, 327 $[M]^+$. – **HRMS** (EI, $C_{18}H_{11}O_1N_1{}^{35}Cl_2$) calc.: 327.0218; found: 327.0218.

10-(2,2''-dimethoxy-[1,1':3',1''-terphenyl]-5'-yl)-9,9-dimethyl-9,10-dihydroacridine (3)

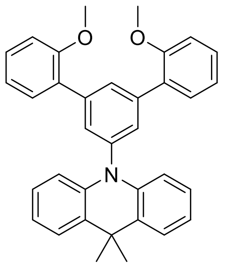

A 20 mL sealed vial was charged with 10-(3,5-dichlorophenyl)-9,9-dimethyl-9,10-dihydroacridine (88.0 mg, 248 µmol, 1.00 equiv.), (2-methoxyphenyl)boronic acid (114 mg, 750 µmol, 3.02 equiv.), palladium(II) acetate (5.6 mg, 24.9 µmol, 0.10 equiv.), 2-dicyclohexylphosphino-2′,6′-dimethoxybiphenyl (20.3 mg, 49.4 µmol, 0.20 equiv.) and potassium phosphate tribasic (527 mg, 2.48 mmol, 10.00 equiv.). It was evacuated and flushed with argon three times. Through the septum 10 mL of tetrahydrofuran and 3 mL of water were added, then it was heated to 80 °C and stirred for 12 h. The reaction mixture was diluted with 30 mL of ethyl acetate and washed with brine (20 mL). The organic layer was dried over $MgSO_4$ and the solvent was removed under reduced pressure. The obtained crude product was purified *via* flash-chromatography on silica gel using cyclohexane/ethyl acetate 20:1 to yield the product as a white solid (113 mg, 227 µmol, 91%).

R_f = 0.45 (cyclohexane/ethyl acetate = 20:1). – **Mp =** 184 °C. – **^{1}H NMR** (400 MHz, $CDCl_3$, ppm) δ = 7.86 (t, *J* = 1.6 Hz, 1H), 7.52 (d, *J* = 1.6 Hz, 2H), 7.46 (td, *J* = 7.7 Hz, *J* = 7.1 Hz, *J* = 1.6 Hz, 4H), 7.34 (td, *J* = 8.2 Hz, *J* =1.7 Hz, 2H), 7.11–6.87 (m, 8H), 6.63 (dd, *J* = 8.2 Hz, *J* =1.1 Hz, 2H), 3.83 (s, 6H), 1.71 (s, 6H). – **^{13}C NMR** (101 MHz, $CDCl_3$, ppm) δ = 156.6 (C_q, $2C_{Ar}$), 141.3 (C_q,

$2C_{Ar}$), 140.8 (C_q, $2C_{Ar}$), 140.1 (C_q, $1C_{Ar}$), 131.0 (+, $2C_{Ar}H$), 130.9 (+, $2C_{Ar}H$), 130.3 (+, $C_{Ar}H$), 130.2 (C_q, $2C_{Ar}$), 130.0 (C_q, $2C_{Ar}$), 129.1 (+, $2C_{Ar}H$), 126.4 (+, $2C_{Ar}H$), 125.0 (+, $2C_{Ar}H$), 121.0 (+, $2C_{Ar}H$), 120.5 (+, $2C_{Ar}H$), 114.6 (+, $2C_{Ar}H$), 111.4 (+, $2C_{Ar}H$), 55.8 (+, $2CH_3O$), 36.2 (C_q, $CC_{Ar}(CH_3)_2$), 31.0(+, $2CH_3$). – **IR** (ATR, ṽ) = 2922, 1587, 1496, 1472, 1466, 1446, 1412, 1323, 1273, 1249, 1218, 1179, 1160, 1122, 1045, 1024, 904, 752, 714, 662, 630, 616, 443 cm^{-1}. – **MS** (EI), *m/z*: 498 $[M+H]^+$, 497 $[M]^+$. – **HRMS** (FAB, 3-NBA, $C_{35}H_{31}O_2N_1$) calc.: 497.2355; found: 497.2355.

10-(2,2''-dimethoxy-[1,1':3',1''-terphenyl]-5'-yl)-10*N*-phenoxazine (4)

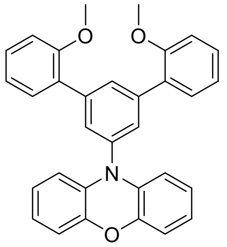

A 20 mL sealed vial was charged with 10-(3,5-dichlorophenyl)phenoxazine (164 mg, 500 µmol, 1.00 equiv.), (2-methoxyphenyl)boronic acid (227 mg, 1.49 mmol, 2.99 equiv.), palladium(II) acetate (11.0 mg, 49.0 µmol, 0.10 equiv.), 2-dicyclohexylphosphino-2′,6′-dimethoxybiphenyl (41.0 mg, 99.9 µmol, 0.20 equiv.) and potassium phosphate tribasic (1.06 g, 5.00 mmol, 10.00 equiv.). It was evacuated and flushed with argon three times. Through the septum 10 mL of tetrahydrofuran and 3 mL of water were added, then it was heated to 80 °C and stirred for 12 h. The reaction mixture was diluted with 30 mL of ethyl acetate and washed with brine (20 mL). The organic layer was dried over $MgSO_4$ and the solvent was removed under reduced pressure. The obtained crude product was purified *via* flash-chromatography on silica gel using cyclohexane/dichloromethane 15:1 to 5:1 to yield the product as a white solid (220 mg, 467 µmol, 93%).

$\mathbf{R_f}$ = 0.20 (cyclohexane/dichloromethane = 15:1). – **Mp =** 149 °C. – **^{1}H NMR** (400 MHz, $CDCl_3$, ppm) δ = 7.80 (t, *J* = 1.6 Hz, 1H), 7.54 (d, *J* = 1.5 Hz, 2H), 7.44 (dd, *J* = 7.5 Hz, *J* = 1.7 Hz, 2H), 7.35 (td, *J* = 8.2 Hz, *J* = 1.7 Hz, 2H), 7.14–6.93 (m, 4H), 6.80–6.54 (m, 6H), 6.38–6.16 (m, 2H), 3.84 (s, 6H). – **^{13}C NMR** (101 MHz, $CDCl_3$, ppm) δ = 156.5 (+, $2C_{Ar}H$), 144.2 (C_q, $2C_{Ar}$), 140.9 (C_q, $2C_{Ar}$), 137.7 (C_q, C_{Ar}), 134.6 (C_q, $2C_{Ar}$), 130.8 (+, $2C_{Ar}H$), 130.4 (+, $2C_{Ar}H$), 130.2 (+, $C_{Ar}H$), 129.7 (C_q, $2C_{Ar}$), 129.2 (+, $2C_{Ar}H$), 123.3 (+, $2C_{Ar}H$), 121.3 (+, $2C_{Ar}H$), 121.0 (+, $2C_{Ar}H$), 115.4 (+, $2C_{Ar}H$), 113.7 (+, $2C_{Ar}H$), 111.4 (+, $2C_{Ar}H$), 55.7 (C_q, $2COCH_3$). – **IR** (ATR, ṽ) = 2839, 1587, 1497, 1482, 1462, 1455, 1431, 1414, 1324, 1288, 1272, 1262, 1242, 1200, 1181, 1163, 1119, 1047,

1026, 892, 742, 718, 670, 647, 615, 602, 567, 547, 520, 489, 475, 441, 424 cm^{-1}. – **MS** (EI), *m/z*: 472 $[M+H]^+$, 471 $[M]^+$. – **HRMS** (FAB, 3-NBA, $C_{32}H_{25}O_3N_1$) calc.: 471.1834; found: 471.1833.

10-(8,9-dioxa-8a-borabenzo[fg]tetracen-2-yl)-9,9-dimethyl-9,10-dihydroacridine (DMAC-OBO)

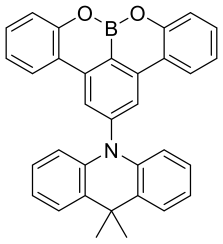

To a solution of compound 10-(2,2''-dimethoxy-[1,1':3',1''-terphenyl]-5'-yl)-9,9-dimethyl-9,10-dihydroacridine (1.00 g, 2.01 mmol, 1.00 equiv) in anhydrous dichlorobenzene (30 mL) was added tribromoborane (1.00 g, 4.00 mL, 4.00 mmol, 1.00 M in heptane, 1.99 equiv.) under argon. Then the mixture was heated to 150 °C and stirred at this temperature for 12 h. After quenching with methanol, the reaction mixture was concentrated under reduced pressure. The obtained crude product was purified *via* flash-chromatography on silica gel using cyclohexane/dichloromethane 3:1 to yield the product as a white solid (535 mg, 1.12 mmol, 56%).

$\mathbf{R}_f$ = 0.56 (cyclohexane/dichloromethane = 3:1). – **Mp =** 310 °C. – **^{1}H NMR** ((500 MHz, THF-d_8, ppm) δ = 8.28 (dd, *J* = 8.1 Hz, *J* =1.2 Hz, 2H), 8.24 (s, 2H), 7.54–7.49 (m, 2H), 7.49–7.43 (m, 4H), 7.23 (ddd, *J* = 8.2 Hz, *J* =5.6 Hz, *J* =2.7 Hz, 2H), 6.94–6.85 (m, 4H), 6.36–6.29 (m, 2H), 1.75 (s, 6H). – **^{13}C NMR** (126 MHz, THF-d_8, ppm) δ = 152.9 (C_q, $2C_{Ar}$), 148.0 (C_q, C_{Ar}), 143.6 (C_q, $2C_{Ar}$), 141.5 (C_q, $2C_{Ar}$), 130.7 (+, $2C_{Ar}H$), 130.6 (C_q, $2C_{Ar}$), 127.0 (+, $2C_{Ar}H$), 126.0 (+, $2C_{Ar}H$), 125.5 (+, $2C_{Ar}H$), 123.8 (+, $2C_{Ar}H$), 123.7 (+, $2C_{Ar}H$), 123.6 (C_q, $2C_{Ar}$), 121.3 (+, $2C_{Ar}H$), 120.9 (+, $2C_{Ar}H$), 114.9 (+, $2C_{Ar}H$), 36.6 (C_q, $\mathit{C}C_{Ar}(CH_3)_2$), 31.9 (+, $2CH_3$), 1C is missing (1C, C–B)[5]. –**^{11}B NMR** (160 MHz, THF-d_8, ppm) δ = 27.81. – **IR** (ATR): ṽ = 2968, 1611, 1589, 1584, 1568, 1555, 1500, 1480, 1460, 1438, 1418, 1371, 1334, 1281, 1258, 1218, 1205, 1174, 1157, 1146, 1135, 1119, 1085, 1044, 1035, 972, 941, 933, 916, 874, 856, 807, 779, 764, 744, 737, 704, 674, 632, 619, 611, 572, 533, 497, 453, 428 cm^{-1}. – **MS** (EI), *m/z*: 478 $[M+H]^+$, 477 $[M]^+$. – **HRMS** (EI, $C_{33}H_{24}N_1O_2{}^{11}B_1$) calc.: 477.1900; found: 477.1898.

10-(8,9-dioxa-8a-borabenzo[fg]tetracen-2-yl)-10*N*-phenoxazine (PXZ-OBO)

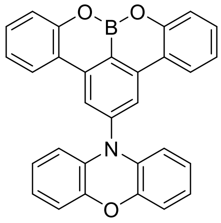

To a solution of compound 10-(2,2''-dimethoxy-[1,1':3',1''-terphenyl]-5'-yl)-10H-phenoxazine (2.85 g, 6.04 mmol, 1.00 equiv.) in anhydrous dichlorobenzene (50 mL) was added tribromoborane (3.03 g, 12.1 mL, 12.10 mmol, 1.00 M in heptane, 2.00 equiv.) under argon. Then the mixture was heated to 150 °C and stirred at this temperature for 12 h. After quenching with methanol, the reaction mixture was concentrated under reduced pressure. The obtained crude product was purified *via* flash-chromatography on silica gel using cyclohexane/dichloromethane 3:1 to 2:1 to yield the product as a yellow solid (1.50 g, 3.32 mmol, 55%).

R_f = 0.53 (cyclohexane/dichloromethane = 3:1). – **Mp =** 337 °C. – **^{1}H NMR** (500 MHz, THF-d_8, ppm) δ = 8.30 (m, 4H), 7.59–7.40 (m, 4H), 7.26 (ddd, *J* = 8.2 Hz, *J* = 6.6 Hz, *J* =1.8 Hz, 2H), 6.71 (dd, *J* = 7.9 Hz, *J* = 1.6 Hz, 2H), 6.65 (td, *J* = 7.7 Hz, 1.5 Hz, 2H), 6.57 (td, *J* = 7.7 Hz, 1.6 Hz, 2H), 6.04 (dd, *J* = 8.0 Hz, *J* = 1.5 Hz, 2H). – **^{13}C NMR** (126 MHz, THF-d_8, ppm) δ = 153.2 (C_q, 2C_{Ar}), 146.1 (C_q, 2C_{Ar}), 145.1 (C_q, 2C_{Ar}), 144.2 (C_q, 2C_{Ar}), 135.4 (C_q, 2C_{Ar}), 131.2 (+, 2C_{Ar}H), 125.8 (+, 2C_{Ar}H), 124.4 (+, 2C_{Ar}H), 124.2 (+, 2C_{Ar}H), 123.9 (C_q, C_{Ar}), 123.6 (+, 2C_{Ar}H), 122.5 (+, 2C_{Ar}H), 121.3 (+, 2C_{Ar}H), 116.4 (+, 2C_{Ar}H), 114.6 (+, 2C_{Ar}H), 1C is missing (1C, C–B)[5]. – **^{11}B NMR** (160 MHz, THF-d_8, ppm) δ = 26.5 ppm.– **IR** (ATR): ṽ = 3061, 1604, 1581, 1555, 1487, 1463, 1418, 1374, 1339, 1315, 1292, 1269, 1238, 1220, 1207, 1166, 1156, 1143, 1118, 1085, 1044, 1034, 938, 915, 874, 857, 781, 754, 735, 711, 681, 662, 625, 612, 602, 534, 497, 455, 448 cm^{-1}. – **MS** (FAB, 3-NBA), *m/z*: 452 $[M+H]^+$, 451 $[M]^+$. – **HRMS** (FAB, 3-NBA, $C_{30}H_{18}N_1O_3{}^{11}B_1$) calc.: 451.1380; found: 451.1381.

10-(3-chloro-4-methoxyphenyl)-9,9-dimethyl-9,10-dihydroacridine (5)

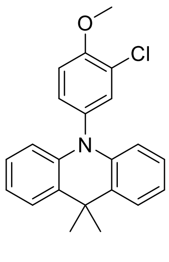

A 20 mL sealed vial was charged with 9,9-dimethyl-10*H*-acridine (157 mg, 750 µmol, 1.00 equiv.), 4-bromo-2-chloro-1-methoxybenzene (174 mg, 787 µmol, 1.05 equiv.), palladium(II) acetate (3.4 mg, 15.1 µmol, 0.02 equiv.), 4,5-bis(diphenylphosphino)-9,9-dimethylxanthene (8.7 mg, 15.0 µmol, 0.02 equiv.) and sodium *tert*-butoxide (108 mg, 1.12 mmol, 1.50 equiv.). It was evacuated and flushed with argon three times. Through the septum 8 mL toluene was added, then it was heated to 100 °C and stirred for 12 h. The reaction mixture was diluted with 30 mL of ethyl acetate and

washed with brine (20 mL). The organic layer was dried over $MgSO_4$ and the solvent was removed under reduced pressure. The obtained crude product was purified *via* flash-chromatography on silica gel using cyclohexane/ethyl acetate 20:1to yield the product as a white solid (260 mg, 743 µmol, 99%).

R$_f$ = 0.40 (cyclohexane/ethyl acetate = 20:1). – **Mp =** 186 °C. – **^{1}H NMR** (400 MHz, $CDCl_3$, ppm) δ = 7.46 (dd, *J* = 7.6 Hz, *J* = 1.6 Hz, 2H), 7.39 (d, *J* = 2.4 Hz, 1H), 7.24 (dd, *J* = 8.6 Hz, *J* = 2.4 Hz, 1H), 7.16 (d, *J* = 8.6 Hz, 1H), 7.02–6.92 (m, 4H), 6.31 (dd, *J* = 8.1 Hz, *J* = 1.2 Hz, 2H), 4.02 (s, 3H), 1.69 (s, 6H). – **^{13}C NMR** (101 MHz, $CDCl_3$, ppm) δ = 155.0 (C_q, C_{Ar}), 141.0 (C_q, $2C_{Ar}$), 134.2 (C_q, C_{Ar}), 133.2 (+, $C_{Ar}H$), 130.8 (+, $C_{Ar}H$), 130.2 (C_q, $2C_{Ar}$), 126.5 (+, $2C_{Ar}H$), 125.4 (+, $2C_{Ar}H$), 124.2 (C_q, C_{Ar}), 120.9 (+, $2C_{Ar}H$), 114.1 (+, $2C_{Ar}H$), 113.6 (+, $C_{Ar}H$), 56.5 (+, CH_3O), 36.1 (C_q, $\mathit{C}C_{Ar}$ $(CH_3)_2$), 31.4 (+, $2CH_3$). – **IR** (ATR, ṽ) = 2962, 1589, 1494, 1475, 1438, 1395, 1387, 1326, 1285, 1275, 1262, 1239, 1183, 1164, 1145, 1129, 1111, 1089, 1058, 1047, 1021, 959, 929, 826, 800, 759, 749, 725, 698, 646, 612, 581, 569, 520, 429 cm^{-1}. – **MS** (EI), *m/z*: 350 $[M+H]^+$, 349 $[M]^+$. – **HRMS** (EI, $C_{22}H_{20}O_1N_1{}^{35}Cl_1$) calc.: 349.1233; found: 349.1231.

10-(3-chloro-4-methoxyphenyl)-10*N*-phenoxazine (6)

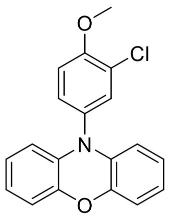

A 20 mL sealed vial was charged with 10*H*-phenoxazine (137 mg, 750 µmol, 1.00 equiv.), 4-bromo-2-chloro-1-methoxybenzene (174 mg, 787 µmol, 1.05 equiv.), palladium(II) acetate (3.3 mg, 14.7 µmol, 0.02 equiv.), 4,5-bis(diphenylphosphino)-9,9-dimethylxanthene (8.6 mg, 15.0 µmol, 0.02 equiv.) and sodium *tert*-butoxide (108 mg, 1.12 mmol, 1.50 equiv.). It was evacuated and flushed with argon three times. Through the septum 8 mL toluene was added, then it was heated to 100 °C and stirred for 12 h. The reaction mixture was diluted with 30 mL of ethyl acetate and washed with brine (20 mL). The organic layer was dried over $MgSO_4$ and the solvent was removed under reduced pressure. The obtained crude product was purified *via* flash-chromatography on silica gel using cyclohexane/dichloromethane 2:1 to 1:1 to yield the product as a white solid (217 mg, 670 µmol, 89%).

R$_f$ = 0.35 (cyclohexane/dichloromethane = 2:1). – **Mp =** 210 °C. – **^{1}H NMR** (400 MHz, $CDCl_3$, ppm) δ = 7.38 (d, *J* = 2.5 Hz, 1H), 7.23 (dd, *J* = 8.6 Hz, *J* = 2.5 Hz, 1H), 7.12 (d, *J* = 8.7 Hz, 1H),

6.77–6.53 (m, 6H), 5.93 (dd, J = 7.6 Hz, J = 1.6 Hz, 2H), 3.98 (s, 3H). – **^{13}C NMR** (101 MHz, $CDCl_3$, ppm) δ = 155.3 (C_q, C_{Ar}), 144.1 (C_q, 2C_{Ar}), 134.5 (C_q, 2C_{Ar}), 132.8 (+, C_{Ar}H), 132.0 (C_q, C_{Ar}), 130.6 (+, C_{Ar}H), 124.6 (C_q, C_{Ar}), 123.5 (+, 2C_{Ar}H), 121.7 (+, 2C_{Ar}H), 115.7 (+, 2C_{Ar}H), 113.9 (+, C_{Ar}H), 113.4 (+, 2C_{Ar}H), 56.6 (+, CH_3O). – **IR** (ATR, ṽ) = 3024, 2970, 1626, 1591, 1570, 1507, 1486, 1460, 1435, 1394, 1337, 1290, 1271, 1262, 1245, 1204, 1184, 1150, 1142, 1116, 1094, 1058, 1043, 1018, 958, 929, 885, 857, 837, 815, 744, 721, 707, 683, 633, 609, 589, 557, 537, 456, 416, 402 cm^{-1}. – **MS** (EI), *m/z*: 324 $[M+H]^+$, 323 $[M]^+$. – **HRMS** (EI, $C_{19}H_{14}O_2N_1{}^{35}Cl_1$) calc.: 323.0713; found: 323.0712.

<u>10-(2'',6-dimethoxy-[1,1':3',1''-terphenyl]-3-yl)-9,9-dimethyl-9,10-dihydroacridine (7)</u>

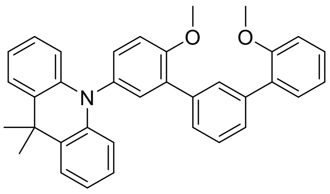

A 100 mL round flask was charged with 10-(3-chloro-4-methoxyphenyl)-9,9-dimethyl-9,10-dihydroacridine (1.46 g, 4.17 mmol, 1.00 equiv.), 2-(2'-methoxy-[1,1'-biphenyl]-3-yl)-4,4,5,5-tetramethyl-1,3,2-dioxaborolane (1.43 g, 4.61 mmol, 1.10 equiv.), palladium(II) acetate (46.4 mg, 207 μmol, 0.05 equiv.), 2-dicyclohexylphosphino-2′,6′-dimethoxybiphenyl (70.9 mg, 173 μmol, 0.04 equiv.) and potassium phosphate tribasic (2.65 g, 12.5 mmol, 2.99 equiv.). It was evacuated and flushed with argon three times. Through the septum 50 mL toluene was added, then it was heated to 100 °C and stirred for 12 h. The reaction mixture was diluted with 50 mL of ethyl acetate and washed with brine (2 × 50 mL). The organic layer was dried over $MgSO_4$ and the solvent was removed under reduced pressure. The obtained crude product was purified *via* flash-chromatography on silica gel using cyclohexane/dichloromethane 5:1 to 2:1 to yield the product as a white solid (1.91 g, 3.84 mmol, 92%).

R_f = 0.35 (cyclohexane/dichloromethane = 5:1). – **Mp =** 77 °C. – **^{1}H NMR** (400 MHz, $CDCl_3$, ppm) δ = 7.71 (t, J = 1.5 Hz, 1H), 7.55 (dt, J = 7.5 Hz, J = 1.5 Hz, 1H), 7.51 (dt, J = 7.7 Hz, J = 1.5 Hz, 1H), 7.44 (dd, J = 7.6 Hz, J = 1.6 Hz, 3H), 7.38–7.33 (m, 2H), 7.33–7.27 (m, 1H), 7.26–7.17 (m, 2H), 7.04–6.95 (m, 4H), 6.91 (td, J = 7.4 Hz, J = 1.4 Hz, 2H), 6.42 (dd, J = 8.2, J = 1.1 Hz, 2H), 3.93 (s, 3H), 3.79 (s, 3H), 1.68 (s, 6H). – **^{13}C NMR** (101 MHz, $CDCl_3$, ppm) δ = 156.6 (C_q, C_{Ar}), 156.2 (C_q, C_{Ar}), 141.4 (C_q, 2C_{Ar}), 138.5 (C_q, C_{Ar}), 137.4 (C_q, C_{Ar}), 133.9 (C_q, C_{Ar}), 133.7 (+, C_{Ar}H), 133.3 (C_q, C_{Ar}), 131.1 (+, C_{Ar}H), 131.0 (+, C_{Ar}H), 130.8 (C_q, 2C_{Ar}), 130.7 (+, C_{Ar}H), 130.1 (+, C_{Ar}H), 128.7 (+, C_{Ar}H), 128.2 (+, 2C_{Ar}H), 127.8 (+, C_{Ar}H), 126.5 (+, 2C_{Ar}H), 125.2 (+,

2C$_{Ar}$H), 120.9 (+, C$_{Ar}$H), 120.5 (+, 2C$_{Ar}$H), 114.2 (+, 2C$_{Ar}$H), 113.1 (+, C$_{Ar}$H), 111.3 (+, C$_{Ar}$H), 56.0 (+, CH_3O), 55.7 (+, CH_3O), 36.1 (C$_q$, *C*C$_{Ar}$ $(CH_3)_2$), 31.3 (+, 2CH_3). – **IR** (ATR, ṽ) = 2924, 1589, 1499, 1472, 1463, 1443, 1400, 1383, 1324, 1264, 1239, 1179, 1163, 1132, 1122, 1088, 1047, 1026, 928, 902, 798, 744, 704, 663, 618, 602, 579, 431 cm^{-1}. – **MS** (FAB, 3-NBA), *m/z*: 499 $[M+H]^+$, 498 $[M]^+$. – **HRMS** (FAB, 3-NBA, $C_{35}H_{32}O_2N_1$) calc.: 498.2433 $[M+H]^+$; found: 498.2431 $[M+H]^+$.

10-(2'',6-dimethoxy-[1,1':3',1''-terphenyl]-3-yl)-10*N*-phenoxazine (8)

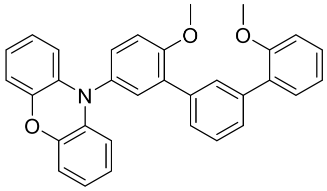

A 100 mL round flask was charged with 10-(3-chloro-4-methoxyphenyl)-10*N*-phenoxazine (1.36 g, 4.20 mmol, 1.00 equiv.), 2-(2'-methoxy-[1,1'-biphenyl]-3-yl)-4,4,5,5-tetramethyl-1,3,2-dioxa-borolane (1.43 g, 4.61 mmol, 1.10 equiv.), palladium(II) acetate (47.0 mg, 209 µmol, 0.05 equiv.), 2-dicyclohexylphosphino-2′,6′-dimethoxybiphenyl (172 mg, 419 µmol, 0.10 equiv.) and potassium phosphate tribasic (2.67 g, 12.6 mmol, 2.99 equiv). It was evacuated and flushed with argon three times. Through the septum 50 mL toluene was added, then it was heated to 100 °C and stirred for 12 h. The reaction mixture was diluted with 50 mL of ethyl acetate and washed with brine (2 × 50 mL). The organic layer was dried over MgSO4 and the solvent was removed under reduced pressure. The obtained crude product was purified *via* flash-chromatography on silica gel using cyclohexane/dichloromethane 5:1 to 2:1 to yield the product as a white solid (1.46 g, 3.10 mmol, 74%).

R*f* = 0.35 (cyclohexane/dichloromethane = 5:1). – **Mp =** 172 °C. – **^{1}H NMR** (400 MHz, $CDCl_3$, ppm) δ = 7.70 (t, *J* = 1.6 Hz, 1H), 7.58–7.50 (m, 2H), 7.45 (t, *J* = 7.6 Hz, 1H), 7.40–7.24 (m, 4H), 7.17 (d, *J* = 8.6 Hz, 1H), 7.08–6.96 (m, 2H), 6.75–6.52 (m, 6H), 6.04 (d, *J* = 6.2 Hz, 2H), 3.92 (s, 3H), 3.82 (s, 3H). – **^{13}C NMR** (101 MHz, $CDCl_3$, ppm) δ = 156.6 (C$_{q,}$ C$_{Ar}$OCH_3), 156.4 (C$_{q,}$ C$_{Ar}$OCH_3), 144.1 (C$_{q,}$ C$_{Ar}$), 138.5 (C$_{q,}$ 2C$_{Ar}$), 137.2 (C$_{q,}$ C$_{Ar}$), 134.8 (C$_{q,}$ C$_{Ar}$), 133.5 (C$_{q,}$ C$_{Ar}$), 133.1 (+, C$_{Ar}$H), 131.6 (C$_{q,}$ C$_{Ar}$), 131.1 (+, C$_{Ar}$H), 130.7 (C$_{q,}$ 2C$_{Ar}$), 130.6 (+, 2C$_{Ar}$H), 128.9 (+, C$_{Ar}$H), 128.8 (+, C$_{Ar}$H), 128.1 (+, C$_{Ar}$H), 127.8 (+, C$_{Ar}$H), 123.4 (+, C$_{Ar}$H), 121.2 (+, C$_{Ar}$H), 120.9 (+, 2C$_{Ar}$H), 115.4 (+, C$_{Ar}$H), 113.4 (+, 2C$_{Ar}$H), 113.3 (+, C$_{Ar}$H), 111.3 (+, 2C$_{Ar}$H), 56.0 (+, CH_3O), 55.7 (+, CH_3O). – **IR** (ATR, ṽ) = 2925, 1592, 1482, 1460, 1439, 1398, 1384, 1329, 1290, 1269, 1244, 1221, 1200, 1179, 1162, 1132, 1118, 1094, 1051, 1043, 1027, 976, 955, 928, 914, 905, 891,

858, 799, 738, 704, 656, 640, 615, 579, 538, 524, 509, 499, 490, 456, 442, 432 cm^{-1}. – **MS** (FAB, 3-NBA), *m/z*: 472 $[M+H]^+$, 471 $[M]^+$. – **HRMS** (FAB, 3-NBA, $C_{32}H_{25}O_3N_1$) calc.: 471.1834; found: 471.1834.

10,10'-(6,6''-dimethoxy-[1,1':3',1''-terphenyl]-3,3''-diyl)bis(9,9-dimethyl-9,10 dihydroacridine) (9)

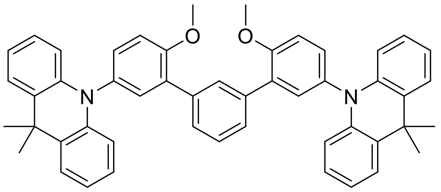

A 20 mL vial was charged with 10-(3-chloro-4-methoxyphenyl)-9,9-dimethyl-9,10-dihydroacridine (160 mg, 457 μmol, 1.00 equiv.), 1,3-bis(4,4,5,5-tetramethyl-1,3,2-dioxaborolan-2-yl)benzene (73.6 mg, 223 μmol, 0.98 equiv.), palladium(II) acetate (2.5 mg, 11.1 μmol, 0.05 equiv.), 2-dicyclohexylphosphino-2′,6′-dimethoxybiphenyl (9.1 mg, 22.2 μmol, 0.10 equiv.) and potassium phosphate tribasic (284 mg, 1.34 mmol, 5.85 equiv.). It was evacuated and flushed with argon three times. Through the septum 5 mL toluene was added, then it was heated to 100 °C and stirred for 12 h. The reaction mixture was diluted with 20 mL of ethyl acetate and washed with brine (2 × 30 mL). The organic layer was dried over $MgSO_4$ and the solvent was removed under reduced pressure. The obtained crude product was purified *via* flash-chromatography on silica gel using cyclohexane/dichloromethane 3:1 to 2:1 to yield the product as a white solid (155 mg, 220 μmol, 96%).

$\mathbf{R}_{\boldsymbol{f}}$ = 0.30 (cyclohexane/dichloromethane = 2:1). – **Mp =** 254 °C. – **^{1}H NMR** (400 MHz, $CDCl_3$, ppm) δ = 7.76 (t, *J* = 1.5 Hz, 1H), 7.54 (dd, *J* = 7.3 Hz, *J* = 1.6 Hz, 2H), 7.45 (dd, *J* = 7.7 Hz, *J* = 1.5 Hz, 5H), 7.34 (d, *J* = 2.5 Hz, 2H), 7.30–7.24 (m, 2H), 7.18 (d, *J* = 8.6 Hz, 2H), 7.05–6.83 (m, 8H), 6.42 (dd, *J* = 8.1 Hz, *J* = 1.2 Hz, 4H), 3.91 (s, 6H), 1.68 (s, 12H). – **^{13}C NMR** (101 MHz, $CDCl_3$, ppm) δ = 156.2 (C_q, $2C_{Ar}$), 141.4 (C_q, $4C_{Ar}$), 137.5 (C_q, $2C_{Ar}$), 133.9 (C_q, $2C_{Ar}$), 133.7 (+, $2C_{Ar}H$), 133.2 (C_q, $2C_{Ar}$), 131.1 (+, $2C_{Ar}H$), 130.7 (+, $C_{Ar}H$), 130.1 (C_q, $4C_{Ar}$), 128.5 (+, $2C_{Ar}H$), 127.9 (+, $C_{Ar}H$), 126.5 (+, $4C_{Ar}H$), 125.2 (+, $4C_{Ar}H$), 120.5 (+, $4C_{Ar}H$), 114.2 (+, $4C_{Ar}H$), 113.1 (+, $2C_{Ar}H$), 56.0 (+, $2CH_3O$), 36.1 (Cq, 2*C*CAr $(CH_3)_2$), 31.3 (+, $4CH_3$). – **IR** (ATR, ṽ) = 2919, 2847, 1589, 1496, 1479, 1462, 1441, 1401, 1332, 1282, 1265, 1252, 1241, 1232, 1220, 1180, 1128, 1045, 1026, 803, 748, 741, 704, 602, 507 cm^{-1}. – **MS** (FAB, 3-NBA), *m/z*: 706 $[M+H]^+$, 705 $[M]^+$. – **HRMS** (FAB, 3-NBA, $C_{50}H_{45}O_2N_2$) calc.: 705.3481 $[M+H]^+$; found: 705.3479 $[M+H]^+$.

<u>10,10'-(6,6''-dimethoxy-[1,1':3',1''-terphenyl]-3,3''-diyl)bis(10*N*-phenoxazine) (10)</u>

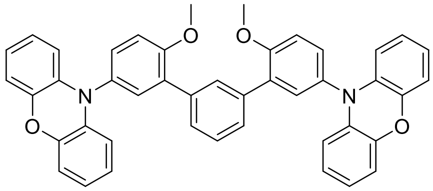

A 50 mL vial was charged with 10-(3-chloro-4-methoxyphenyl)-10*N*-phenoxazine (906 mg, 2.80 mmol, 1.00 equiv.), 1,3-bis(4,4,5,5-tetramethyl-1,3,2-dioxaborolan-2-yl)benzene (440 mg, 1.33 mmol, 0.95 equiv.), palladium(II) acetate (14.9 mg, 66.4 µmol, 0.05 equiv), 2-dicyclohexylphosphino-2′,6′-dimethoxybiphenyl (54.7 mg, 133 µmol, 0.10 equiv.) and potassium phosphate tribasic (1.70 g, 8.01 mmol, 5.72 equiv.). It was evacuated and flushed with argon three times. Through the septum 20 mL toluene was added, then it was heated to 100 °C and stirred for 12 h. The reaction mixture was diluted with 30 mL of ethyl acetate and washed with brine (2 × 30 mL). The organic layer was dried over $MgSO_4$ and the solvent was removed under reduced pressure. The obtained crude product was purified *via* flash-chromatography on silica gel using cyclohexane/dichloromethane 10:3 to 2:1 to yield the product as a white solid (600 mg, 919 µmol, 66%).

R_f = 0.40 (cyclohexane/dichloromethane = 10:3). – **Mp =** 302 °C. – **^{1}H NMR** (400 MHz, $CDCl_3$, ppm) δ = 7.69 (t, *J* = 1.6 Hz, 1H), 7.56–7.53 (m, 2H), 7.43–7.47 (m, 1H), 7.34 (d, *J* = 2.6 Hz, 2H), 7.28 (dd, *J* = 8.6 Hz, *J* = 2.6 Hz, 2H), 7.16 (d, *J* = 8.6 Hz, 2H), 6.68–6.57 (m, 12H), 6.03 (d, *J* = 7.3 Hz, 4H), 3.89 (s, 6H). – **^{13}C NMR** (101 MHz, $CDCl_3$, ppm) δ = 156.4 (C_q, $2C_{Ar}OCH_3$), 144.1 (C_q, $2C_{Ar}$), 137.4 (C_q, $4C_{Ar}$), 134.8 (C_q, $2C_{Ar}$), 133.4 (C_q, $4C_{Ar}$), 133.1 (+, $C_{Ar}H$), 131.6 (C_q, $2C_{Ar}$), 130.8 (+, $C_{Ar}H$), 130.5 (+, $2C_{Ar}H$), 128.6 (+, $4C_{Ar}H$), 128.0 (+, $2C_{Ar}H$), 123.4 (+, $4C_{Ar}H$), 121.3 (+, $2C_{Ar}H$), 115.5 (+, $2C_{Ar}H$), 113.4 (+, $4C_{Ar}H$), 113.4 (+, $4C_{Ar}H$), 56.0 (+, $2CH_3O$). – **IR** (ATR, ṽ) = 3033, 1589, 1482, 1462, 1442, 1400, 1330, 1292, 1269, 1245, 1237, 1204, 1181, 1154, 1129, 1116, 1098, 1086, 1068, 1041, 1030, 928, 902, 887, 861, 816, 796, 739, 713, 697, 686, 667, 656, 636, 578, 462, 439, 404 cm^{-1}. – **MS** (FAB, 3-NBA), *m/z*: 653 $[M+H]^+$, 652 $[M]^+$. – **HRMS** (FAB, 3-NBA, $C_{44}H_{32}O_4N_2$) calc.: 652.2362; found: 652.2360.

10-(8,9-dioxa-8a-borabenzo[fg]tetracen-12-yl)-9,9-dimethyl-9,10-dihydroacridine (5DMAC-OBO)

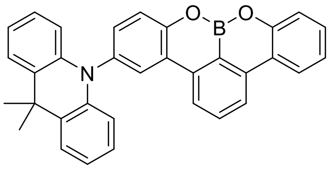

To a solution of compound 10-(2'',6-dimethoxy-[1,1':3',1''-terphenyl]-3-yl)-9,9-dimethyl-9,10-dihydroacridine (1.70 g, 3.42 mmol, 1.00 equiv.) in anhydrous dichlorobenzene (45 mL) was added tribromoborane (1.71 g, 6.83 mL, 6.83 mmol, 1.00 M in heptane, 2.00 equiv.) under argon. Then the mixture was heated to 150 °C and stirred at this temperature for 12 h. After quenching with methanol, the reaction mixture was concentrated under reduced pressure. The obtained crude product was purified *via* flash-chromatography on silica gel using cyclohexane/dichloromethane 3:1 to 2:1to yield the product as a white solid (360 mg, 754 µmol, 22%).

R$_f$ = 0.54 (cyclohexane/dichloromethane = 3:1). – **Mp =** 278 °C. – **^{1}H NMR** (500 MHz, THF-d_8, ppm) δ = 8.32–8.27 (m, 2H), 8.24 (dd, *J* = 14.4 Hz, *J* = 8.0 Hz, 2H), 7.90 (t, *J* = 7.9 Hz, 1H), 7.70 (d, *J* = 8.5 Hz, 1H), 7.51–7.38 (m, 5H), 7.29 (ddd, *J* = 8.2 Hz, *J* = 6.3 Hz, *J* = 2.0 Hz, 1H), 6.89 (m, 4H), 6.35 (dd, *J* = 8.1 Hz, *J* = 1.4 Hz, 2H), 1.70 (s, 6H). – **^{13}C NMR** (126 MHz, THF-d_8, ppm) δ = 153.2 (C_q, C_{Ar}), 152.7 (C_q, C_{Ar}), 142.3 (C_q, C_{Ar}), 140.6 (C_q, C_{Ar}), 140.0 (C_q, C_{Ar}), 137.5 (C_q, C_{Ar}), 135.1 (+, C_{Ar}H), 133.4 (+, C_{Ar}H), 131.0 (C_q, C_{Ar}), 130.6 (+, C_{Ar}H), 128.4 (+, C_{Ar}H), 127.3 (+, C_{Ar}H), 127.1 (C_q, C_{Ar}), 126.1 (+, C_{Ar}H), 125.3 (+, C_{Ar}H), 124.4 (C_q, C_{Ar}), 124.1 (+, C_{Ar}H), 123.8 (+, C_{Ar}H), 121.5 (+, C_{Ar}H), 121.4 (+, C_{Ar}H), 121.3 (+, C_{Ar}H), 115.1 (+, C_{Ar}H), 36.9 (Cq, *C*CAr $(CH_3)_2$), 31.9 (+, CH_3), 1C is missing (1C, C–B) [5]. – **IR** (ATR): ṽ = 2955, 2948, 1602, 1587, 1560, 1486, 1473, 1446, 1375, 1329, 1288, 1269, 1241, 1210, 1177, 1162, 1130, 1118, 1103, 1089, 1074, 1061, 1044, 1024, 929, 877, 861, 837, 827, 810, 754, 739, 701, 666, 630, 616, 567, 543, 531, 509, 483, 425 cm^{-1}. – **MS** (FAB, 3-NBA), *m/z*: 478 [M+H]$^+$, 477 [M]$^+$. – **HRMS** ($C_{33}H_{24}N_1O_2{}^{11}B_1$) calc.: 477.1900; found: 477.1901.

10-(8,9-dioxa-8a-borabenzo[fg]tetracen-12-yl)-10*N*-phenoxazine (5PXZ-OBO)

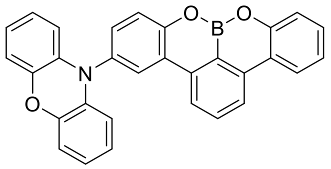

To a solution of compound 10-(2'',6-dimethoxy-[1,1':3',1''-terphenyl]-3-yl)-10*N*-phenoxazine (930 mg, 1.97 mmol, 1.00 equiv.) in anhydrous dichlorobenzene (30 mL) was added tribromoborane (988

mg, 3.94 mL, 3.94 mmol,1.00 M in heptane, 2.00 equiv) under argon. Then the mixture was heated to 150 °C and stirred at this temperature for 12 h. After quenching with methanol, the reaction mixture was concentrated under reduced pressure. The obtained crude product was purified *via* flash-chromatography on silica gel using cyclohexane/dichloromethane 2:1 to 1:1 to yield the product as a yellow solid (205 mg, 454 µmol, 23%).

R_f = 0.37 (cyclohexane/dichloromethane = 2:1). – **Mp =** 300 °C. – **^{1}H NMR** (300 MHz, THF-d_8, ppm) δ = 8.36 (d, *J* = 2.5 Hz, 1H), 8.34–8.23 (m, 3H), 7.93 (t, *J* = 7.9 Hz, 1H), 7.68 (d, *J* = 8.6 Hz, 1H), 7.54–7.40 (m, 3H), 7.29 (ddd, *J* = 8.3 Hz, *J* = 6.3 Hz, *J* = 2.2 Hz, 1H), 6.73–6.51 (m, 6H), 6.01 (dd, *J* = 7.5 Hz, *J* = 1.9 Hz, 2H). – **^{13}C NMR** (101 MHz, THF-d_8, ppm) δ = 152.8 (C_q, C_{Ar}), 152.5 (C_q, C_{Ar}), 144.8 (C_q, 2C_{Ar}), 140.3 (C_q, C_{Ar}), 139.5 (C_q, C_{Ar}), 135.4 (C_q, 2C_{Ar}), 134.8 (C_q, C_{Ar}), 134.7 (C_q, C_{Ar}H), 132.6 (C_q, C_{Ar}H), 130.3 (C_q, C_{Ar}H), 127.7 (C_q, C_{Ar}H), 127.0 (C_q, C_{Ar}), 124.9 (C_q, C_{Ar}H), 124.1 (C_q, C_{Ar}), 124.0 (+, 2C_{Ar}H), 123.8 (+, C_{Ar}H), 123.7 (+, C_{Ar}H), 121.9 (+, 2C_{Ar}H), 121.2 (+, C_{Ar}H), 121.1 (+, C_{Ar}H), 120.9 (+, C_{Ar}H), 115.9 (+, 2C_{Ar}H), 114.1 (+, 2C_{Ar}H), 1C is missing (1C, C–B)[5]. – **IR** (ATR): ṽ = 2921, 1604, 1585, 1560, 1483, 1453, 1375, 1333, 1324, 1309, 1292, 1265, 1238, 1204, 1156, 1130, 1116, 1102, 1074, 1060, 1041, 1023, 976, 970, 955, 933, 926, 909, 878, 861, 829, 812, 758, 737, 677, 659, 637, 622, 613, 603, 591, 550, 528, 487, 466, 455, 439, 424, 412, 398, 390, 378 cm^{-1}. – **MS** (FAB, 3-NBA), *m/z*: 452 [M+H]$^+$, 451 [M]$^+$. – **HRMS** ($C_{30}H_{18}N_1O_3{}^{11}B_1$) calc.: 451.1380; found: 451.1378.

<u>5,12-bis(9,9-dimethylacridin-10(9*N*)-yl)-8,9-dioxa-8a-borabenzo[fg]tetracene (DDMAC-OBO)</u>

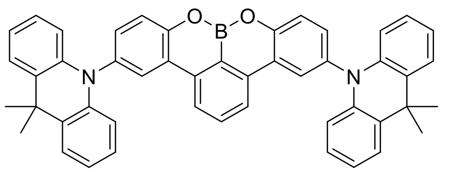

To a solution of compound 10,10'-(6,6''-dimethoxy-[1,1':3',1''-terphenyl]-3,3''-diyl)bis(9,9-dimethyl-9,10-dihydroacridine) (1.40 g, 1.99 mmol, 1.00 equiv.) in anhydrous dichlorobenzene (50 mL) was added tribromoborane (995 mg, 3.97 mL, 3.97 mmol, 1.00 M in heptane, 2.00 equiv.) under argon. Then the mixture was heated to 150 °C and stirred at this temperature for 12 h. After quenching with methanol, the reaction mixture was concentrated under reduced pressure. The obtained crude product was purified *via* flash-chromatography on silica gel using cyclohexane/dichloromethane 2:1 to 1:1 to yield the product as a white solid (320 mg, 467 µmol, 24%).

R$_f$ = 0.28 (cyclohexane/dichloromethane = 2:1). – **Mp** was not observed (> 345 °C) – **^{1}H NMR** (500 MHz, THF-d_8, ppm) δ = 8.32 (d, *J* = 2.4 Hz, 2H), 8.27 (d, *J* = 8.0 Hz, 2H), 7.88 (t, *J* = 7.9 Hz, 1H), 7.75 (d, *J* = 8.5 Hz, 2H), 7.48 (dd, *J* = 7.7 Hz, *J* = 1.6 Hz, 4H), 7.46 (dd, *J* = 8.5 Hz, *J* = 2.4 Hz, 2H), 6.90 (m, 8H), 6.37 (dd, *J* = 8.2 Hz, *J* = 1.3 Hz, 4H), 1.70 (s, 12H). – **^{13}C NMR** (126 MHz, THF-d_8, ppm) δ = 152.3 (C_q, 2C_{Ar}), 141.9 (C_q, 4C_{Ar}), 139.6 (C_q, 2C_{Ar}), 137.3 (C_q, 2C_{Ar}), 134.8 (+, C_{Ar}H), 133.2 (+, 2C_{Ar}H), 130.7 (C_q, 4C_{Ar}), 128.1 (+, 2C_{Ar}H), 126.9 (+, 4C_{Ar}H), 126.7 (+, 2C_{Ar}H), 125.8 (+, 4C_{Ar}H), 123.5 (+, 2C_{Ar}H), 121.7 (+, 2C_{Ar}H), 121.2 (+, 4C_{Ar}H), 114.7 (+, 4C_{Ar}H), 36.6 (Cq, 2*C*CAr (CH_3)$_2$), 31.5 (+, 4CH_3), 1C is missing (1C, C–B)[5]. – **IR** (ATR): ʋ̃ = 2925, 1591, 1558, 1499, 1473, 1460, 1449, 1378, 1329, 1316, 1264, 1242, 1211, 1173, 1120, 1072, 1047, 929, 834, 815, 739, 652, 629, 596, 579, 517, 487, 469, 428 cm^{-1}. – **MS** (FAB, 3-NBA), *m/z*: 685 $[M+H]^+$, 684 $[M]^+$. – **HRMS** ($C_{48}H_{37}N_2O_2{}^{11}B_1$) calc.: 684.2948; found: 684.2948.

5,12-di(10*N*-phenoxazin-10-yl)-8,9-dioxa-8a-borabenzo[fg]tetracene (DPXZ-OBO)

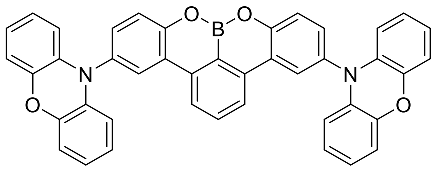

To a solution of compound 10,10'-(6,6''-dimethoxy-[1,1':3',1''-terphenyl]-3,3''-diyl)bis(10*N*-phenoxazine) (634 mg, 972 µmol, 1.00 equiv.) in anhydrous dichlorobenzene (30 mL) was added tribromoborane (487 mg, 1.94 mL, 1.94 mmol, 1.00 M in heptane, 2.00 equiv.) under argon. Then the mixture was heated to 150 °C and stirred at this temperature for 12 h. After quenching with methanol, the reaction mixture was concentrated under reduced pressure. The obtained crude product was purified *via* flash-chromatography on silica gel using cyclohexane/dichloromethane 2:1 to 1:1 to yield the product as a yellow solid (128 mg, 202 µmol, 21%).

R$_f$ = 0.58 (cyclohexane/dichloromethane = 1.5:1).). – **Mp =** 304 °C. – **^{1}H NMR** (500 MHz, THF-d_8, ppm) δ = 8.38 (d, *J* = 2.5 Hz, 2H), 8.31 (d, *J* = 7.9 Hz, 2H), 7.93 (t, *J* = 7.9 Hz, 1H), 7.71 (d, *J* = 8.5 Hz, 2H), 7.49 (dd, *J* = 8.6 Hz, *J* = 2.4 Hz, 2H), 6.68 (dd, *J* = 7.8 Hz, *J* = 1.6 Hz, 4H), 6.62 (td, *J* = 7.6 Hz, *J* = 1.6 Hz, 4H), 6.58 (td, *J* = 7.6 Hz, *J* = 1.7 Hz, 4H), 6.03 (dd, *J* = 7.8 Hz, *J* = 1.5 Hz, 4H). – **^{13}C NMR** (101 MHz, THF-d_8, ppm) δ = 152.4 (C_q, 2C_{Ar}), 144.8 (C_q, 4C_{Ar}), 139.5 (C_q, 2C_{Ar}), 135.4 (C_q, 4C_{Ar}), 135.0 (C_q, 2C_{Ar}), 134.9 (+, C_{Ar}H), 132.7 (+, 2C_{Ar}H), 127.8 (+, 2C_{Ar}H), 126.9 (C_q, 2C_{Ar}), 124.0 (+, 4C_{Ar}H), 123.8 (+, 2C_{Ar}H), 121.9 (+, 4C_{Ar}H), 121.8 (+, 2C_{Ar}H), 115.9 (+, 4C_{Ar}H), 114.1 (+, 4C_{Ar}H), 1C is missing (1C, C–B)[5]. – IR (ATR): ʋ̃ = 2922, 1605, 1588, 1562, 1486, 1456, 1377, 1339, 1330, 1316, 1292, 1265, 1203, 1153, 1119, 1103, 1095, 1075, 1043, 935,

914, 878, 871, 861, 829, 813, 778, 730, 674, 640, 630, 613, 601, 562, 492, 453, 426, 394 cm^{-1}. – **MS** (FAB, 3-NBA), *m/z*: 633 [M+H]$^{+}$, 632 [M]$^{+}$. – **HRMS** ($C_{42}H_{25}N_2O_4{}^{11}B_1$) calc.: 632.1907; found: 632.1907.

Crystal Structure Determinations

The single-crystal X-ray diffraction study weres carried out on a Bruker D8 Venture diffractometer with PhotonII detector at 123(2) K using Cu-Kα radiation (λ = 1.54178 Å). Dual space methods (SHELXT)[6] were used for structure solution and refinement was carried out using SHELXL-2014 (full-matrix least-squares on F^2.[7] Hydrogen atoms were localized by difference electron density determination and refined using a riding model. Semi-empirical absorption corrections were applied.

PXZ-OBO, sb1044_hy: yellow crystals, $C_{30}H_{18}BNO_3$, M_r = 451.26, crystal size 0.24 × 0.16 × 0.04 mm, triclinic, space group *P-1* (No. 2), a = 5.9979(3) Å, b = 13.5277(6) Å, c = 14.3850(7) Å, α = 64.594(2)°, β = 81.973(2)°, γ = 84.275(2)°, V = 1042.93(9) Å^3, Z = 2, ρ = 1.437 Mg/m^{-3}, μ(Cu-K$_\alpha$) = 0.74 mm^{-1}, F(000) = 468, $2\theta_{max}$ = 144.4°, 18499 reflections, of which 4107 were independent (R_{int} = 0.025), 317 parameters, R_1 = 0.039 (for 3686 I > 2σ(I)), wR_2 = 0.108 (all data), S = 1.06, largest diff. peak / hole = 0.73 / -0.19 e Å^{-3}.

5PXZ-OBO, sb1295_hy: yellow crystals, $C_{30}H_{18}BNO_3$, M_r = 451.26, crystal size 0.16 × 0.08 × 0.03 mm, orthorhombic, space group *P*bca (No. 61), a = 9.0398(2) Å, b = 17.2238(4) Å, c = 27.4998(6) Å, V = 4281.71(17) Å^3, Z = 8, ρ = 1.400 Mg/m^{-3}, μ(Cu-K$_\alpha$) = 0.72 mm^{-1}, F(000) = 1872, $2\theta_{max}$ = 144.2°, 39582 reflections, of which 4205 were independent (R_{int} = 0.048), 316 parameters, R_1 = 0.048 (for 3625 I > 2σ(I)), wR_2 = 0.129 (all data), S = 1.05, largest diff. peak / hole = 0.84 / -0.19 e Å^{-3}.

5DMAC-OBO, SB1097_hy: colourless crystals, $C_{32}H_{24}BNO_2$, M_r = 477.34, crystal size 0.24 × 0.12 × 0.06 mm, triclinic, space group *P-1* (No. 2), a = 8.8265(3) Å, b = 13.4334(4) Å, c = 20.8908(6) Å, α = 76.063(1)°, β = 83.015(1)°, γ = 84.063(1)°, V = 2379.18(13) Å^3, Z = 4, ρ = 1.333 Mg/m^{-3}, μ(Cu-K$_\alpha$) = 0.64 mm^{-1}, F(000) = 1000, $2\theta_{max}$ = 144.6°, 34843 reflections, of which 9321 were independent (R_{int} = 0.024), 667 parameters, R_1 = 0.037 (for 8383 I > 2σ(I)), wR_2 = 0.102 (all data), S = 1.03, largest diff. peak / hole = 0.31 / -0.19 e Å^{-3}.

CCDC 1973638 (**PXZ-OBO, sb1044_hy**), 1973639 (5**PXZ-OBO, sb1295_hy**), and 1973640 (**5DMAC-OBO, SB1097_hy**) contain the supplementary crystallographic data for this paper. These data can be obtained free of charge from The Cambridge Crystallographic Data Centre via www.ccdc.cam.ac.uk/data_request/cif.

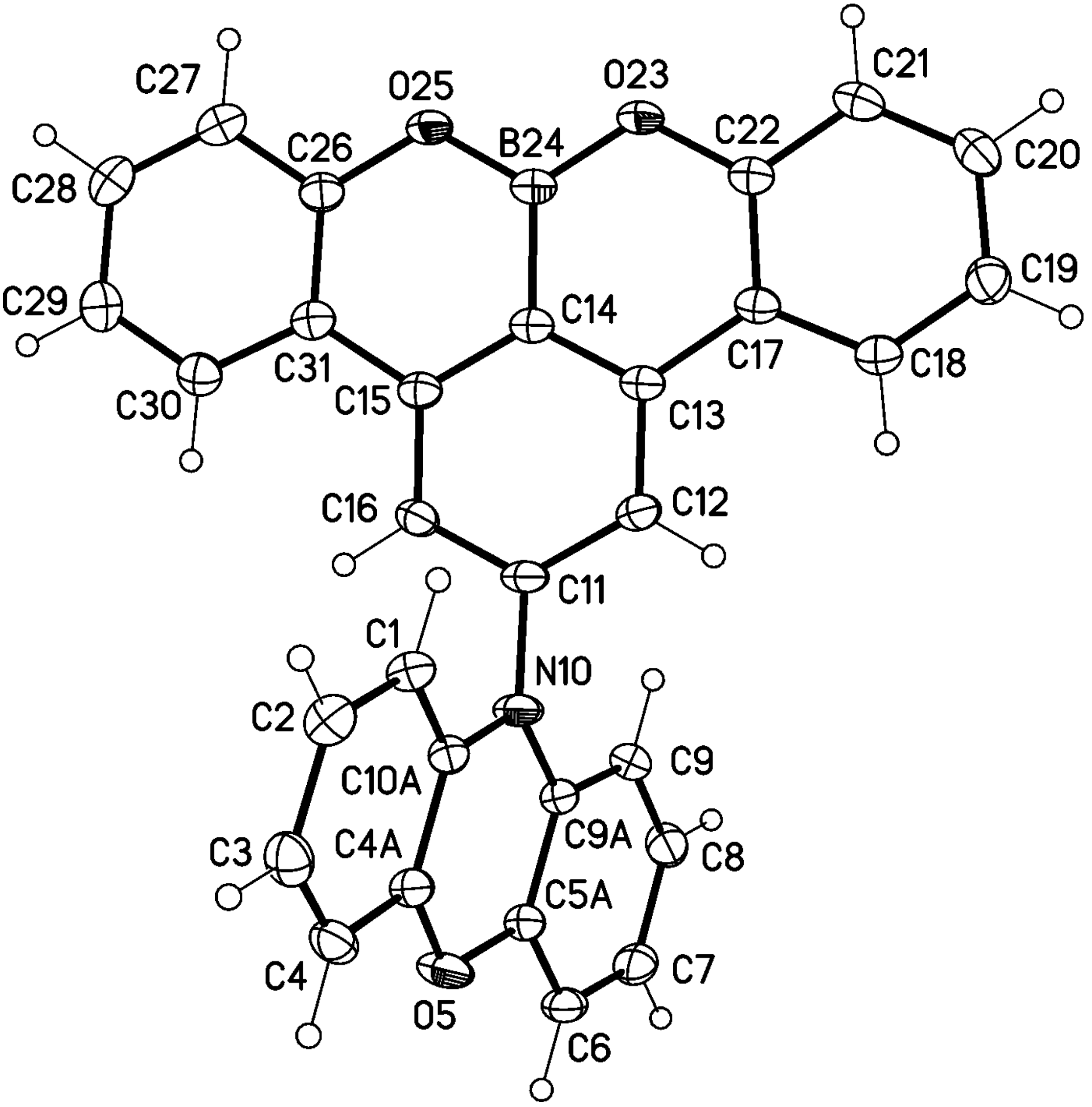

Fig. S1 Molecular structure of **PXZ-OBO, sb1044_hy** (displacement parameters are drawn at 50 % probability level).

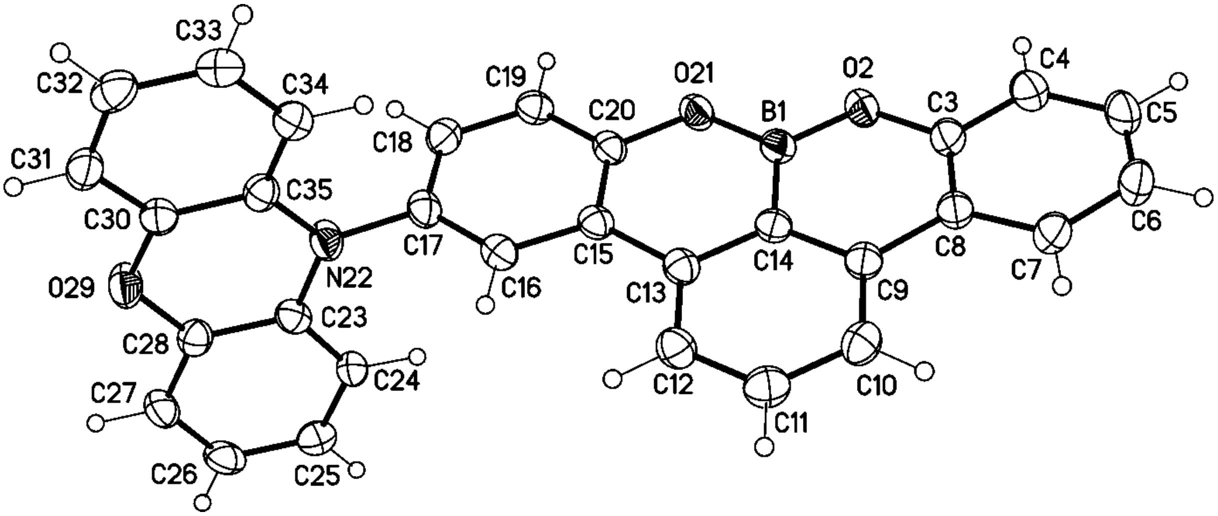

Fig. S2 Molecular structure of 5**PXZ-OBO, sb1295_hy** (displacement parameters are drawn at 50 % probability level).

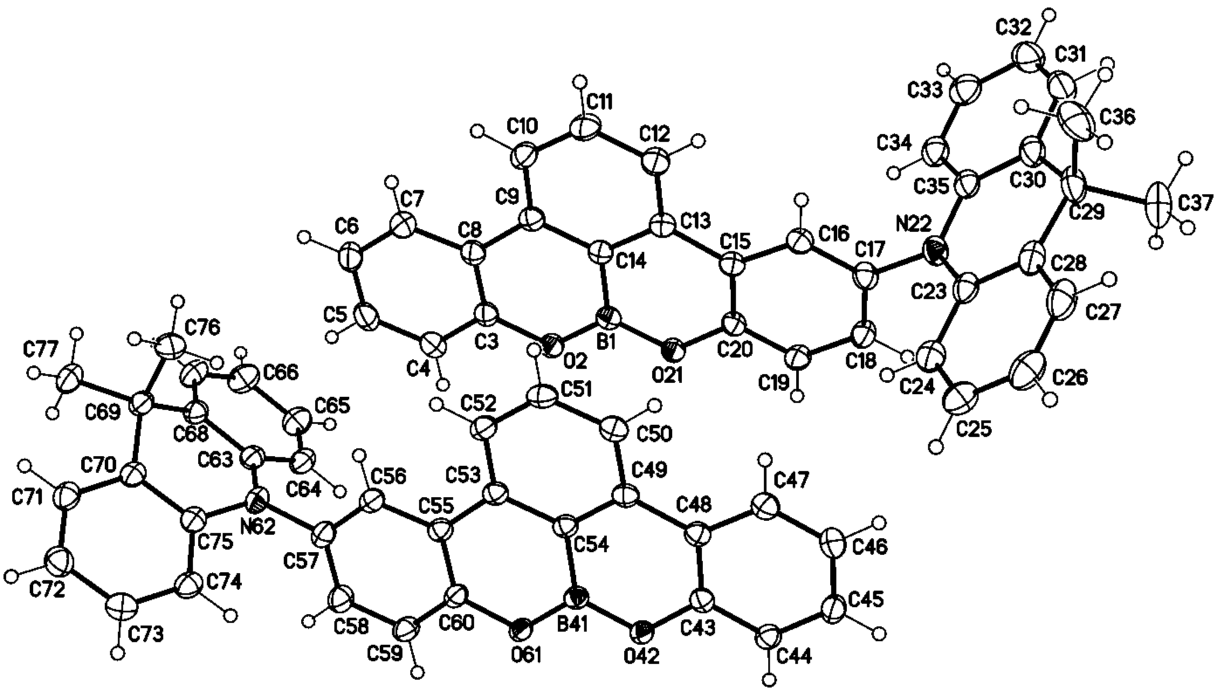

Fig. S3 Molecular structure of **5DMAC-OBO, SB1097_hy** (displacement parameters are drawn at 50 % probability level).

10-(3,5-dichlorophenyl)-9,9-dimethyl-9,10-dihydroacridine (1):

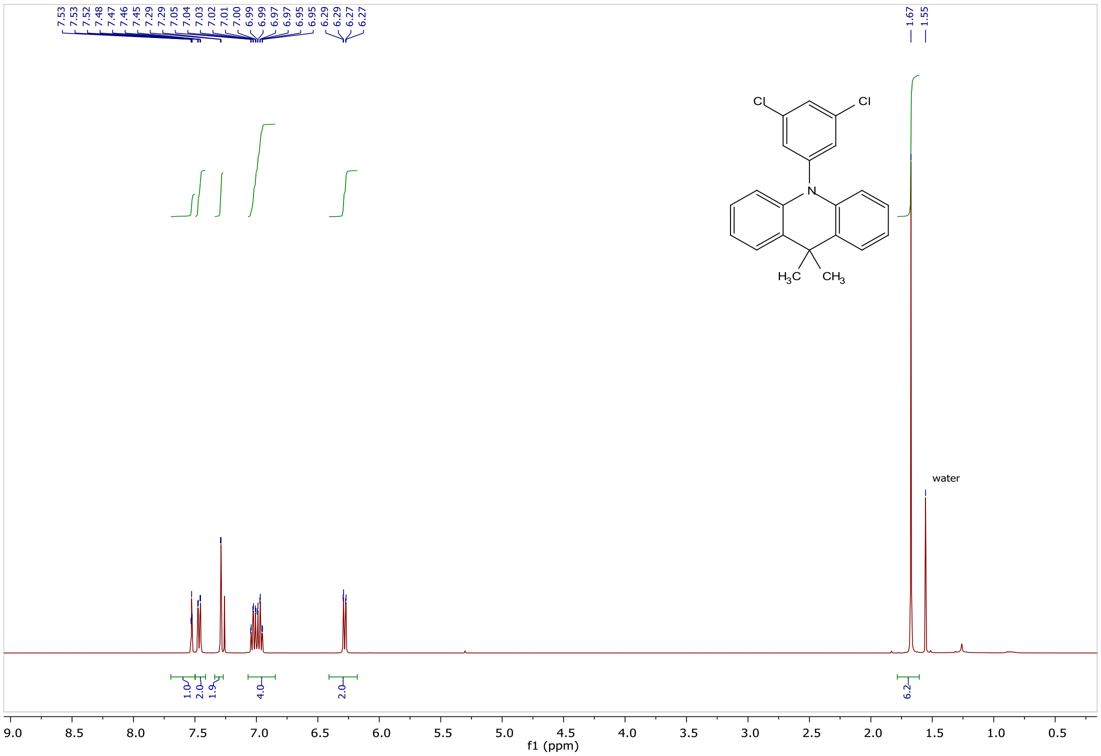

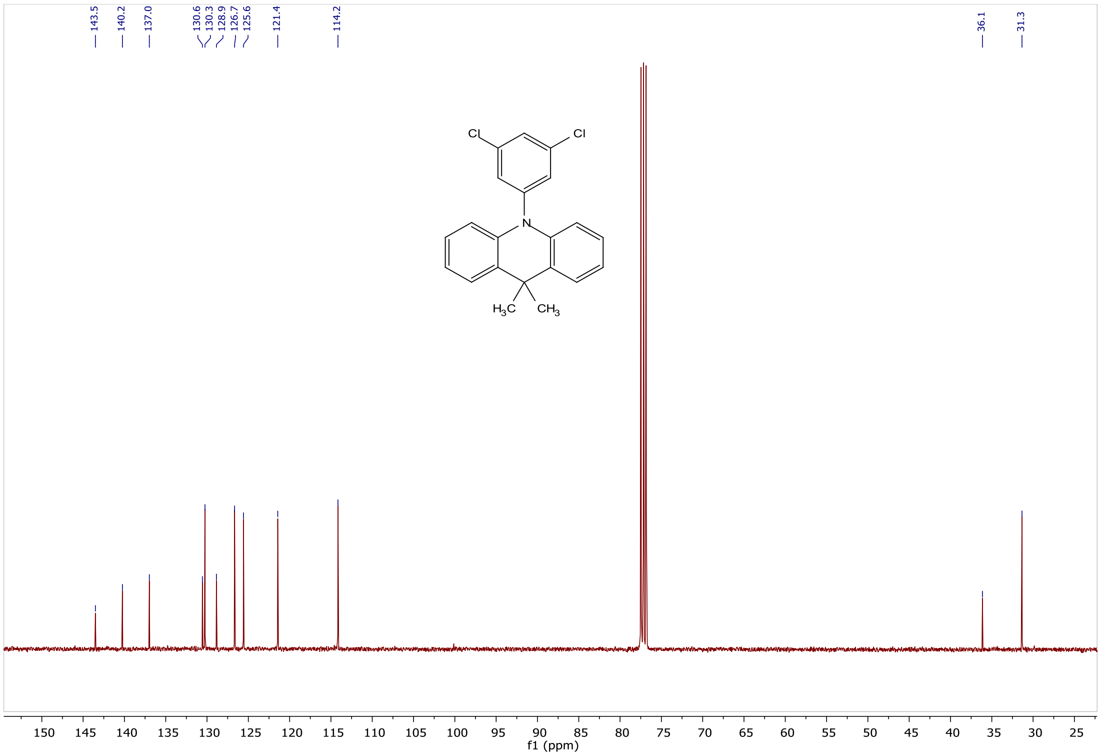

<u>10-(3,5-dichlorophenyl)-10*N*-phenoxazine (2):</u>

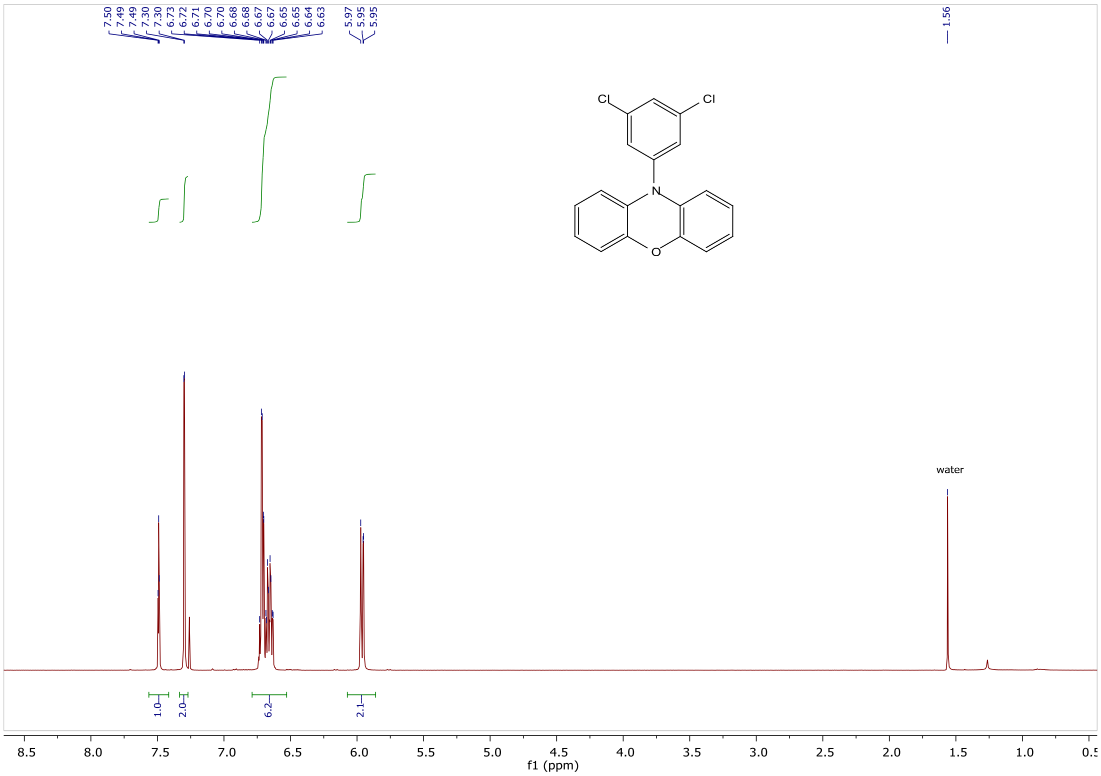

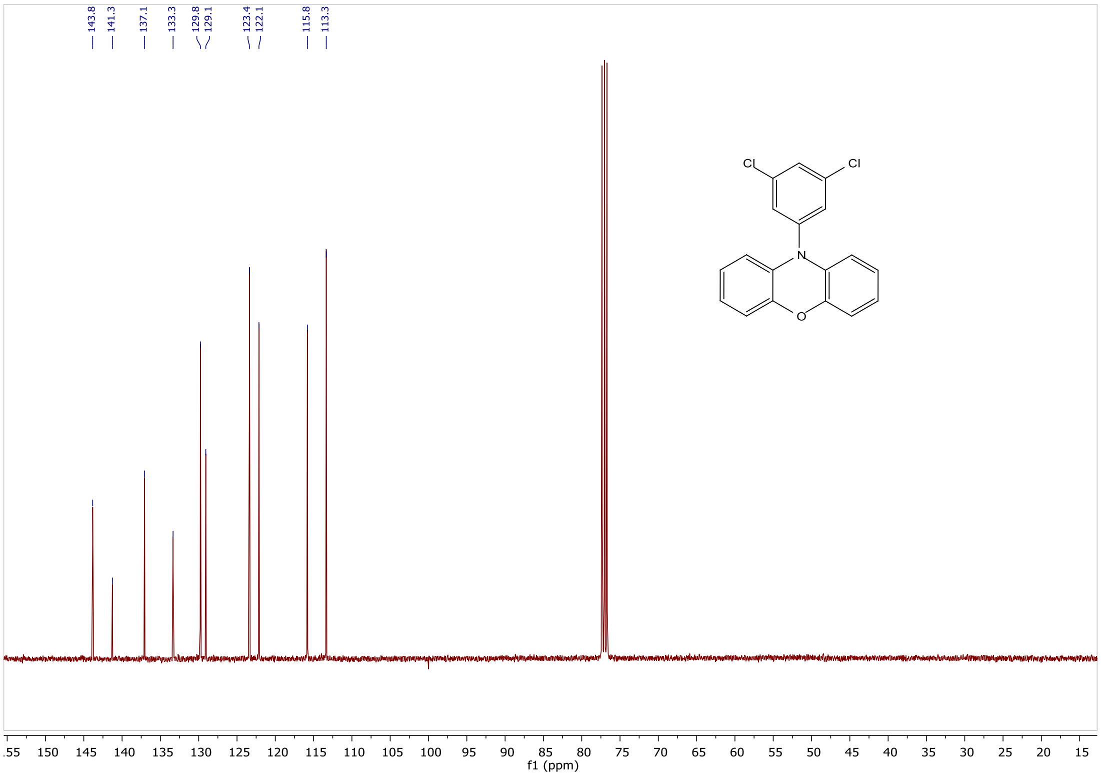

10-(2,2''-dimethoxy-[1,1':3',1''-terphenyl]-5'-yl)-9,9-dimethyl-9,10-dihydroacridine (3)

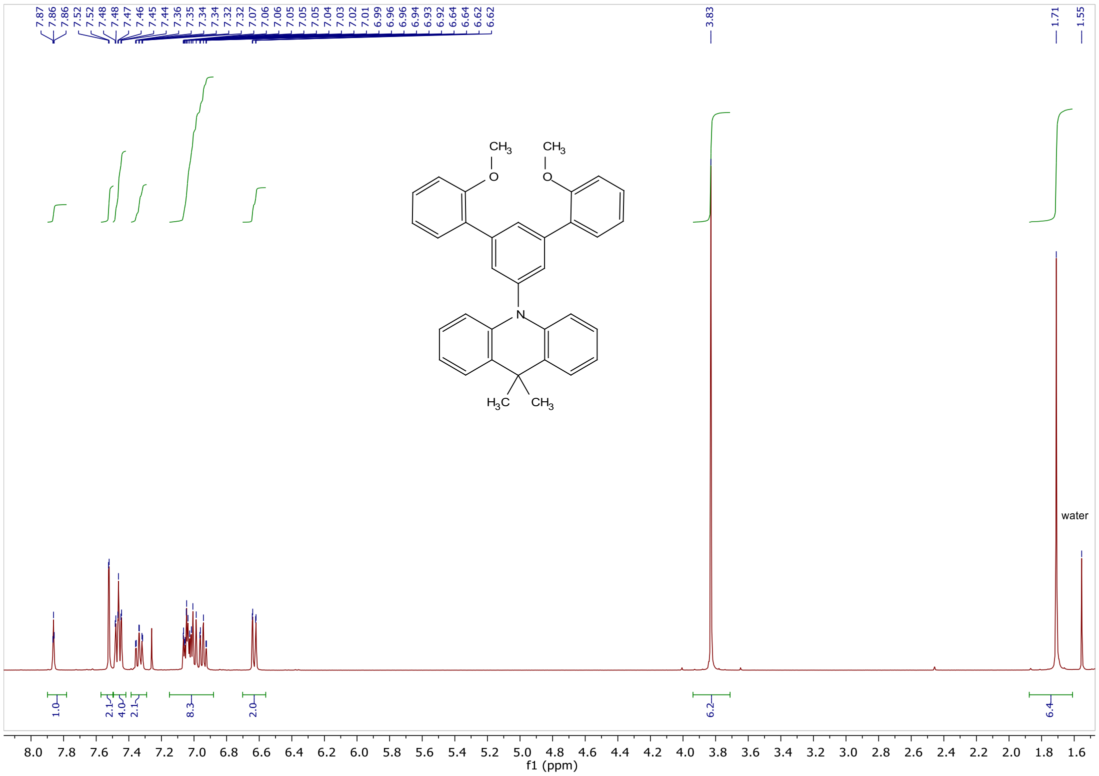

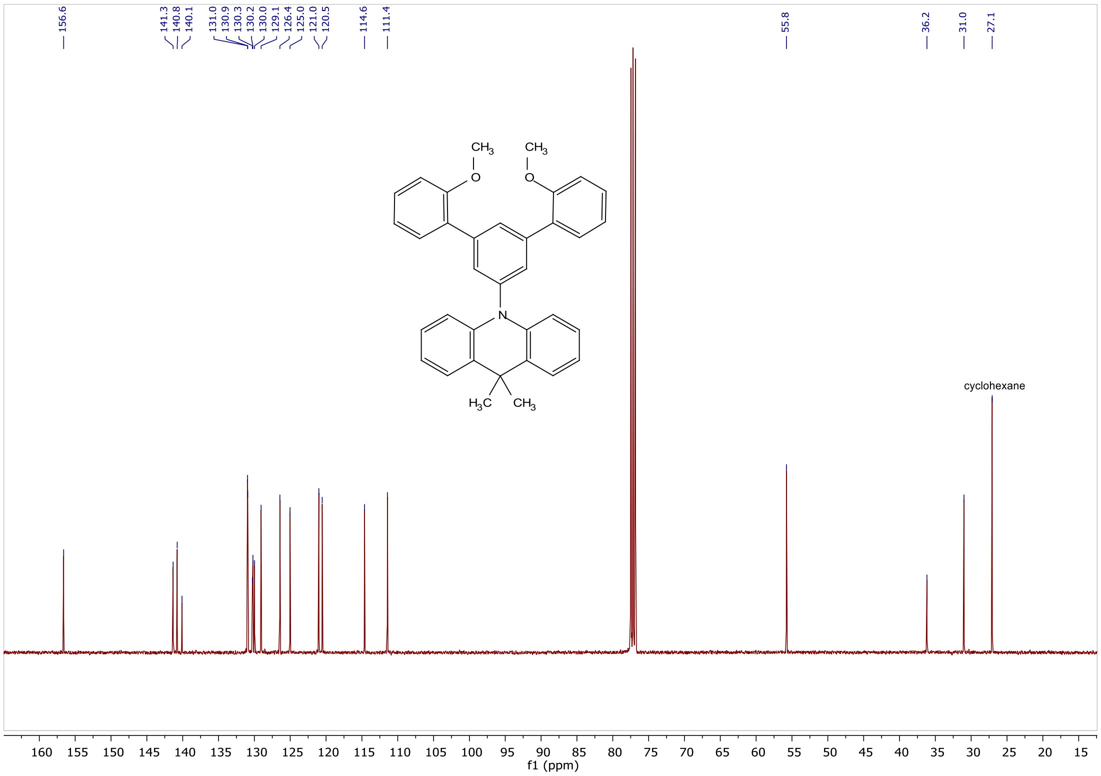

10-(2,2''-dimethoxy-[1,1':3',1''-terphenyl]-5'-yl)-10*N*-phenoxazine (4)

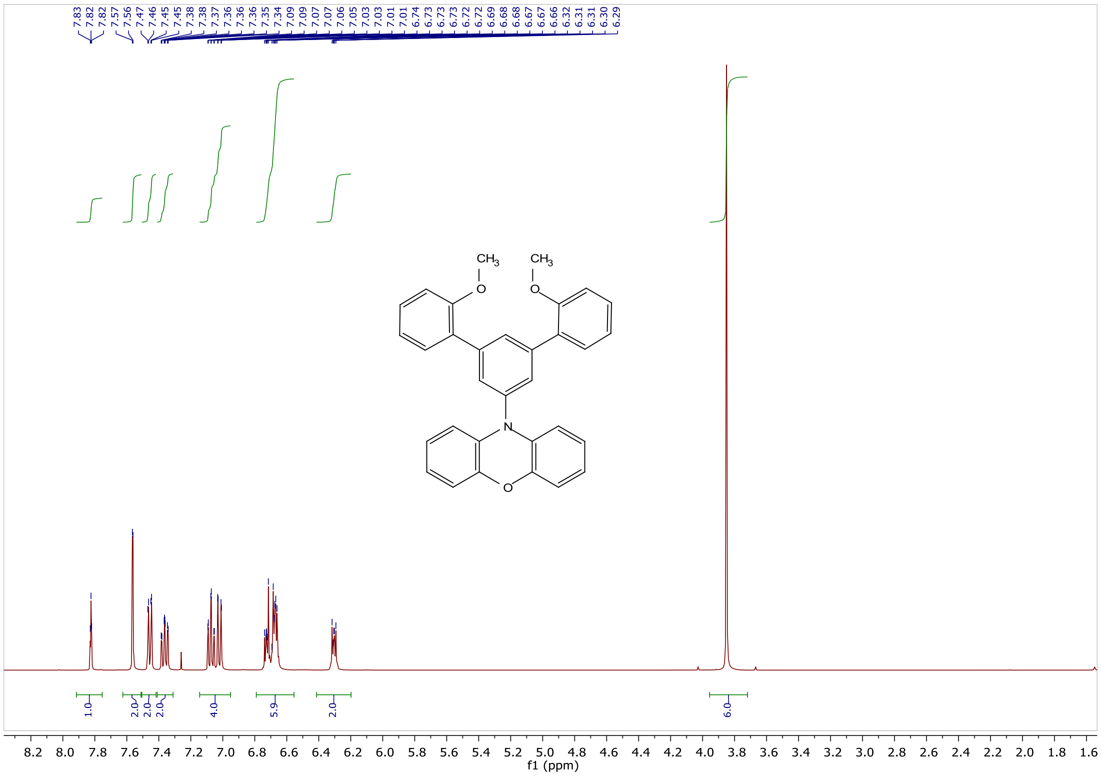

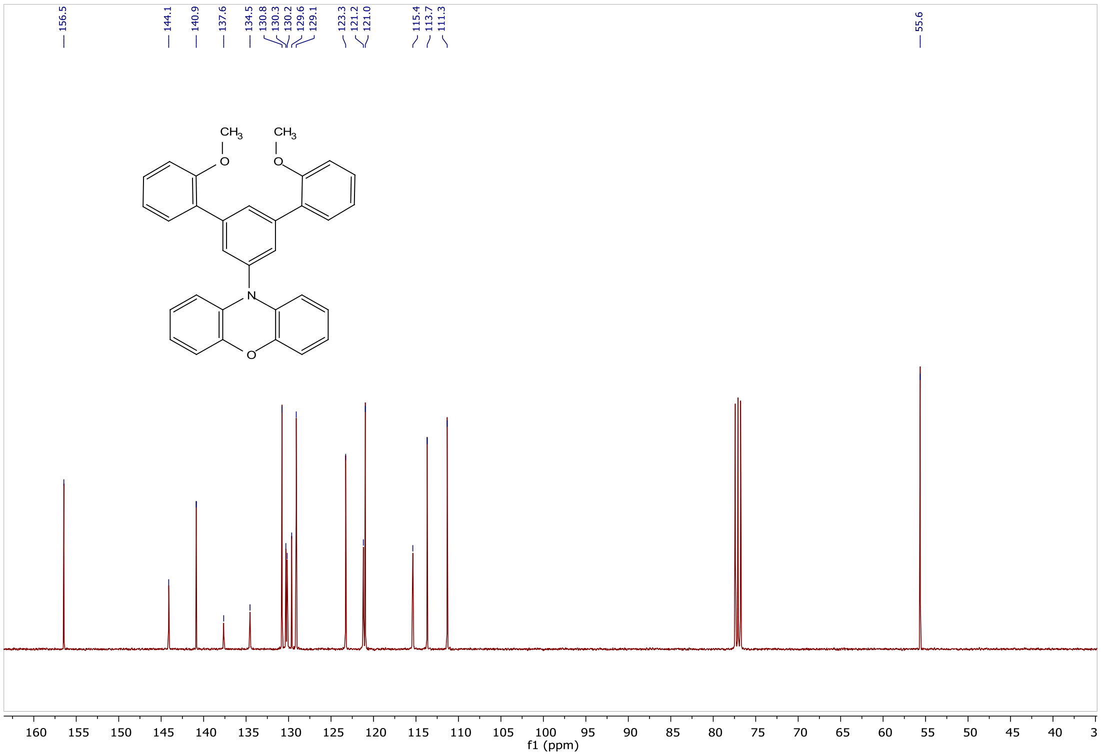

10-(8,9-dioxa-8a-borabenzo[fg]tetracen-2-yl)-9,9-dimethyl-9,10-dihydroacridine (DMAC-OBO)

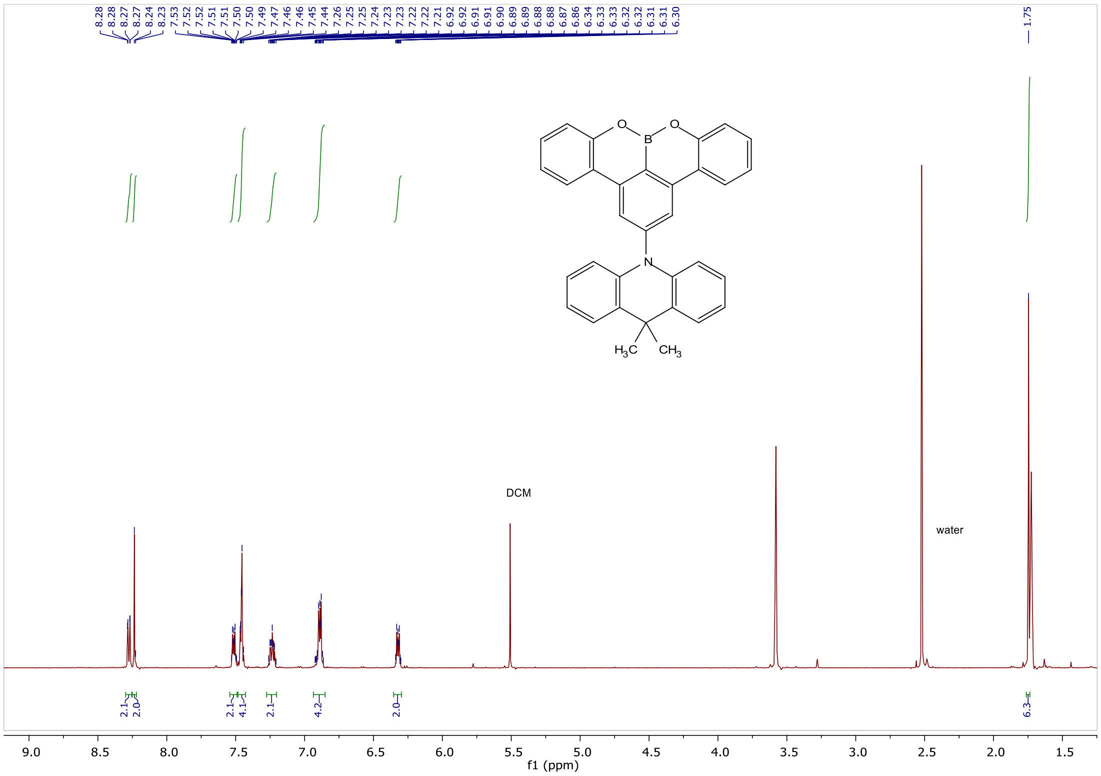

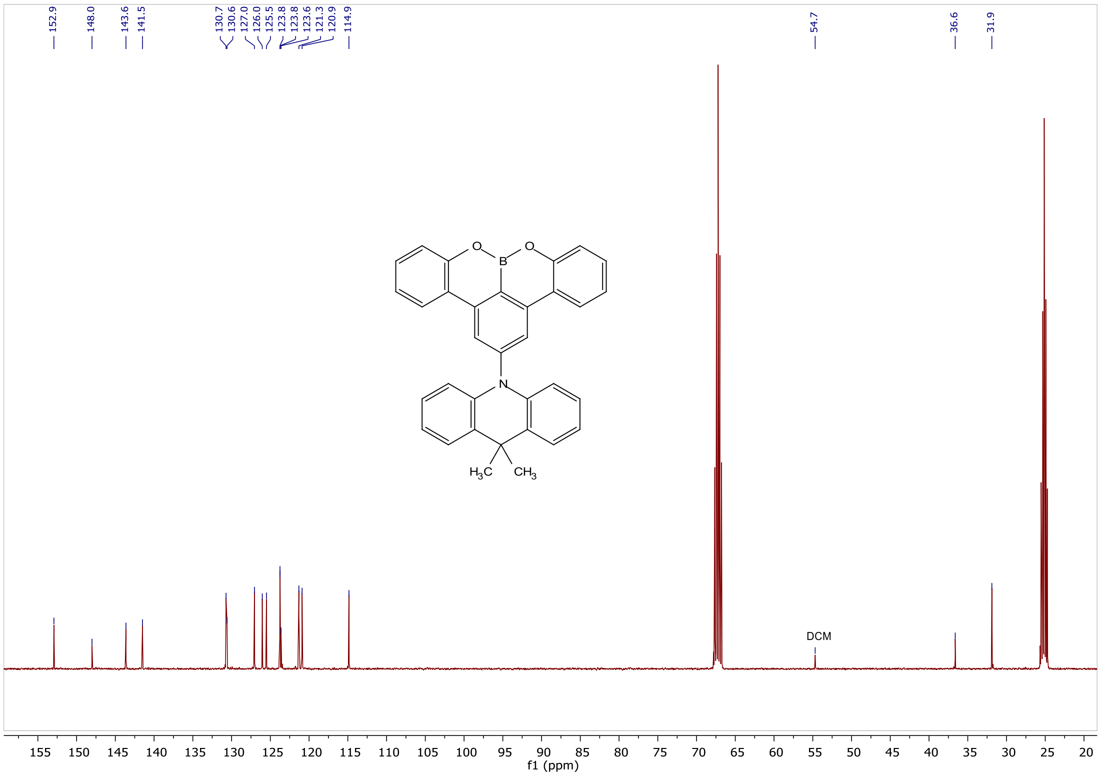

^{11}B NMR

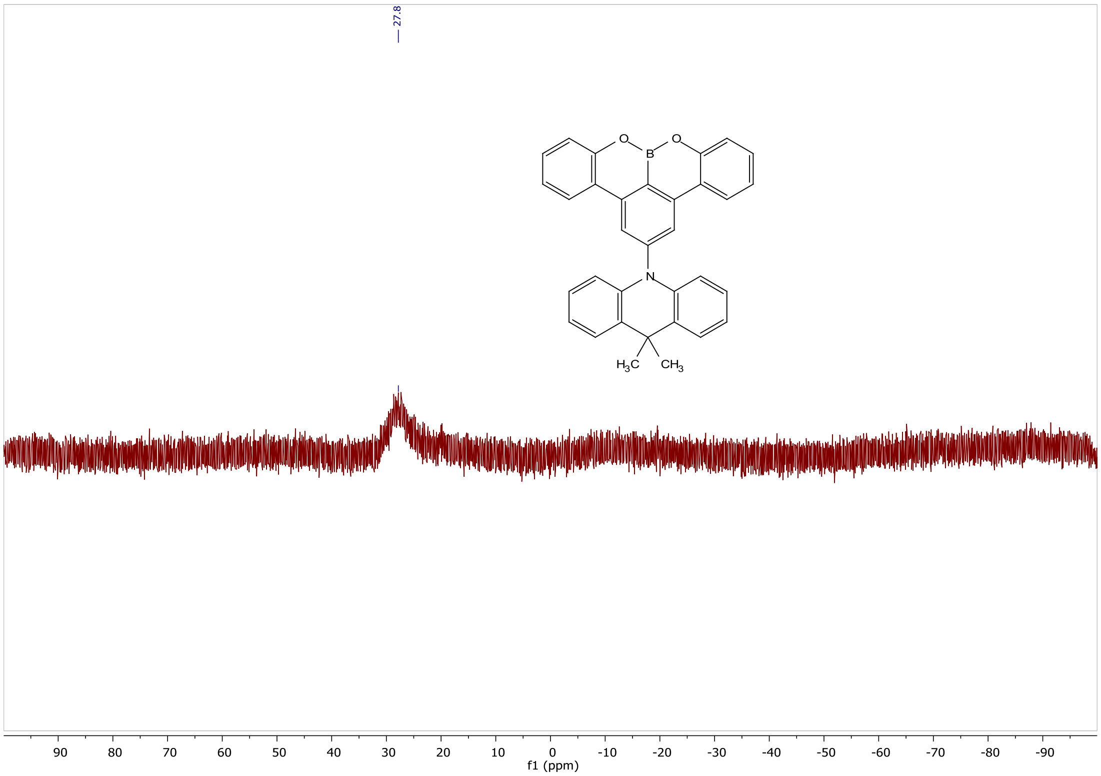

27.8
O
B
O
N
H_3C
CH_3
90
80
70
60
50
40
30
20
10
0
-10
-20
-30
-40
-50
-60
-70
-80
-90
f1 (ppm)

10-(8,9-dioxa-8a-borabenzo[fg]tetracen-2-yl)-10*N*-phenoxazine (PXZ-OBO)

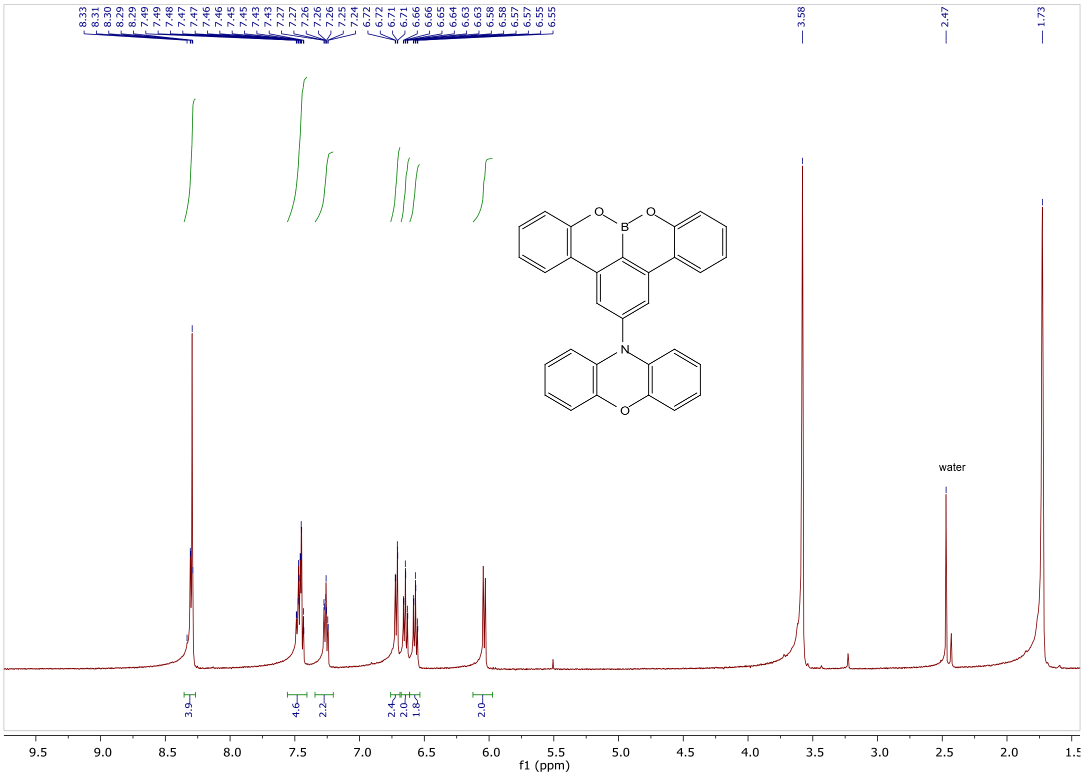

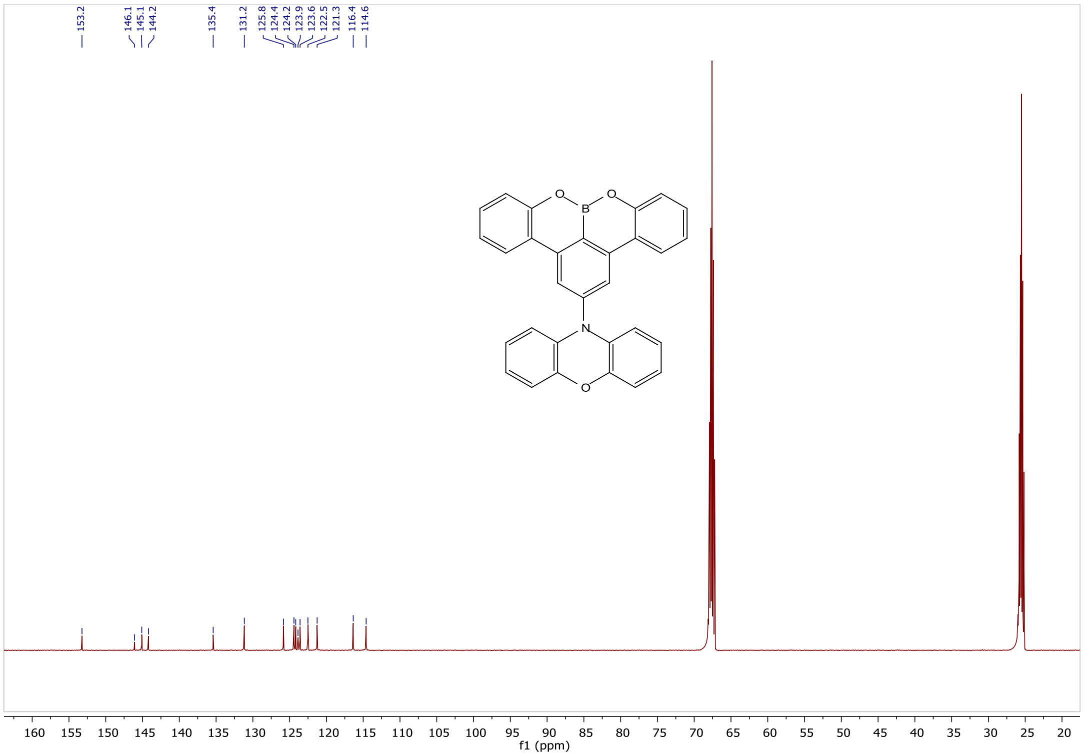

^{11}B NMR

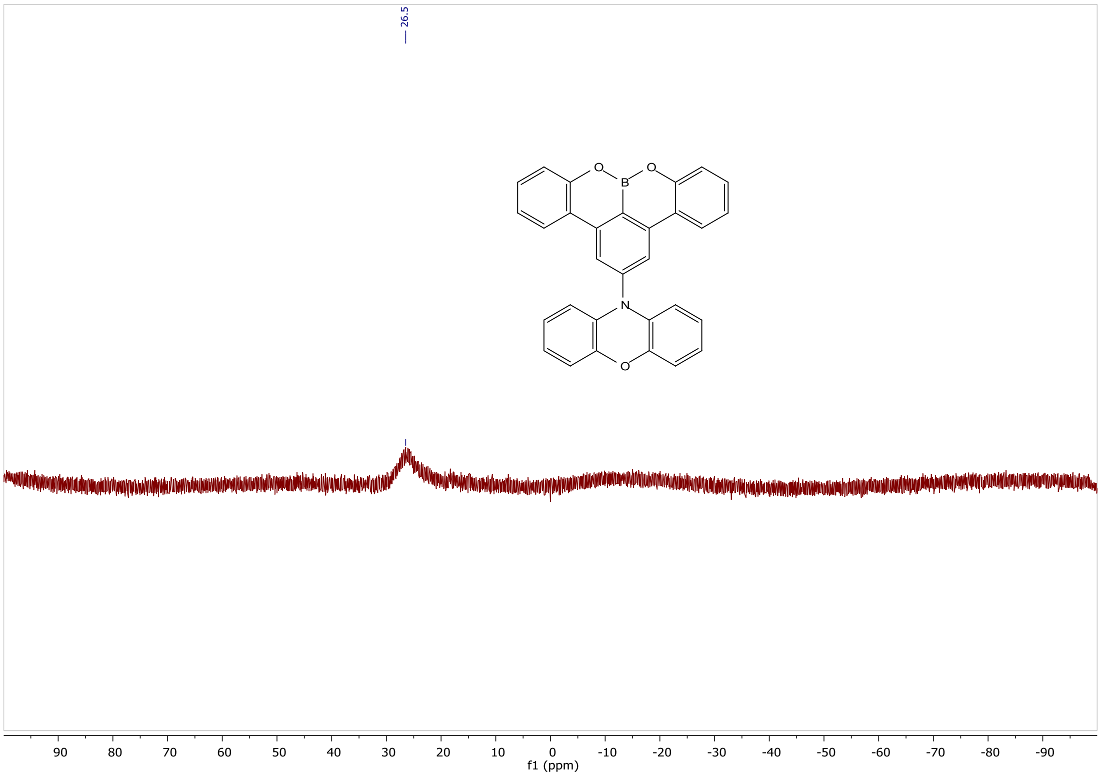

26.5
O
B
O
N
O
90
80
70
60
50
40
30
20
10
0
-10
-20
-30
-40
-50
-60
-70
-80
-90
f1 (ppm)

10-(3-chloro-4-methoxyphenyl)-9,9-dimethyl-9,10-dihydroacridine (5)

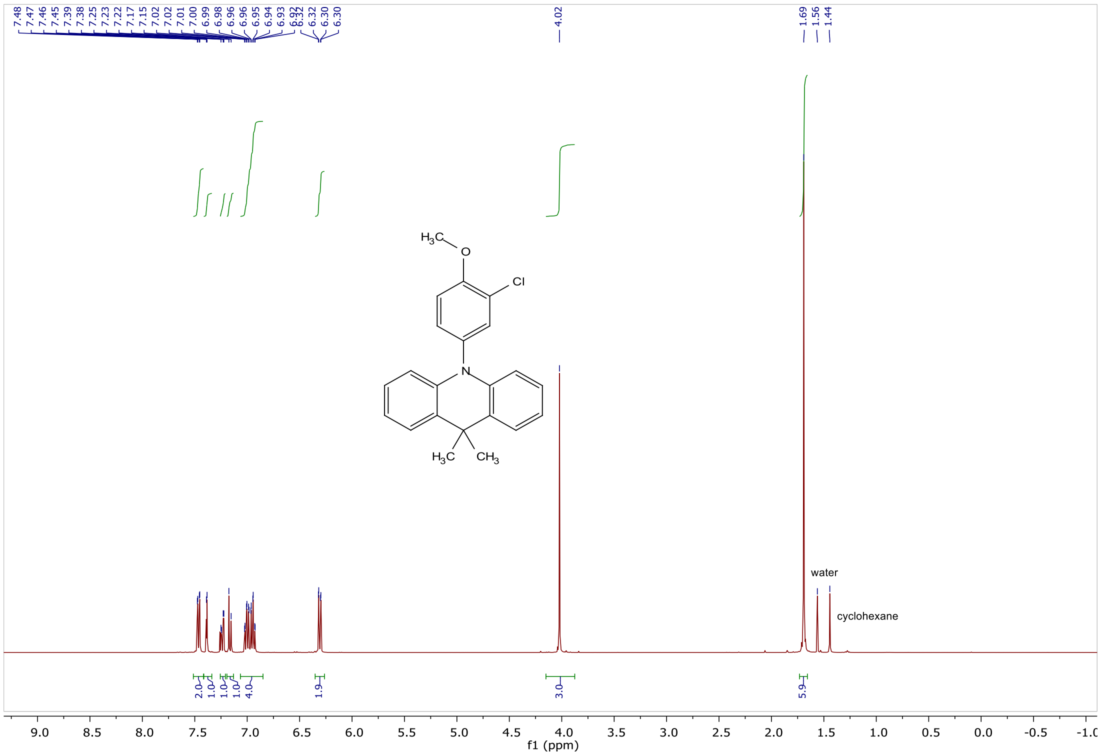

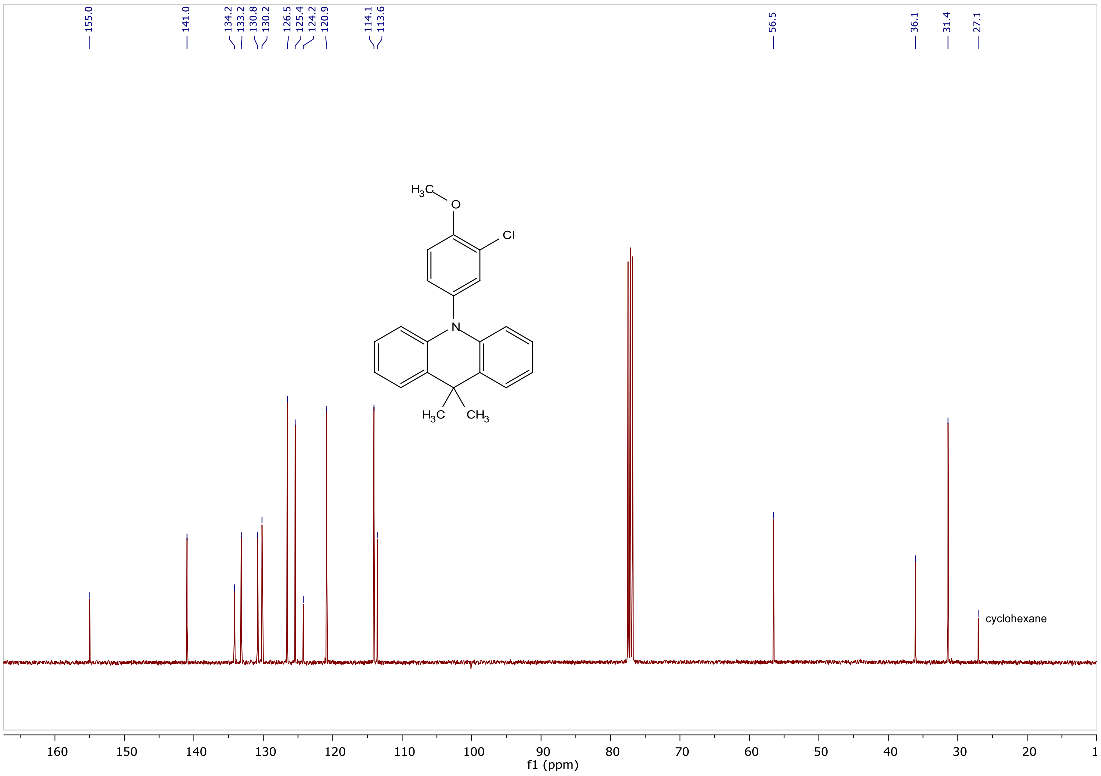

10-(3-chloro-4-methoxyphenyl)-10*N*-phenoxazine (6)

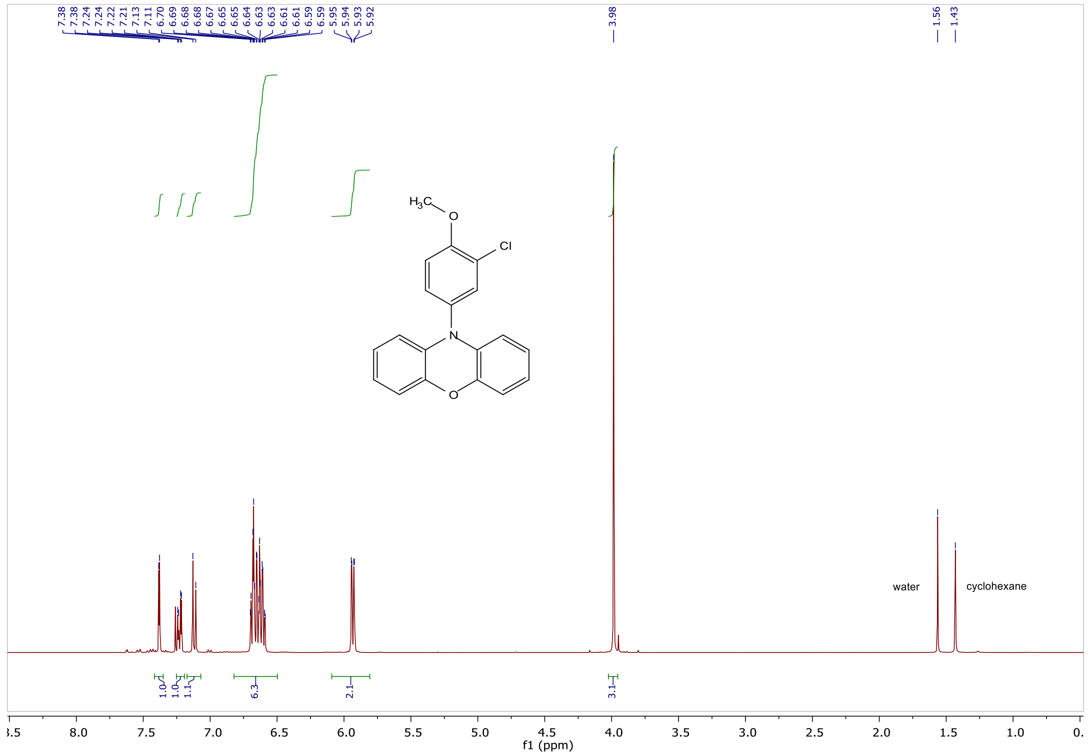

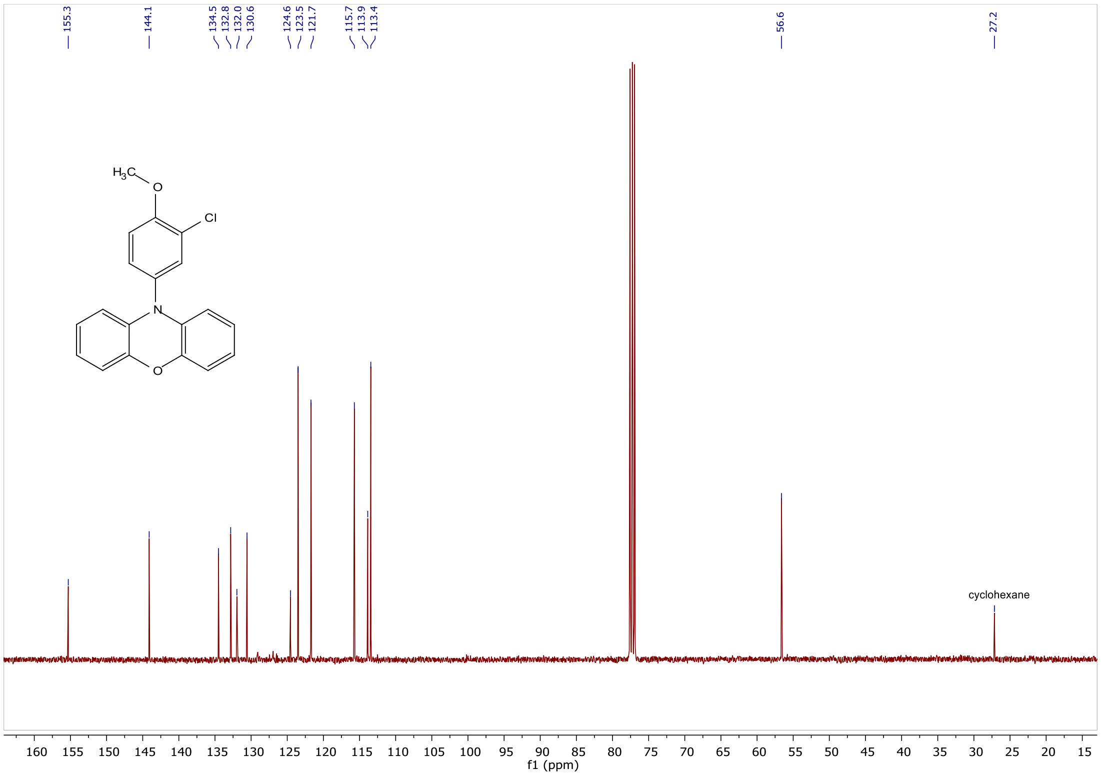

10-(2'',6-dimethoxy-[1,1':3',1''-terphenyl]-3-yl)-9,9-dimethyl-9,10-dihydroacridine (7)

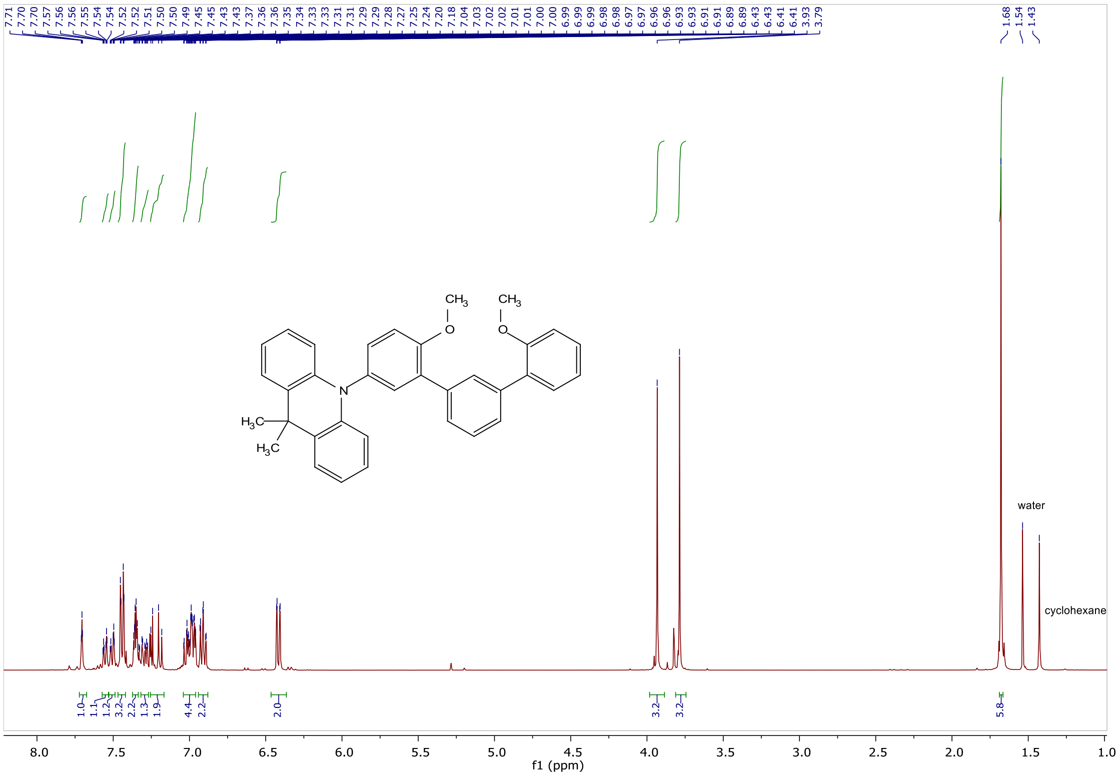

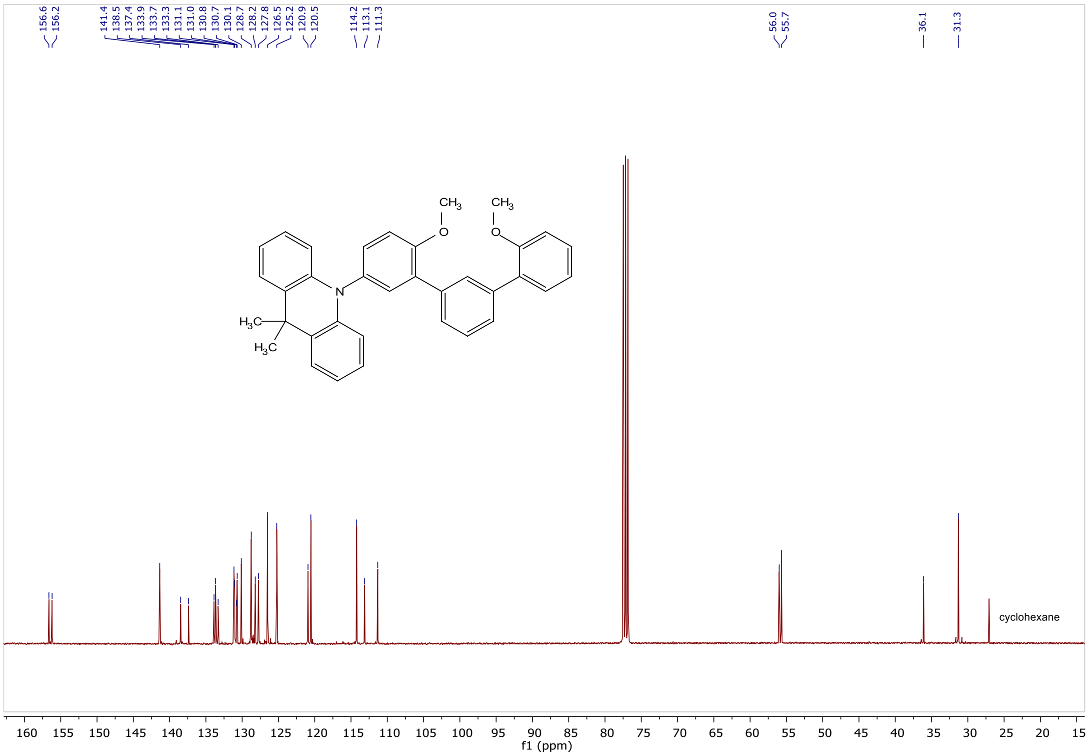

10-(2'',6-dimethoxy-[1,1':3',1''-terphenyl]-3-yl)-10*N*-phenoxazine (8)

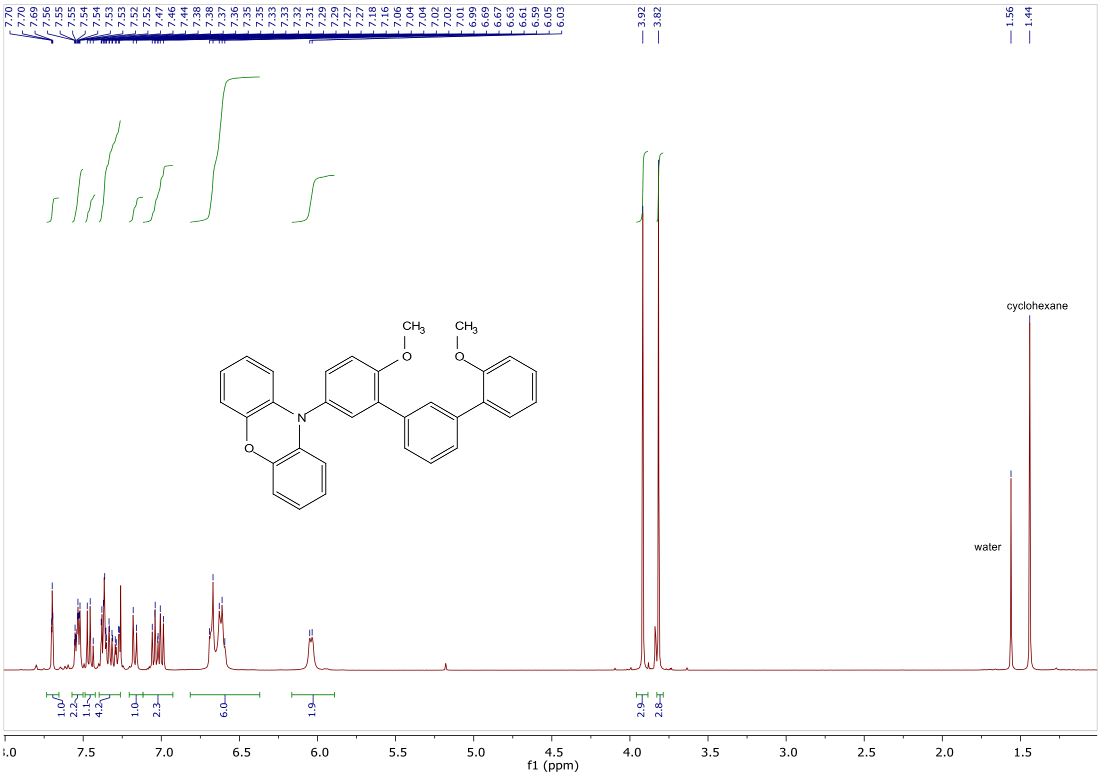

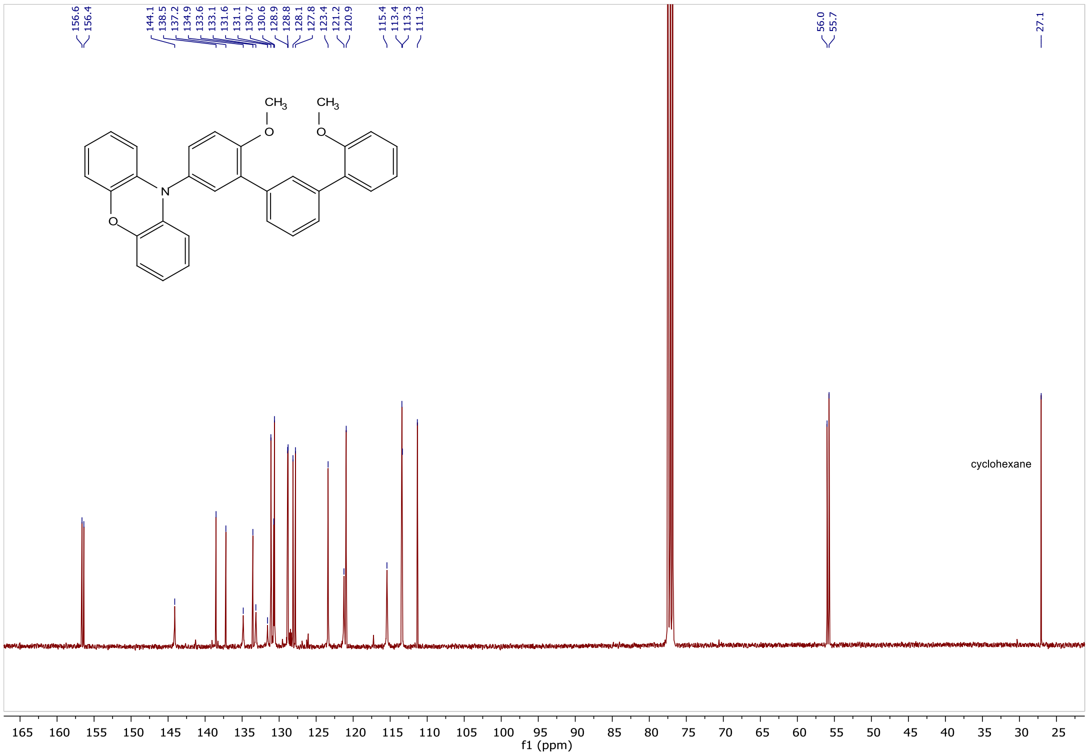

10,10'-(6,6''-dimethoxy-[1,1':3',1''-terphenyl]-3,3''-diyl)bis(9,9-dimethyl-9,10 dihydroacridine) (9)

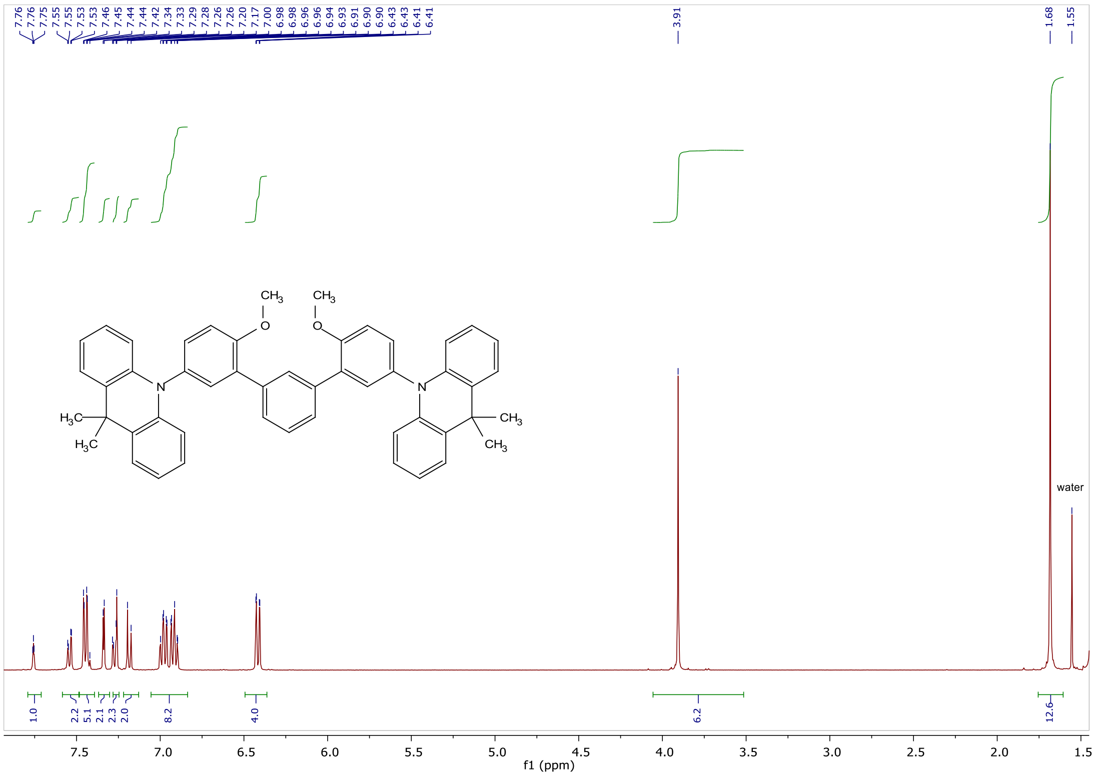

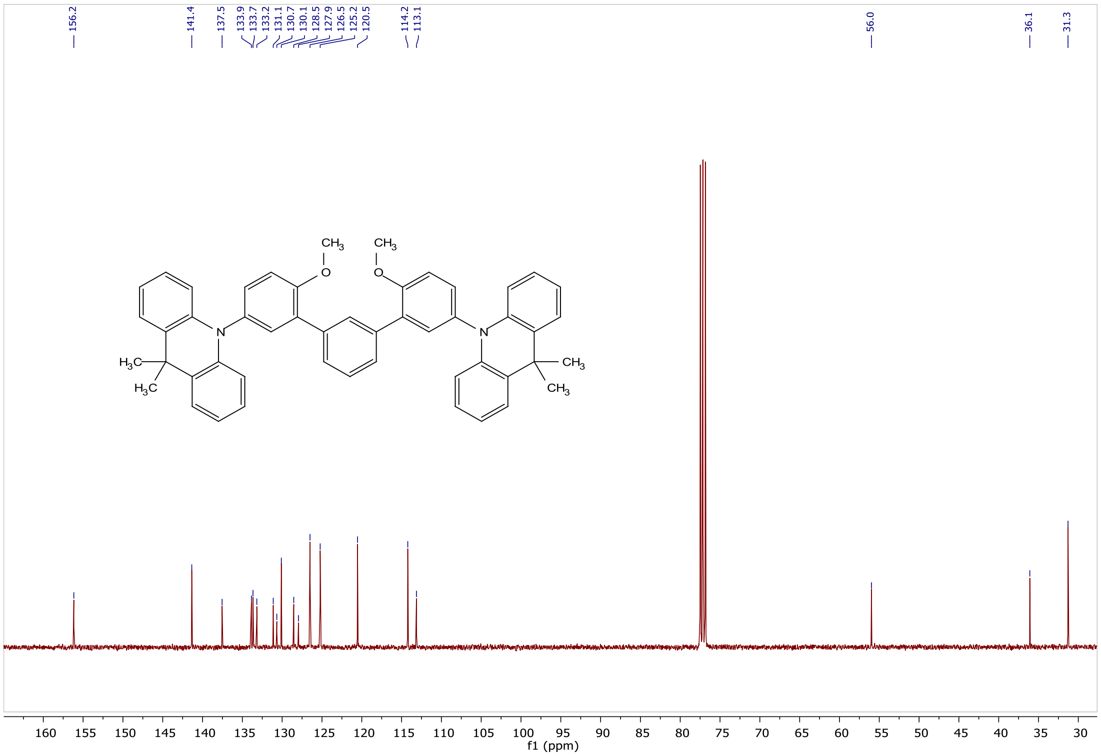

10,10'-(6,6''-dimethoxy-[1,1':3',1''-terphenyl]-3,3''-diyl)bis(10*N*-phenoxazine) (10)

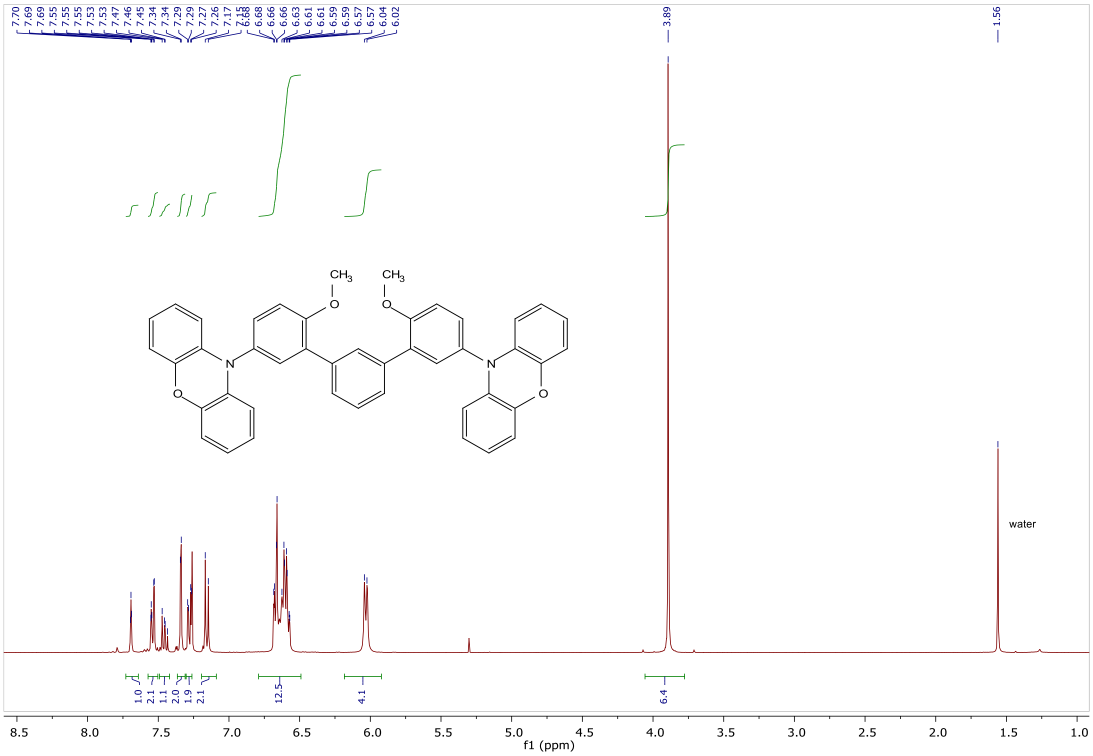

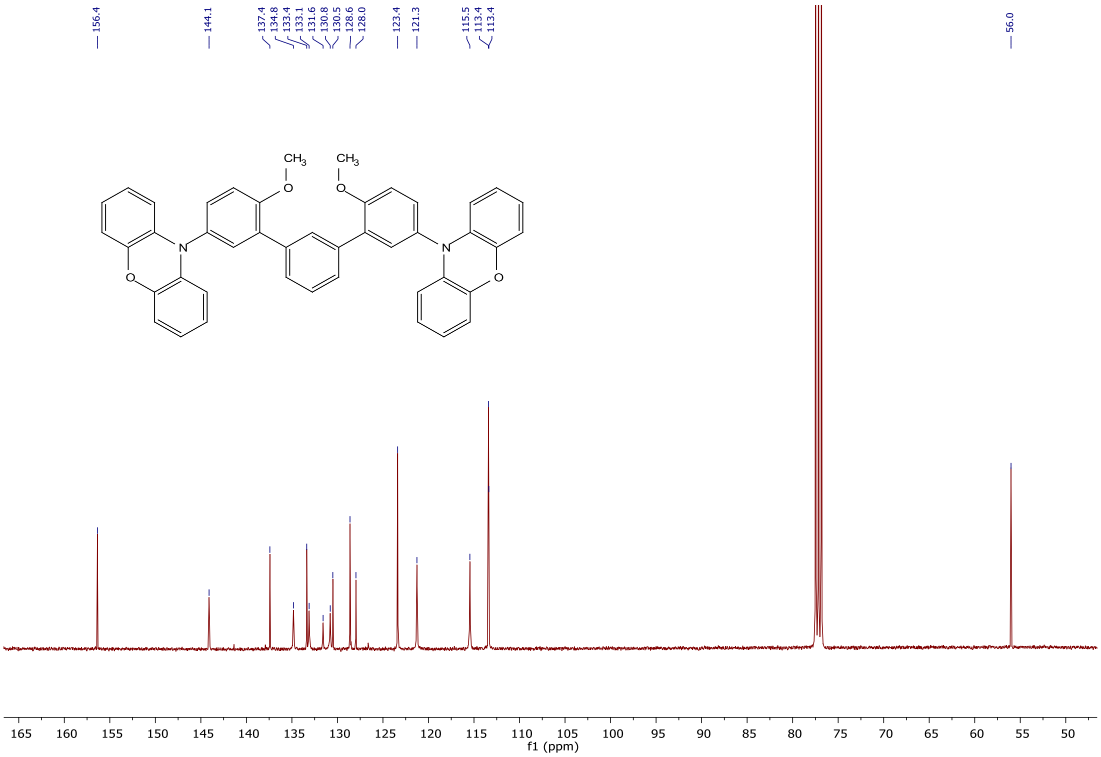

10-(8,9-dioxa-8a-borabenzo[fg]tetracen-12-yl)-9,9-dimethyl-9,10-dihydroacridine (5DMAC-OBO)

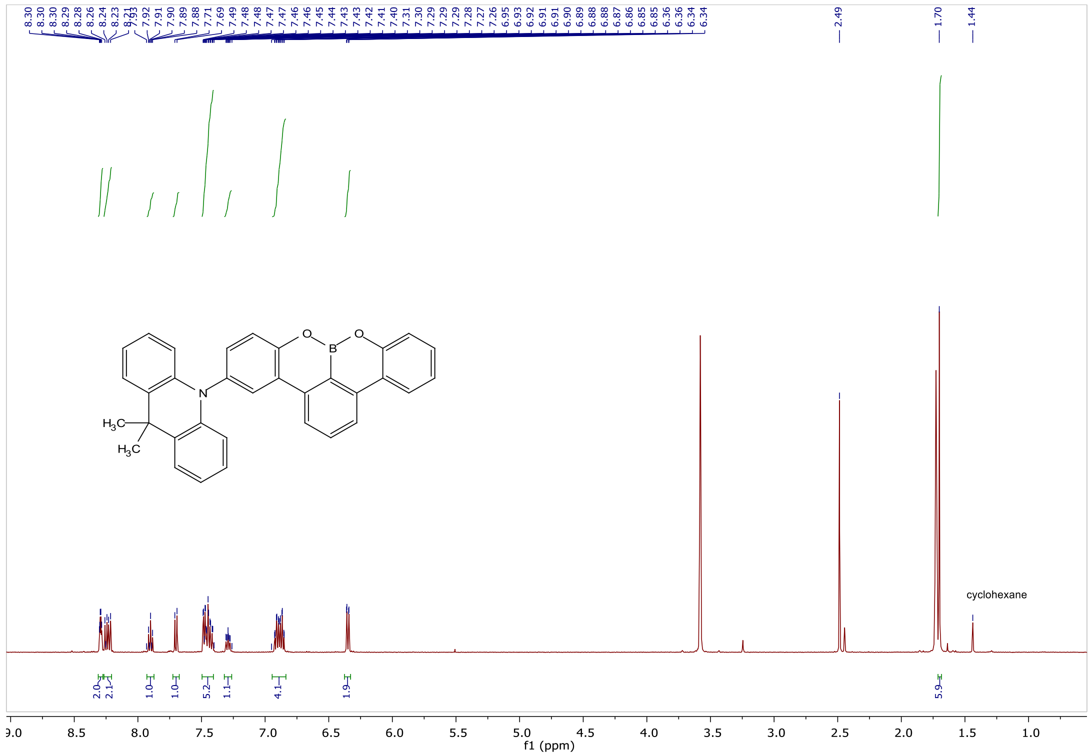

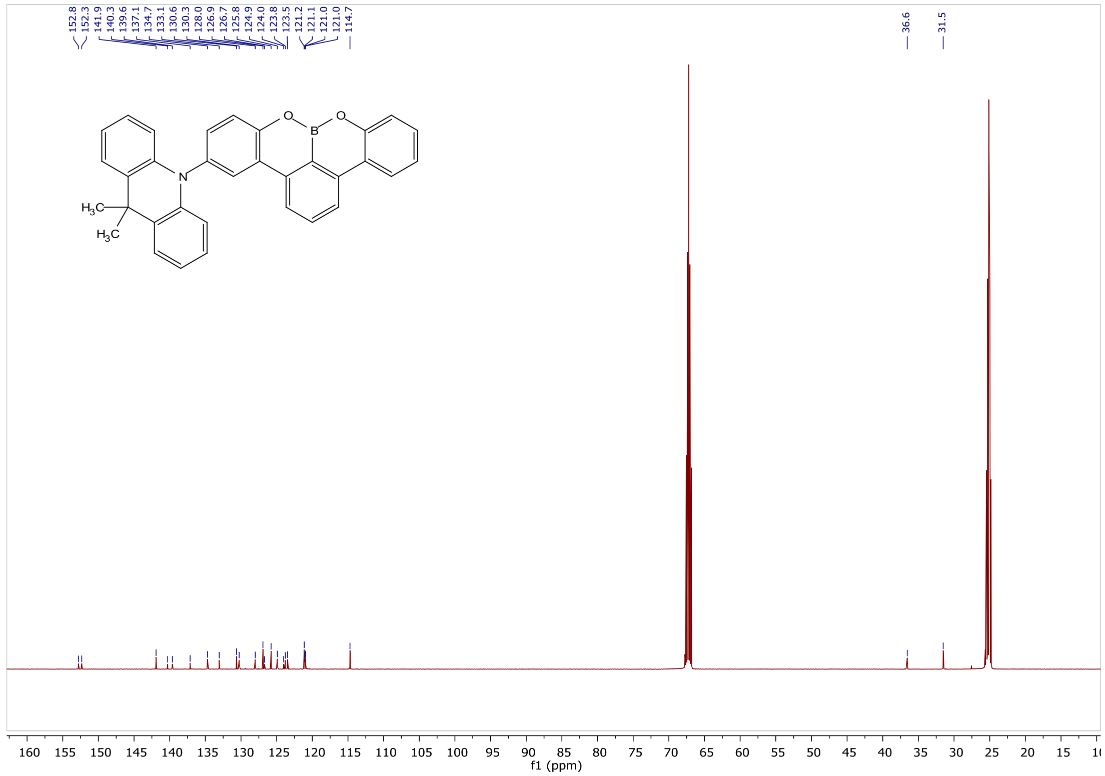

10-(8,9-dioxa-8a-borabenzo[fg]tetracen-12-yl)-10*N*-phenoxazine (5PXZ-OBO)

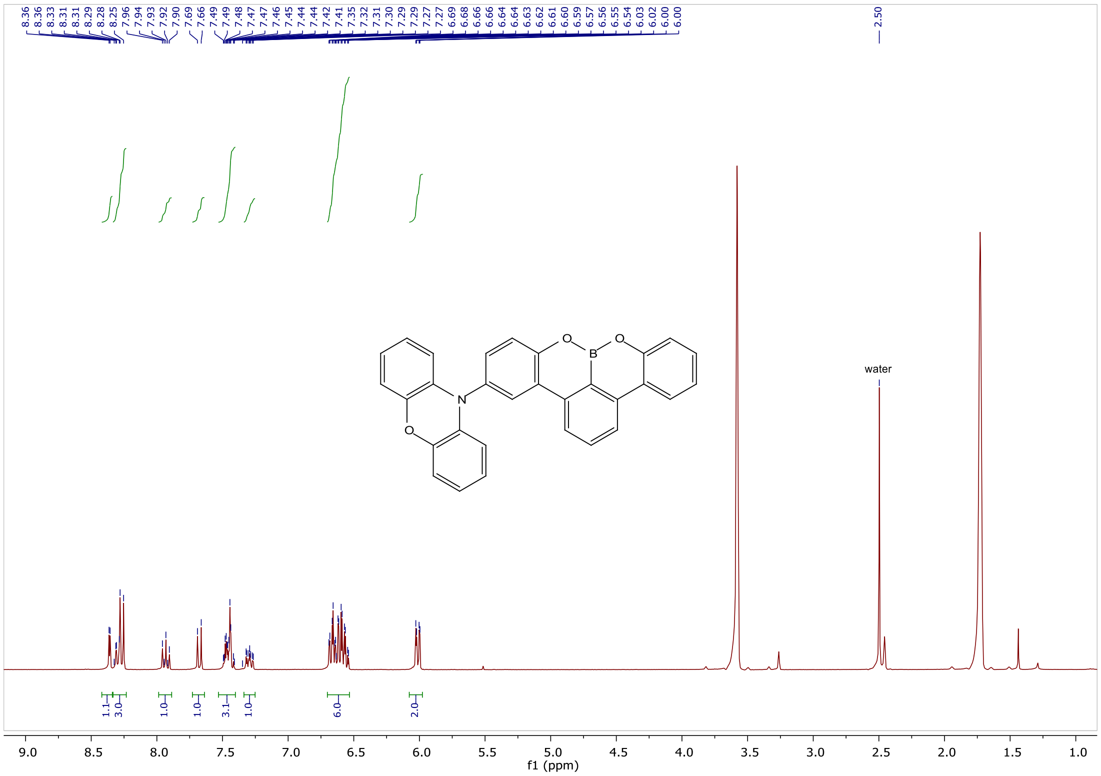

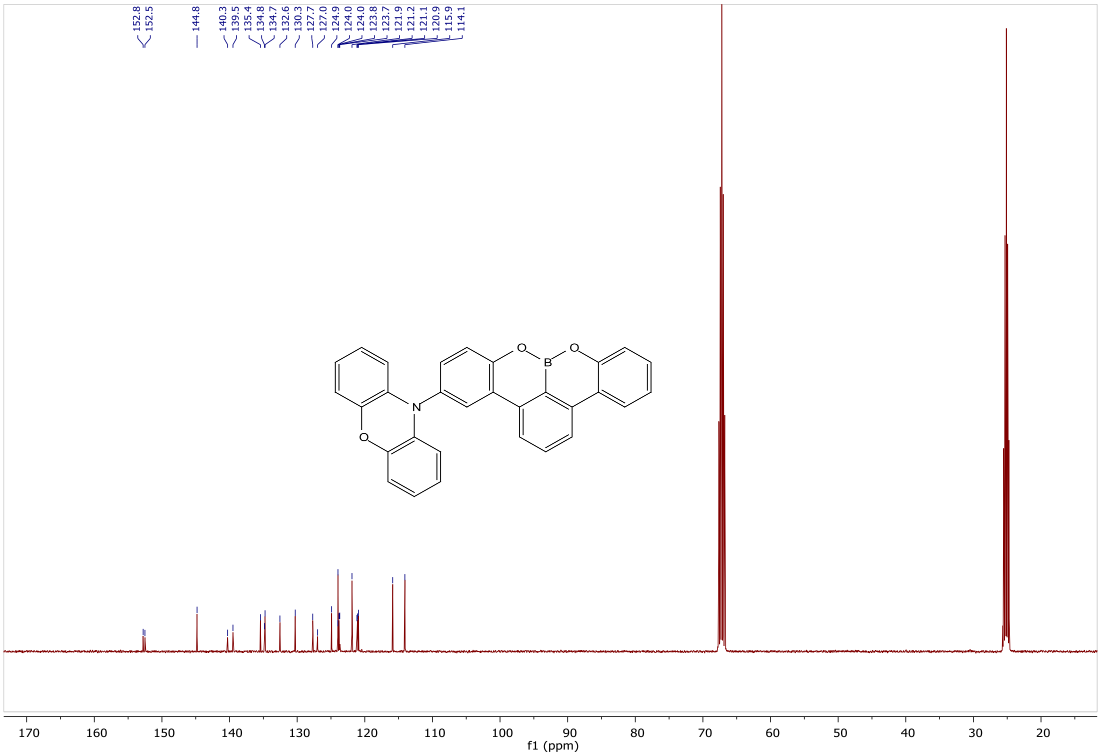

5,12-bis(9,9-dimethylacridin-10(9*N*)-yl)-8,9-dioxa-8a-borabenzo[fg]tetracene (DDMAC-OBO)

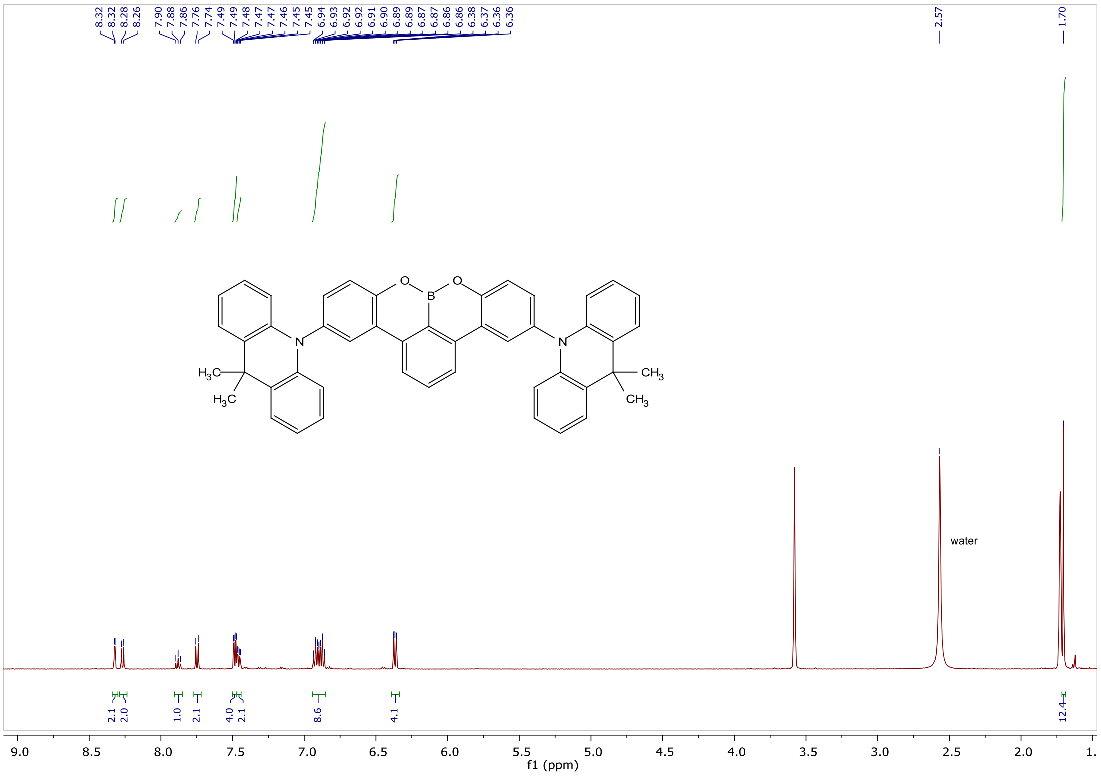

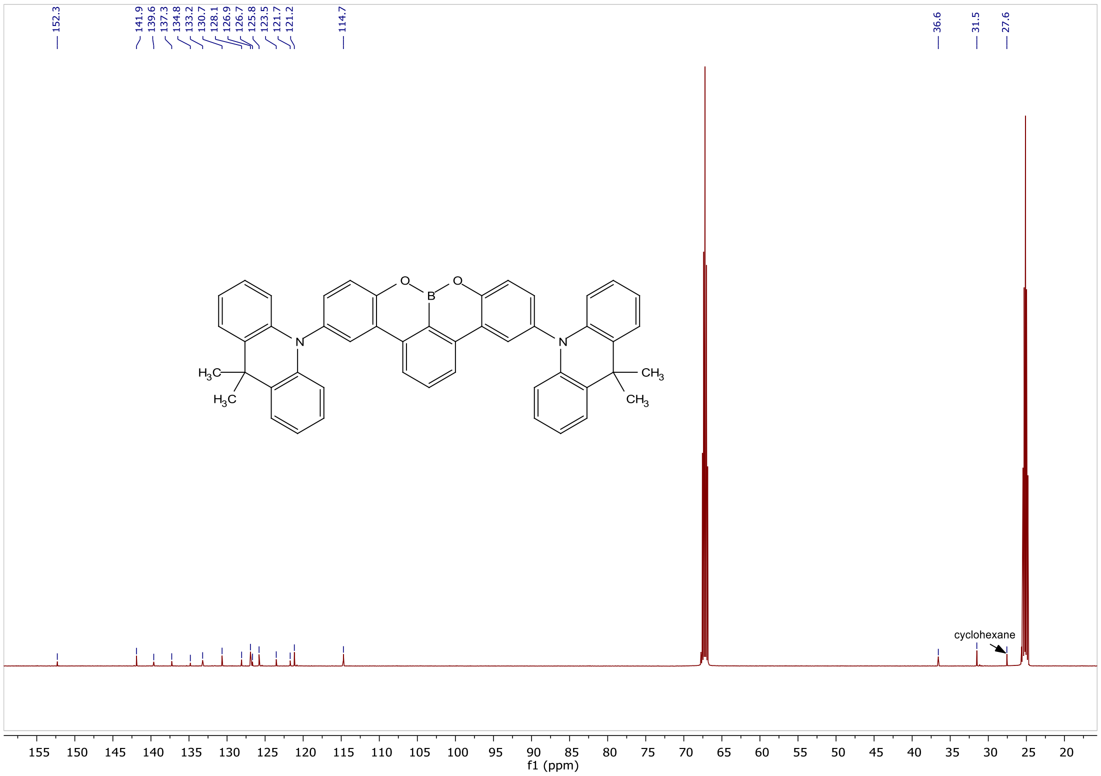

5,12-di(10*N*-phenoxazin-10-yl)-8,9-dioxa-8a-borabenzo[fg]tetracene (DPXZ-OBO)

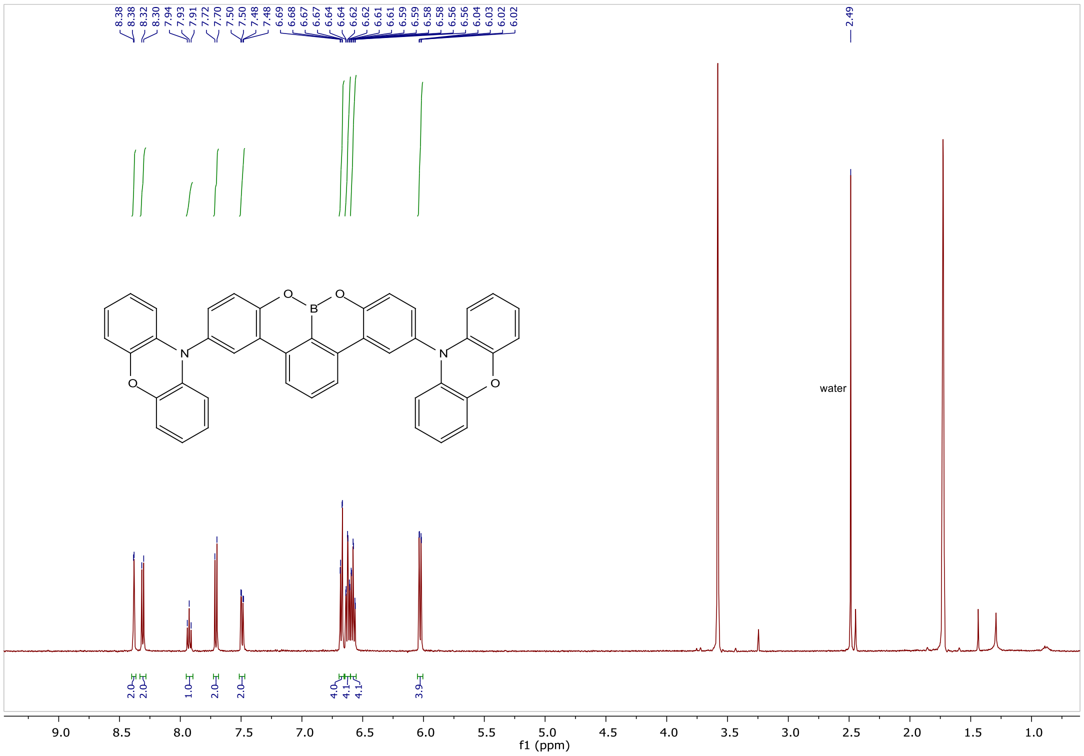

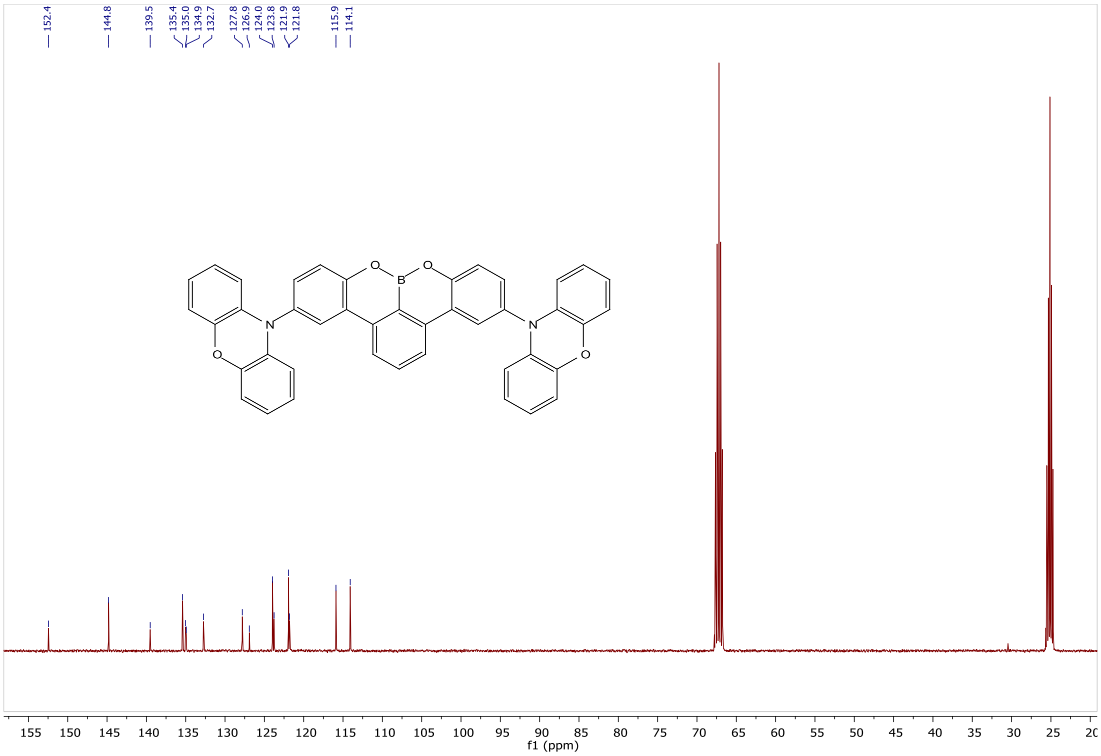

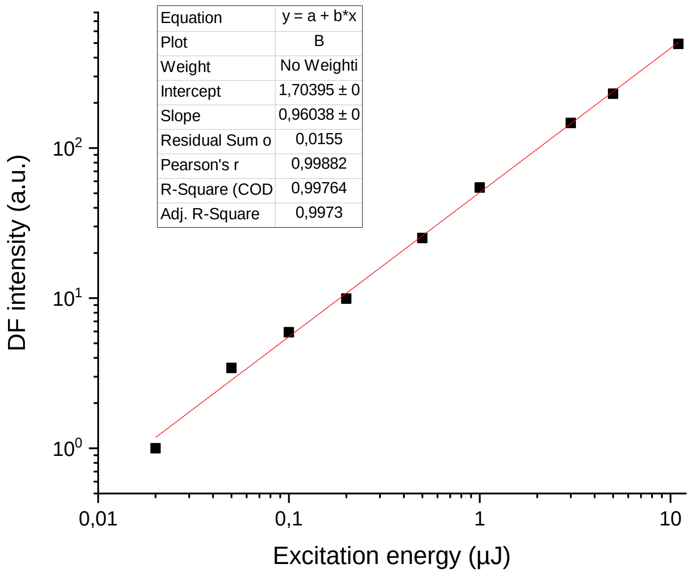

Fig. S4 Dependence of the intensity of delayed emission of **PXZ-OBO** in mCP film measured in the time range 1-100 µs on the intensity of the laser irradiation.

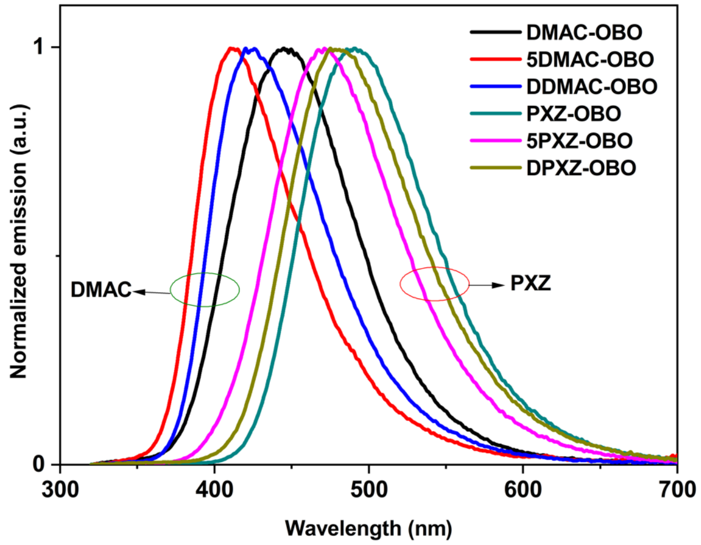

Fig. S5 PL spectra in 10 wt% doped films in PMMA at 300 K.

Table S2 Electronic transitions of UV-vis absorption spectrum calculated by TD-DFT calculations. (LE = locally excited; CT = charge transferred, * = virtual molecular orbitals)

Emitters	Electronic transition	λ_{exc} (nm)	Molecular orbitals associated with electronic transition (%, nature, location)	Oscillator strength (*f*)
DMAC-OBO	S_0-T_1	403.45	H-1→L (77%, LE, OBO)	0.0000
	S_0-T_2	389.56	H→L (97%, CT)	0.0000
	S_0-S_1	384.97	H→L (98%, CT)	0.0004
	S_0-S_3	297.88	H-1→L+1 (78%, LE, OBO)	0.1212
	S_0-S_4	287.84	H-1→L (81%, LE, OBO)	0.2999
	S_0-S_5	270.06	H→L+4* (84%, LE, DMAC)	0.0272
	S_0-S_8	259.27	H-3→L (39%, CT), H→L+5* (50%, LE, DMAC)	0.1217
	S_0-S_9	259.18	H-6→L (15%, CT), H-5→L (24%, LE, OBO), H-4→L+1 (30%, CT)	0.1508
5DMAC-OBO	S_0-T_1	402.92	H-1→L (38%, LE, OBO)	0.0000
	S_0-T_2	384.03	H→L (89%, CT)	0.0000
	S_0-S_1	380.17	H→L (92%, CT)	0.0003
	S_0-S_4	294.35	H-1→L (15%, LE, OBO), H-1→L+1 (67%, LE, OBO)	0.1806
	S_0-S_5	283.38	H-1→L (70%, LE, OBO), H-1→L+1 (16%, LE, OBO)	0.1364
	S_0-S_6	270.44	H→L+4* (70%, CT, DMAC), H→L+5* (16%, CT, DMAC)	0.0554
	S_0-S_9	258.21	H-3→L (23%, LE, DMAC), H→L+6* (65%, LE, DMAC)	0.1907
	S_0-S_{10}	257.81	H-6→L+1 (26%, CT), H-5→L+1 (13%, CT), H-4→L+1 (10%, CT), H-1→L+2 (12%, CT)	0.2283
DDMAC-OBO	S_0-T_1	401.41	H-2→L (77%, LE, OBO)	0.0000
	S_0-T_2	391.95	H-1→L+1 (12%, CT), H→L (83%, CT)	0.0000
	S_0-T_3	391.53	H-1→L (83%, CT), H→L+1 (13%, CT)	0.0000
	S_0-S_1	388.36	H-1→L+1 (11%, CT), H→L (85%, CT)	0.0005
	S_0-S_7	292.96	H-2→L+1 (82%, LE, OBO)	0.2309
	S_0-S_8	284.85	H-2→L (80%, LE, OBO)	0.1174
PXZ-OBO	S_0-T_1	456.31	H→L (93%, CT)	0.0000
	S_0-S_1	446.15	H→L (99%, CT)	0.0005
	S_0-S_2	429.00	H→L+1 (97%, CT)	0.0240
	S_0-S_3	322.48	H→L+2 (95%, LE, PXZ)	0.0234
	S_0-S_4	298.32	H-4→L (11%, LE, OBO), H-1→L+1 (81%, LE, OBO)	0.1159

	S_0-S_5	293.26	H→L+3 (28%, CT), H→L+4 (66%, CT)	0.0141
	S_0-S_6	288.84	H-2→L (26%, CT), H→L+5* (26%, CT), H→L+6* (36%, CT)	0.0846
	S_0-S_7	288.21	H-1→L (80%, LE, OBO)	0.2599
	S_0-S_8	284.88	H→L+3 (67%, CT), H→L+4*(29%, CT)	0.0109
	S_0-S_9	279.88	H-2→L (70%, CT)	0.0979
5PXZ-OBO	S_0-T_1	446.00	H→L (84%, CT)	0.0000
	S_0-S_1	438.08	H→L (93%, CT)	0.0002
	S_0-S_4	320.09	H→L+3 (58%, CT), H→L+4* (35%, CT)	0.0185
	S_0-S_5	294.20	H-1→L+1 (60%, LE, OBO)	0.1666
	S_0-S_6	294.17	H→L+3 (27%, CT), H→L+4* (53%, CT)	0.0617
	S_0-S_7	282.78	H-1→L (59%, LE, OBO), H-1→L+1 (18%, LE, OBO)	0.1379
	S_0-S_8	281.30	H-2→L (38%, CT), H→L+6* (35%, CT)	0.0114
	S_0-S_9	278.21	H-2→L (49%, CT), H→L+6 (36%, CT)	0.1053
DPXZ-OBO	S_0-T_1	462.00	H→L (80%, CT)	0.0000
	S_0-T_2	461.64	H-1→L (79%, CT)	0.0000
	S_0-S_1	455.49	H→L (86%, CT)	0.0003
	S_0-S_8	322.57	H-1→L+3 (34%, CT), H→L+5 (33%, CT)	0.0253
	S_0-S_{10}	304.01	H-1→L+3 (51%, CT)	0.0665

Table S3 Molecular orbitals of the emitters

**DMAC-OBO**	**5DMAC-OBO**	**DDMAC-OBO**
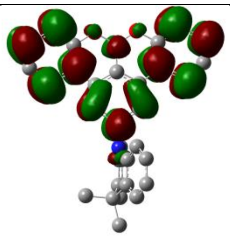 LUMO+3 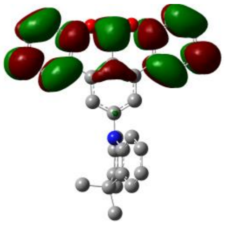 LUMO+2 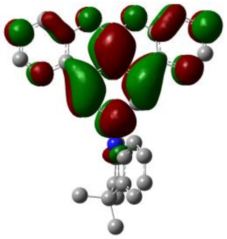	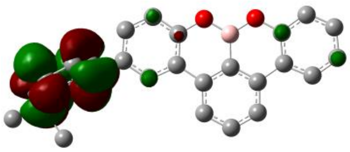 LUMO+6* 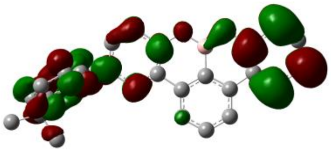 LUMO+5* 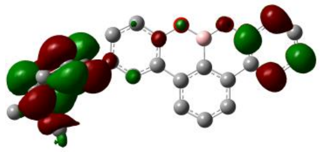 LUMO+4* 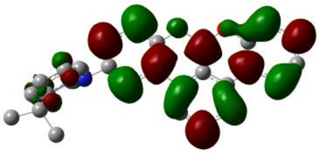 LUMO+3	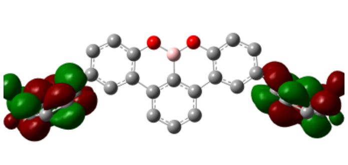 LUMO+6* 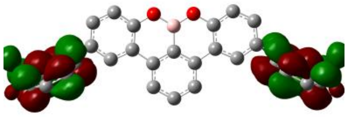 LUMO+5* 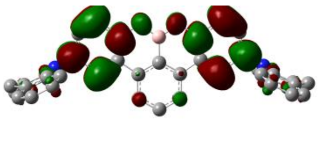 LUMO+4 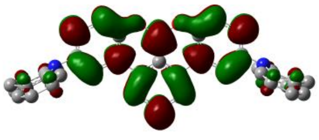 LUMO+3

LUMO+1

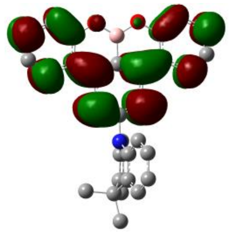

LUMO

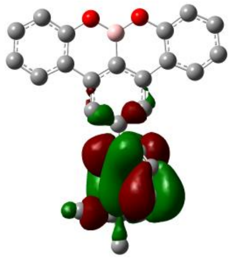

HOMO

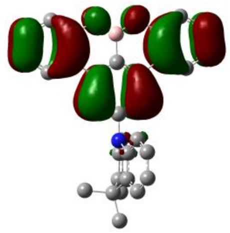

HOMO-1

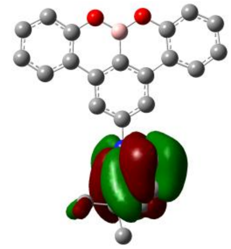

HOMO-2

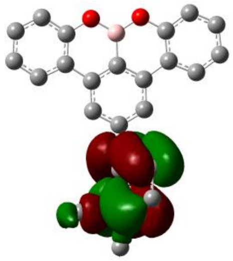

HOMO-3

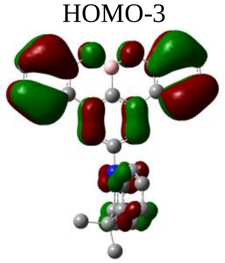

HOMO-4

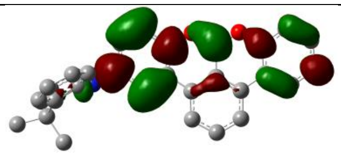

LUMO+2

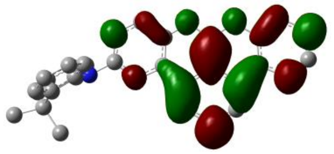

LUMO+1

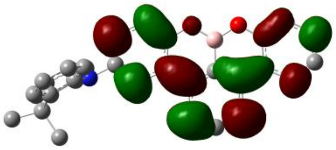

LUMO

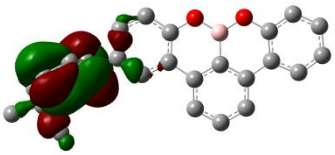

HOMO

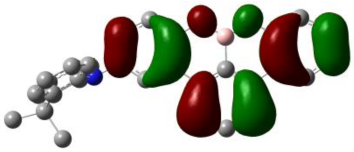

HOMO-1

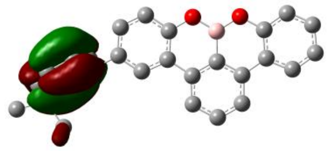

HOMO-2

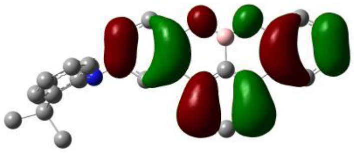

HOMO-3

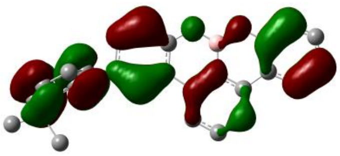

HOMO-4

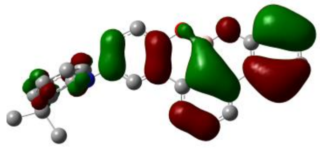

HOMO-5

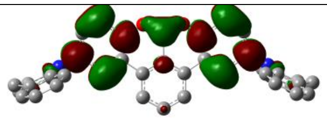

LUMO+2

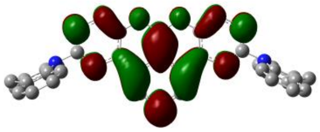

LUMO+1

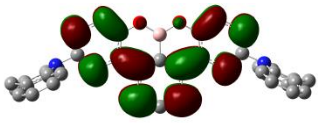

LUMO

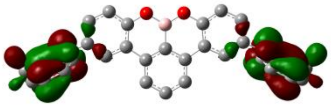

HOMO

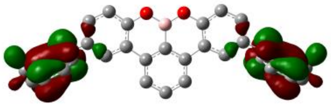

HOMO-1

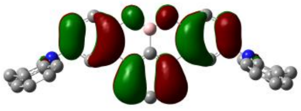

HOMO-2

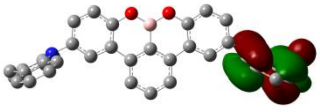

HOMO-3

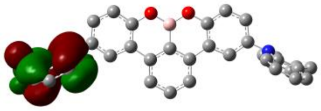

HOMO-4

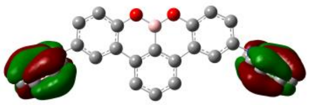

HOMO-5

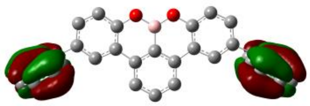

HOMO-6

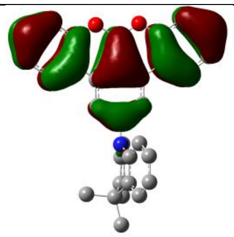<br>HOMO-5<br>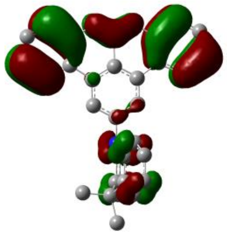<br>HOMO-6	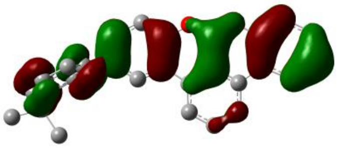<br>HOMO-6<br>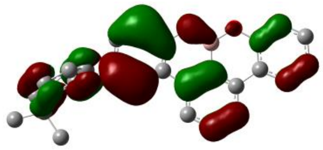<br>HOMO-7<br>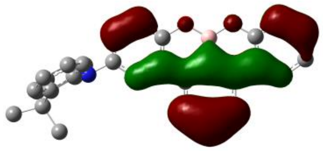<br>HOMO-8	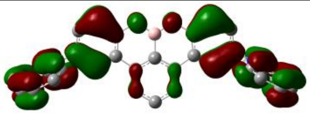<br>HOMO-7<br>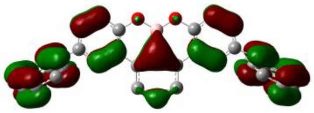<br>HOMO-8<br>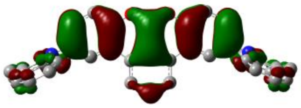<br>HOMO-9<br>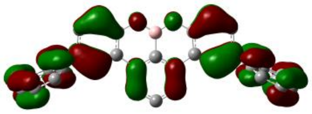<br>HOMO-10
**PXZ-OBO**	**5PXZ-OBO**	**DPXZ-OBO**
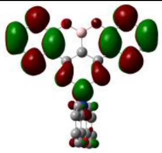<br>LUMO+4<br>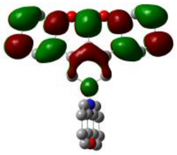<br>LUMO+3<br>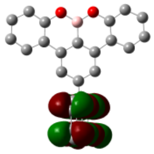<br>LUMO+2<br>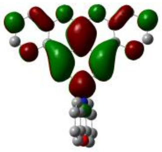<br>LUMO+1<br>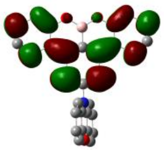<br>LUMO	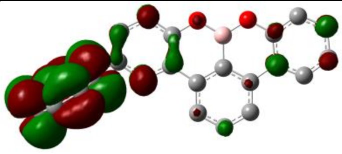<br>LUMO+6*<br>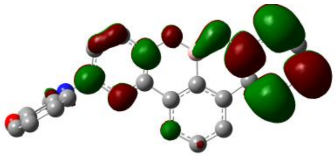<br>LUMO+5*<br>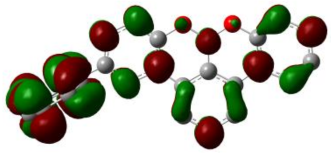<br>LUMO+4*<br>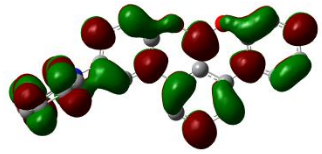<br>LUMO+3<br>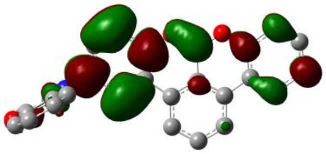<br>LUMO+2	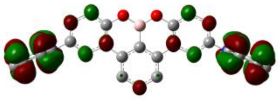<br>LUMO+6<br>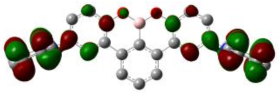<br>LUMO+5<br>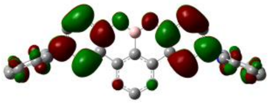<br>LUMO+4<br>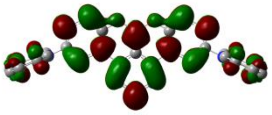<br>LUMO+3<br>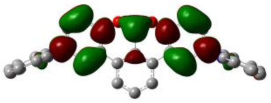<br>LUMO+2

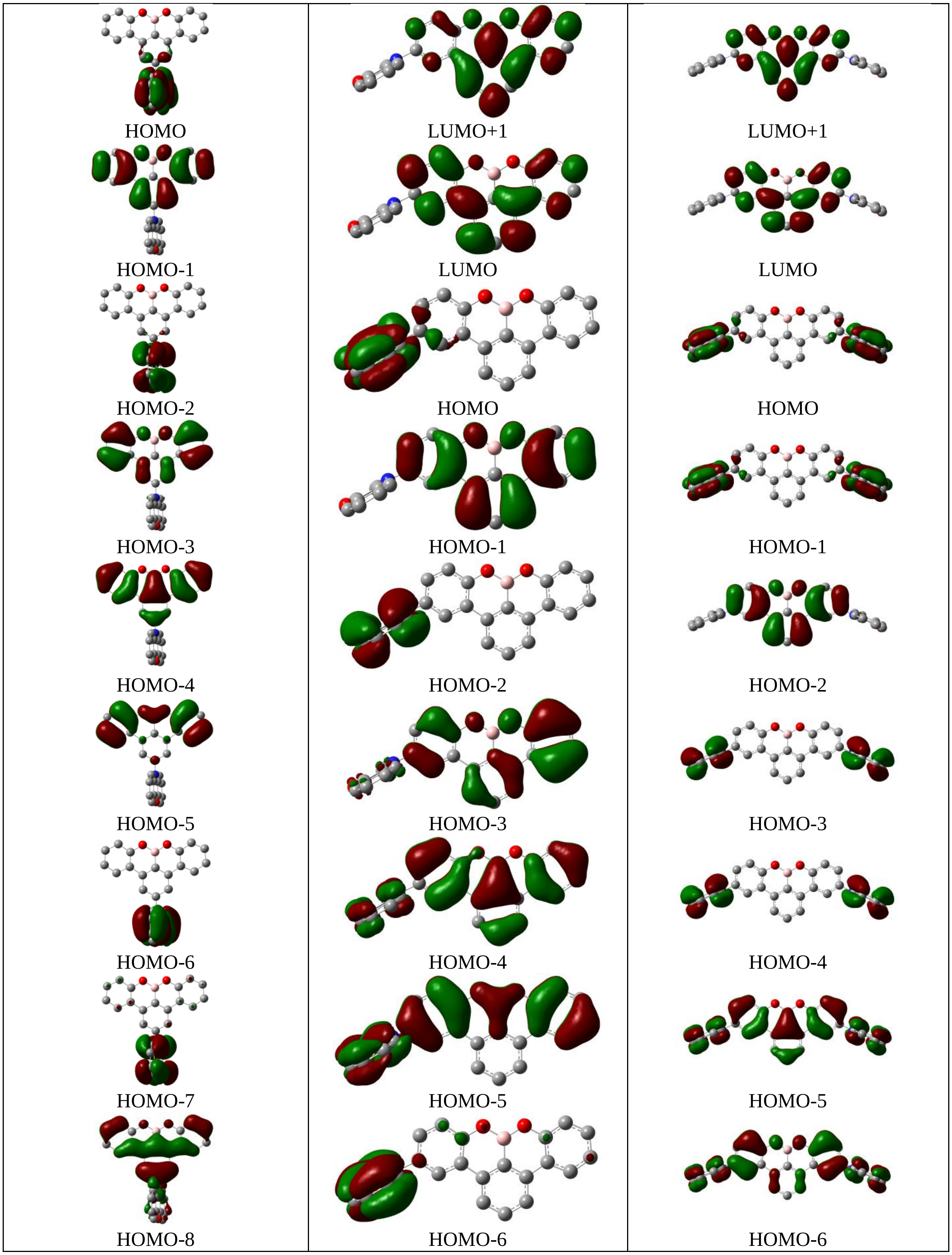
HOMO
HOMO-1
HOMO-2
HOMO-3
HOMO-4
HOMO-5
HOMO-6
HOMO-7
HOMO-8
LUMO+1
LUMO
HOMO
HOMO-1
HOMO-2
HOMO-3
HOMO-4
HOMO-5
HOMO-6
LUMO+1
LUMO
HOMO
HOMO-1
HOMO-2
HOMO-3
HOMO-4
HOMO-5
HOMO-6

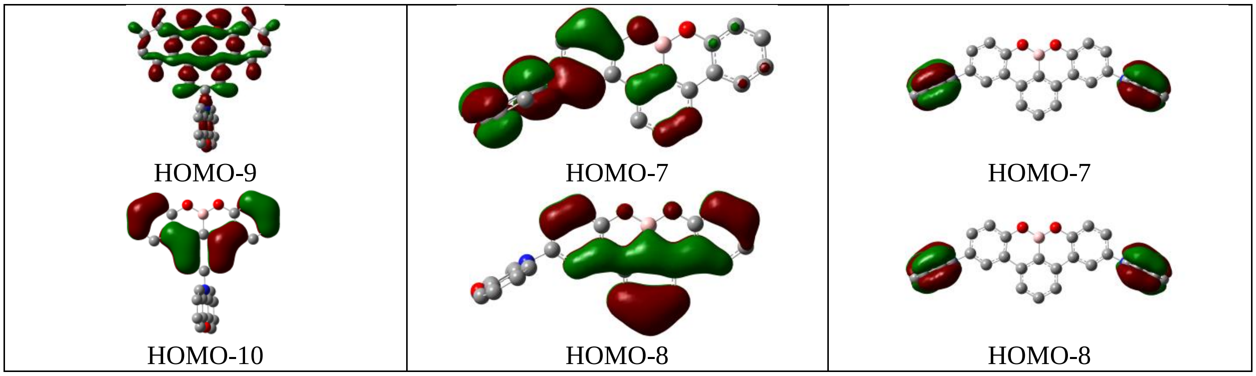

Cartesian coordinates of optimized geometry in ground state

PXZ-OBO

C	4.9699	10.654	12.1432
H	5.1157	11.5171	11.7736
C	3.9321	10.4613	13.0515
H	3.3694	11.19	13.2889
C	3.7171	9.2096	13.6123
H	2.9997	9.0747	14.2203
C	4.5583	8.1577	13.2776
H	4.4285	7.3027	13.6713
C	5.583	8.3526	12.3739
O	6.3662	7.2531	12.0666
C	7.4663	7.4514	11.248
C	8.3224	6.3823	11.0776
H	8.1551	5.5633	11.5295
C	9.4269	6.5038	10.2468
H	10.0205	5.7707	10.133
C	9.6596	7.6994	9.5819
H	10.4072	7.7822	9.0014
C	8.8006	8.7731	9.7651
H	8.9684	9.5893	9.3101
C	7.7002	8.6722	10.6031
N	6.8199	9.743	10.8278
C	5.7949	9.6009	11.7714
C	6.8202	10.8847	9.9541
C	7.7611	11.8892	10.1456
H	8.4166	11.8114	10.828
C	7.7281	13.02	9.3143
C	6.7248	13.106	8.3437
C	5.7844	12.0853	8.1391
C	5.8551	10.951	8.956
H	5.2474	10.2316	8.8296
C	8.6868	14.1338	9.4031
C	9.7899	14.1161	10.2629
H	9.9165	13.3665	10.8319
C	10.7025	15.1539	10.3123
H	11.4367	15.1178	10.9133

C	10.5347	16.2534	9.4723
H	11.162	16.9659	9.4936
C	9.4578	16.3097	8.6085
H	9.3449	17.0609	8.0377
C	8.5406	15.2694	8.5737
O	7.5003	15.4075	7.6783
B	6.6144	14.3779	7.5145
O	5.6209	14.5333	6.5919
C	4.6937	13.5297	6.4156
C	3.6724	13.7975	5.515
H	3.6361	14.6402	5.0781
C	2.7082	12.8434	5.2501
H	2.0081	13.031	4.6363
C	2.7668	11.6034	5.8889
H	2.1172	10.938	5.6969
C	3.7737	11.3472	6.8006
H	3.8012	10.5014	7.2316
C	4.7571	12.3013	7.1099

5PXZ-OBO

C	-2.30409	-1.65026	-0.48066
C	-2.21992	-2.96086	-0.96903
C	-3.5438	-0.98921	-0.47615
C	-3.36916	-3.60122	-1.46886
C	-4.68824	-1.62502	-0.98152
H	-3.61643	-0.00066	-0.09032
C	-4.60081	-2.93127	-1.47936
H	-3.30732	-4.60365	-1.84614
H	-5.62774	-1.11305	-0.99018
H	-5.47175	-3.41828	-1.86217
C	0.06344	-1.43045	-0.72702
C	1.14015	-0.57101	-0.95397
C	0.10758	-2.75506	-1.20157
C	2.26752	-1.0344	-1.64831
H	1.10752	0.44173	-0.59764
C	1.23206	-3.20841	-1.90203
C	2.31664	-2.35313	-2.11984
H	3.09635	-0.37929	-1.81137
H	1.26583	-4.21229	-2.27159
H	3.18477	-2.70229	-2.64323
N	-1.10601	-0.95108	0.01763
C	-0.8423	-1.23601	1.4435
C	-0.44961	-0.21962	2.34392
C	-0.97069	-2.54185	1.89021
C	-0.18211	-0.53698	3.69204
H	-0.35209	0.79494	2.00992
C	-0.68761	-2.8645	3.21653
H	-1.27939	-3.29826	1.20196
C	-0.2999	-1.84866	4.153
H	0.12408	0.22742	4.3674
C	-0.75958	-4.3322	3.63869

C	-0.74001	-5.40232	2.72984
C	-0.80504	-4.58358	4.95928
C	-0.61917	-6.71786	3.22194
H	-0.79337	-5.22383	1.67854
C	-0.8367	-5.84219	5.46128
C	-0.65208	-6.94285	4.61199
H	-0.50446	-7.54312	2.5436
H	-0.5468	-7.93262	5.00738
C	-1.14031	-5.98319	6.96062
C	-1.44577	-4.84211	7.77684
C	-1.14256	-7.26197	7.52075
C	-1.5962	-5.01502	9.15133
C	-1.37461	-7.42002	8.89412
H	-0.97942	-8.12324	6.89764
C	-1.5578	-6.29145	9.71361
H	-1.74317	-4.15987	9.77643
H	-1.41379	-8.40183	9.31485
H	-1.68044	-6.40647	10.76475
B	-0.82014	-3.35346	5.9665
O	-0.01951	-2.12151	5.56507
O	-1.63288	-3.49121	7.24675
O	-0.97772	-3.65851	-0.9753

DPXZ-OBO

C	0.77997	-4.60466	4.43699
C	2.11606	-4.49646	4.659
C	2.82161	-3.35763	4.43532
C	2.17227	-2.24824	3.88123
C	0.76844	-2.29619	3.71312
C	0.05755	-3.47648	4.02999
H	2.72419	-1.37438	3.60666
H	0.24449	-1.43838	3.34552
H	-1.00968	-3.51124	3.94464
C	4.30572	-3.3629	4.86434
C	5.13838	-2.31531	4.45103
C	4.83336	-4.41482	5.69318
C	6.48513	-2.29303	4.82696
H	4.74322	-1.5279	3.84534
C	6.19213	-4.3796	6.04129
C	7.01273	-3.32518	5.61305
H	6.60632	-5.16296	6.64019
H	8.04646	-3.30912	5.88883
C	0.1589	-6.00799	4.60957
C	-1.23404	-6.13698	4.61423
C	0.98825	-7.17546	4.74826
C	-1.82664	-7.39272	4.78678
H	-1.84876	-5.27136	4.48803
C	0.37157	-8.42221	4.93012
C	-1.02646	-8.53107	4.95421
H	0.97367	-9.29775	5.05043
H	-1.4834	-9.48795	5.09876

B	2.8935	-5.76796	5.21
O	2.45168	-7.13563	4.70482
O	4.02324	-5.52484	6.2026
N	7.34391	-1.17998	4.39355
N	-3.29272	-7.51046	4.79317
C	8.00644	-1.52902	3.12975
C	9.2522	-2.17455	3.13637
C	7.38909	-1.21394	1.91043
C	9.88	-2.49191	1.92404
C	8.01955	-1.53265	0.69979
H	6.43487	-0.72976	1.90411
C	9.26652	-2.16961	0.70773
H	10.83102	-2.9813	1.92806
H	7.54872	-1.28905	-0.2299
H	9.75104	-2.41092	-0.21509
C	8.35347	-0.91317	5.42378
C	8.07162	0.00968	6.44016
C	9.59003	-1.57263	5.39808
C	9.02169	0.27061	7.43535
H	7.12875	0.51488	6.45524
C	10.5413	-1.30734	6.39332
C	10.25676	-0.38806	7.41185
H	8.80442	0.97298	8.21279
H	11.48659	-1.80821	6.37506
H	10.98334	-0.18966	8.17153
C	-3.76435	-7.80023	3.43326
C	-3.88454	-9.12707	2.99257
C	-4.09376	-6.7442	2.57095
C	-4.33031	-9.39079	1.69006
C	-4.54194	-7.01201	1.27016
H	-4.00303	-5.73202	2.90653
C	-4.6592	-8.33595	0.82993
H	-4.41895	-10.40117	1.3515
H	-4.79428	-6.20578	0.61347
H	-5.00051	-8.5422	-0.16331
C	-3.69599	-8.59229	5.69754
C	-3.95713	-8.30286	7.04386
C	-3.82053	-9.90608	5.22586
C	-4.34196	-9.32448	7.92048
H	-3.86239	-7.29893	7.40202
C	-4.20877	-10.92705	6.1045
C	-4.46874	-10.63687	7.45059
H	-4.53837	-9.10237	8.94863
H	-4.30648	-11.93075	5.74628
H	-4.76424	-11.41792	8.11913
O	-3.55435	-10.22222	3.85528
O	9.89609	-2.51887	4.36818

DMAC-OBO

C	0.02788	0.27764	-0.54992
C	0.03508	-1.0338	-0.04272

C	0.05712	-1.23795	1.34371
C	0.10764	-0.1529	2.18885
C	0.05298	1.14089	1.71551
C	0.02511	1.3712	0.33369
H	0.02179	-1.86983	-0.71036
H	0.00088	2.37127	-0.04601
C	0.00886	-2.65274	1.9506
C	0.06913	-2.87446	3.35735
C	-0.11769	-3.74365	1.07864
C	-0.10635	-4.1791	3.84517
C	-0.25362	-5.04117	1.58325
H	-0.11569	-3.58201	0.02091
C	-0.26616	-5.2561	2.96448
H	-0.11271	-4.35318	4.9009
H	-0.35085	-5.86877	0.91208
H	-0.39557	-6.24596	3.3496
C	0.00069	2.30032	2.73076
C	-0.22084	3.59926	2.2518
C	0.1564	2.08051	4.12858
C	-0.36666	4.66646	3.14541
H	-0.28473	3.77458	1.19819
C	-0.03128	3.15394	5.01244
C	-0.29212	4.44044	4.52394
H	-0.53821	5.65522	2.77411
H	0.03132	2.98961	6.06789
H	-0.43163	5.25257	5.20648
B	0.2228	-0.39849	3.776
O	0.52208	0.7985	4.69545
O	0.30995	-1.8301	4.33694
C	-1.29429	0.59858	-3.08658
C	-1.29782	-0.44242	-4.00951
C	-2.31255	1.5461	-3.01675
C	-2.45188	-0.66939	-4.76726
C	-3.44772	1.35797	-3.82008
H	-2.23584	2.38877	-2.36184
C	-3.53007	0.23343	-4.6697
H	-2.51172	-1.51408	-5.42135
H	-4.25155	2.06333	-3.7854
H	-4.41531	0.06796	-5.24749
C	1.32056	0.61767	-3.09986
C	2.32545	1.5801	-3.04023
C	1.33012	-0.4231	-4.02291
C	3.45494	1.40898	-3.8553
H	2.24306	2.42145	-2.38431
C	2.47963	-0.63294	-4.79249
C	3.5452	0.28582	-4.70593
H	4.24849	2.12625	-3.8288
H	2.54535	-1.47671	-5.44721
H	4.42676	0.13359	-5.29291
C	0.02178	-1.31682	-4.19487
N	0.01825	0.53279	-2.18006
C	0.03547	-2.46103	-3.16425

H	0.9091	-2.37907	-2.55191
H	-0.8381	-2.3979	-2.54961
H	0.04493	-3.40114	-3.67515
C	0.01882	-1.90162	-5.61951
H	-0.85127	-2.50979	-5.7536
H	0.00969	-1.10385	-6.33252
H	0.89597	-2.49755	-5.76226

5DMAC-OBO

C	-0.42566	0.89424	-3.56904
C	-0.79163	-0.45226	-3.43148
C	-1.41042	1.86512	-3.79891
C	-2.13935	-0.82562	-3.52531
C	-2.75759	1.49134	-3.8924
H	-1.13286	2.89321	-3.90321
C	-3.12211	0.1458	-3.75582
H	-2.41783	-1.85344	-3.42073
H	-3.50784	2.23371	-4.06813
H	-4.15076	-0.13997	-3.82747
C	1.91657	0.26897	-3.961
C	3.12501	0.65429	-4.558
C	1.60437	-1.09188	-3.83224
C	4.01896	-0.31781	-5.02616
H	3.36506	1.69237	-4.65636
C	2.49889	-2.06383	-4.30087
C	3.70583	-1.67703	-4.89774
H	4.94077	-0.02186	-5.48176
H	2.25976	-3.10213	-4.20261
H	4.3882	-2.41959	-5.25533
C	0.28181	-1.52389	-3.1767
N	0.98607	1.2999	-3.47308
C	1.305	1.61608	-2.07339
C	1.70673	2.90932	-1.72255
C	1.20302	0.62383	-1.09173
C	2.04706	3.2043	-0.39627
H	1.75707	3.67389	-2.46941
C	1.50252	0.91808	0.24589
H	0.88919	-0.36263	-1.36248
C	1.98819	2.2105	0.59253
H	2.36022	4.19385	-0.13624
C	1.27784	-0.17698	1.30795
C	0.91952	-1.48403	0.95239
C	1.4151	0.15272	2.63962
C	0.68001	-2.43404	1.96072
H	0.82785	-1.75667	-0.07823
C	1.13432	-0.74656	3.64292
C	0.77918	-2.06162	3.31299
H	0.41973	-3.43847	1.69943
H	0.58305	-2.77654	4.08456
C	1.20269	-0.27373	5.10719
C	1.59857	1.05029	5.45643

C	0.84267	-1.18111	6.11389
C	1.5171	1.44904	6.79998
C	0.80402	-0.77526	7.45198
H	0.59081	-2.18865	5.85637
C	1.12309	0.54258	7.79203
H	1.76382	2.45468	7.06962
H	0.5275	-1.47272	8.21485
H	1.06864	0.85867	8.81282
B	1.90742	1.63545	3.0292
O	2.44976	2.5557	1.9216
O	2.09473	2.02952	4.5061
C	-0.17706	-2.86328	-3.78253
H	-1.09713	-3.16431	-3.32673
H	-0.32231	-2.74699	-4.83623
H	0.56943	-3.60915	-3.60556
C	0.49088	-1.69111	-1.66017
H	-0.14039	-1.00489	-1.13529
H	1.51374	-1.49225	-1.41707
H	0.24455	-2.6924	-1.37446

DDMAC-OBO

C	0.8277	-4.67541	3.86116
C	2.15856	-4.57635	4.12517
C	2.88624	-3.44056	3.93478
C	2.27938	-2.34295	3.30419
C	0.88741	-2.39877	3.03837
C	0.14692	-3.56273	3.35565
H	2.84923	-1.47636	3.04405
H	0.39347	-1.55572	2.60114
H	-0.90911	-3.60048	3.19803
C	4.33438	-3.44495	4.49566
C	5.18065	-2.35302	4.24651
C	4.81978	-4.54592	5.29333
C	6.49767	-2.35851	4.73653
H	4.81788	-1.51429	3.69102
C	6.15964	-4.55947	5.69527
C	6.99725	-3.4803	5.4079
H	6.54124	-5.40183	6.23365
H	8.02489	-3.51042	5.70464
C	0.17089	-6.05112	4.12466
C	-1.22919	-6.14957	4.19297
C	0.98291	-7.22515	4.30391
C	-1.82797	-7.37002	4.53193
H	-1.83849	-5.2897	4.00161
C	0.35638	-8.43965	4.61131
C	-1.03811	-8.50968	4.73603
H	0.95081	-9.3199	4.75727
H	-1.50508	-9.43802	4.98927
B	2.89276	-5.86378	4.69924
O	2.44047	-7.21809	4.18876
O	3.99006	-5.66272	5.72447

N	7.36041	-1.18333	4.57475
N	-3.28603	-7.46	4.69761
C	8.0941	-1.24937	3.29603
C	9.34554	-1.87707	3.19406
C	7.51882	-0.6631	2.15994
C	10.01919	-1.90686	1.96695
C	8.19174	-0.69878	0.93187
H	6.56486	-0.1853	2.23362
C	9.44362	-1.31624	0.8367
H	10.97417	-2.38315	1.89367
H	7.74753	-0.25193	0.06672
H	9.95963	-1.33641	-0.09981
C	8.28043	-1.15241	5.72883
C	7.88222	-0.47852	6.89281
C	9.53104	-1.78845	5.68809
C	8.71938	-0.4525	8.01609
H	6.9354	0.01392	6.92422
C	10.37335	-1.74983	6.80809
C	9.96482	-1.08768	7.9744
H	8.40661	0.05315	8.90617
H	11.33055	-2.22834	6.77247
H	10.60488	-1.06946	8.83149
C	-3.91967	-7.95856	3.45491
C	-4.10971	-9.3343	3.23147
C	-4.34266	-7.0407	2.4826
C	-4.73085	-9.78255	2.05894
C	-4.96455	-7.49141	1.30851
H	-4.19096	-5.9939	2.637
C	-5.16342	-8.86214	1.09988
H	-4.87426	-10.8308	1.8981
H	-5.2876	-6.78749	0.57248
H	-5.64588	-9.20474	0.20813
C	-3.54746	-8.35557	5.83434
C	-3.62058	-7.79464	7.11602
C	-3.71204	-9.73556	5.66683
C	-3.83981	-8.60964	8.2313
H	-3.50575	-6.73854	7.24155
C	-3.94077	-10.55231	6.78316
C	-3.99832	-9.99045	8.06647
H	-3.88499	-8.17762	9.20951
H	-4.06907	-11.60755	6.65612
H	-4.16299	-10.61502	8.91965
C	-3.6413	-10.35978	4.26853
C	9.98651	-2.53219	4.42235
C	-2.18937	-10.77122	3.96159
H	-1.7643	-10.08338	3.26082
H	-1.61663	-10.75928	4.86532
H	-2.17838	-11.75689	3.54536
C	-4.54818	-11.60318	4.21251
H	-5.55738	-11.31846	4.42543
H	-4.49854	-12.03712	3.23571
H	-4.21872	-12.31789	4.93745

C	9.54808	-4.00619	4.50423
H	9.27103	-4.2405	5.51083
H	8.71071	-4.16569	3.8575
H	10.35789	-4.63652	4.20127
C	11.52028	-2.45773	4.30571
H	11.836	-2.97029	3.42111
H	11.82459	-1.43332	4.25217
H	11.96591	-2.91786	5.16279

Reference

[1] M. J. Frisch, G. W. Trucks, H. B. Schlegel, G. E. Scuseria, M. A. Robb, J. R. Cheeseman, G. Scalmani, V. Barone, G. A. Petersson, H. Nakatsuji, X. Li, M. Caricato, A. V. Marenich, J. Bloino, B. G. Janesko, R. Gomperts, B. Mennucci, H. P. Hratchian, J. V. Ortiz, A. F. Izmaylov, J. L. Sonnenberg, Williams, F. Ding, F. Lipparini, F. Egidi, J. Goings, B. Peng, A. Petrone, T. Henderson, D. Ranasinghe, V. G. Zakrzewski, J. Gao, N. Rega, G. Zheng, W. Liang, M. Hada, M. Ehara, K. Toyota, R. Fukuda, J. Hasegawa, M. Ishida, T. Nakajima, Y. Honda, O. Kitao, H. Nakai, T. Vreven, K. Throssell, J. A. Montgomery Jr., J. E. Peralta, F. Ogliaro, M. J. Bearpark, J. J. Heyd, E. N. Brothers, K. N. Kudin, V. N. Staroverov, T. A. Keith, R. Kobayashi, J. Normand, K. Raghavachari, A. P. Rendell, J. C. Burant, S. S. Iyengar, J. Tomasi, M. Cossi, J. M. Millam, M. Klene, C. Adamo, R. Cammi, J. W. Ochterski, R. L. Martin, K. Morokuma, O. Farkas, J. B. Foresman, D. J. Fox, Wallingford, CT, **2009**.

[2] C. Adamo, V. Barone, *J. Chem. Phys.* **1999**, *110*, 6158-6170.

[3] W. J. Hehre, R. Ditchfield, J. A. Pople, *J. Chem. Phys.* **1972**, *56*, 2257-2261.

[4] S. Hirata, M. Head-Gordon, *Chem. Phys. Lett.* **1999**, *314*, 291-299.

[5] X. Y. Wang, A. Narita, W. Zhang, X. Feng, K. Mullen, *J. Am. Chem. Soc.* **2016**, *138*, 9021-9024.

[6] G. M. Sheldrick, *Acta. Crystallogr. A* **2015**, *71*, 3-8.

[7] G. M. Sheldrick, *Acta. Crystallogr. C* **2015**, *71*, 3-8.
